# Discovery of Potent PDE4 Inhibitors with 3(2H)-Pyridazinone Scaffold: Synthesis, In Silico Studies and In Vitro/Vivo Evaluation

**DOI:** 10.3390/molecules31040699

**Published:** 2026-02-17

**Authors:** Claudia Vergelli, Letizia Crocetti, Gabriella Guerrini, Fabrizio Melani, Jordi Gracia, Maria Antonia Buil, Yolanda Garrido, Lluis Pagès, Joan Taltavull, Amadeu Gavaldà, Elena Calama, Maria Paola Giovannoni

**Affiliations:** 1 Neurofarba, Pharmaceutical and Nutraceutical Section, University of Florence, Via Ugo Schiff 6, 50019 Sesto Fiorentino, Italy; letizia.crocetti@unifi.it (L.C.); gabriella.guerrini@unifi.it (G.G.); fabrizio.melani54@gmail.com (F.M.); mariapaola.giovannoni@unifi.it (M.P.G.); 2 Medicinal Chemistry Department, Evotec (France) SAS, Campus Curie, 195, Route d‘Espagne, 31036 Toulouse CEDEX, France; jordi.gracia@evotec.com; 3 New Chemical Entities Discovery & Early Development, Almirall R&D, Carrer Laureà Miró 408-410, 08980 Sant Feliu de Llobregat, Spain; mantoniabuil@gmail.com (M.A.B.); ygru03@gmail.com (Y.G.); lluis.pages@almirall.com (L.P.); joantaltavull@yahoo.com (J.T.); 4 Pharmacology, Almirall R&D, Carrer Laureà Miró 408-410, 08980 Sant Feliu de Llobregat, Spain; amadeu.gavalda@almirall.com (A.G.); elena.calama@almirall.com (E.C.)

**Keywords:** phosphodiesterases 4, inhibition, selectivity, in vivo test, docking

## Abstract

Phospodiesterase 4 (PDE4) has long been an attractive target not only for the anti-inflammatory therapy in respiratory diseases, but also for other pathologies such as psoriatic arthritis and atopic dermatitis. In this study, we report the synthesis of 5-acetyl-2-ethyl-6-phenyl-3(2H)-pyridazinones differently substituted at position 4 with a variety of aryl/alkylamines, which act as potent PDE4B1 inhibitors in the low nanomolar range. The selectivity toward PDE4A4, PDE4D3 and HARBS, as well as the ability to inhibit TNFα production in human whole blood (hWB), was also evaluated for the most potent products, resulting in a small cluster of compounds with an interesting profile and two selected products (**3a** and **3k**) have been in depth investigated with additional in vitro tests on metabolism and in vivo studies. Finally, molecular docking and minimization of the ligand-enzyme complexes were carried out.

## 1. Introduction

Phosphodiesterases (PDEs) are a family of enzymes that catalyze the hydrolysis of the secondary messengers cyclic adenosine monophosphate (c-AMP) and cyclic guanosine monophosphate (c-GMP) to the inactive 5′-AMP and 5′-GMP nucleotides. Due to their ability to regulate the levels of these essential second messengers, PDEs are involved in many metabolic and physiological processes, such as inflammation, cell differentiation, muscle contraction, apoptosis, and ion channel signaling [1,2,3]. To date, 21 different genes encoding for PDEs have been identified in the human genome; these genes express more than 100 isoforms, which are classified into 11 different families (PDE1-PDE11) characterized by different structures, substrate specificity (c-AMP or c-GMP), sensitivity to synthetic inhibitors, tissues, and cell distribution [4].

cAMP-specific PDE4 family is predominant in humans and abundantly expressed in a variety of cells and tissues; four subtypes have been identified (PDE4A-PDE4D), encoded by four distinct genes (A-D), which produce more than 20 splice variants. PDE4 A, B, and D are highly expressed in inflammatory and immune cells, including neutrophils, T-lymphocytes, B cells, macrophage and eosinophils, and in many tissues such as airway and vascular smooth muscle, vascular endothelium, and brain, whereas PDE4C isoform is mainly found in testis, skeletal muscle, and SNC [5,6].

PDE4s, as above reported, are involved in several physiological processes (such as monocyte and macrophage activation, neutrophil adhesion and migration, airway immunity, brain and cardiovascular functions), and their inhibition, resulting in an increase of intracellular c-AMP concentration, exerts a broad range of anti-inflammatory effects such as inhibition of cytokine and chemokine release, inhibition of reactive oxygen species production in monocytes and macrophages and so on. Additionally, elevation of intracellular c-AMP levels led to smooth muscle relaxation and thereby bronchodilation, beneficial for the management of respiratory diseases [7,8].

Therefore, PDE4 inhibitors are of remarkable interest for the treatment of a variety of inflammatory and autoimmune diseases, including chronic obstructive pulmonary disease (COPD) and asthma, atopic dermatitis, psoriasis, and psoriatic arthritis, rheumatoid arthritis, vascular and neurological disorders, Alzheimer’s disease, mild cognitive impairment, depression, and cancer [9,10,11,12,13].

Since the early 1980s, many PDE4 inhibitors have been studied [14]; despite their high potency in vitro, they showed enormous limitations in vivo, mainly due to their adverse effects that prevented their clinical development. The most common side effects were nausea, diarrhea, abdominal pain, vomiting, and headache, mainly responsible for the lack of correlation between the preclinical and clinical data [9]. The adverse effects were thought to be caused by the inhibition of PDE4 in non-target tissues, especially in the CNS and gut, as well as by the binding at the Rolipram binding site (HARBS) [15]. Finally, high selectivity towards PDE4B isoform with respect to isoform D was also considered an important factor to limit vomiting, as isoform D was indicated as the main contributor to PDE4 inhibitor-induced emesis. However, further studies implied that inhibition of PDE4D may not be a key factor to cause vomiting [9,16].

**Figure 1:**
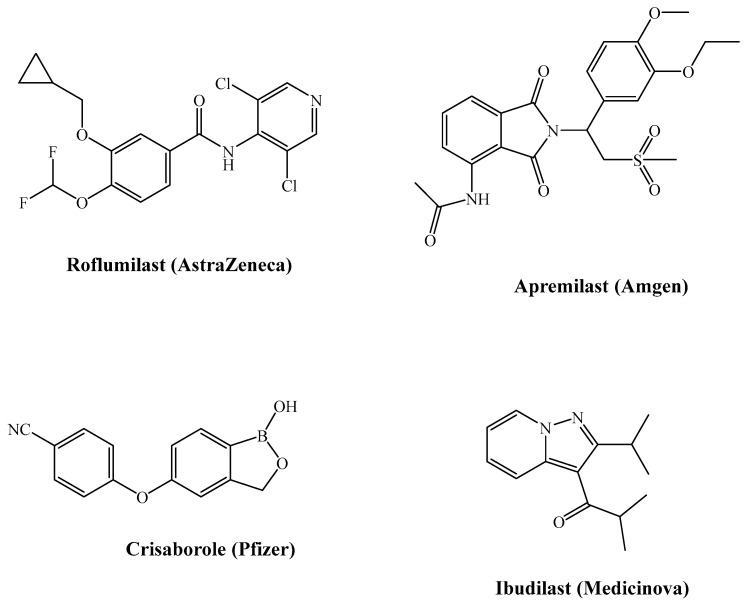
PDE4 inhibitors on the market: Roflumilast, Apremilast, Crisaborole and Ibudilast.

In light of this information, together with considerable advances in the knowledge of the biochemistry and pharmacology of PDE4, new potent and selective compounds were designed [9,10], and the culmination of the research was the discovery of **Roflumilast**, the first marketed PDE4 inhibitor (Figure 1). **Roflumilast** by AstraZeneca [17,18] was approved in the European Union in 2010 (Daxas^®^) and in the US in 2011 (Daliresp^®^) for the treatment of severe COPD associated with chronic bronchitis. The marketing of Roflumilast was followed by three other drugs (Figure 1): **Apremilast** (by Amgen marketed as Otezla^®^) approved in 2014 by FDA in US and in 2015 in EU for psoriatic arthritis in adults [19,20] and in 2019 for oral ulcers associated with Behçet’s Disease (US and Japan) [21]; **Crisaborole** by Pfizer (Eucrisa^®^), the first nonsteroidal drug approved in 2016 for atopic dermatitis [22] and **Ibudilast** by MediciNova, approved, in 2016, for bronchial asthma in Japan and in US for the treatment of Krabbe diseases, a rare and often fatal child pathology resulting in progressive damage to nervous system [23].

**Figure 2:**
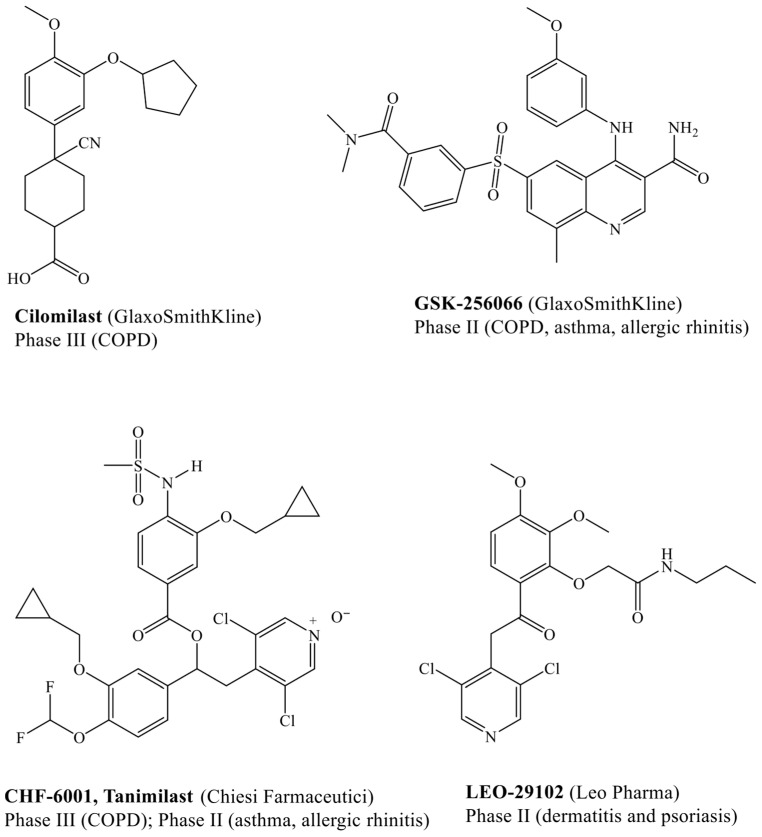
PDE4 inhibitors in clinical trials.

Many PDE4 inhibitors have been or are currently in clinical trials not only for COPD, but also for asthma and atopic dermatitis; some examples are reported in Figure 2. For example, **Cilomilast** (GlaxoSmithKline) in Phase III for COPD and recently discontinued [24,25], **GSK-256066** (GlaxoSmithKline) and **CHF-6001** (Tanimilast, Chiesi Farmaceutici) [26,27,28], the first in phase II for COPD, the second in phase III for treatment of COPD and in phase II for asthma and allergic rhinitis, both available for topical pulmonary administration via inhalation, therefore with reduced adverse effects associated with systemic exposure [29]. Finally, **LEO-29102** is a soft drug used as topic in the treatment of atopic dermatitis and psoriasis plaques (Phase II clinical trials) [6]. In this drug, two strategies (both aimed at decreasing/suppressing adverse effects) have been combined: on the one hand, rapid metabolism, which converts the drug into non-toxic metabolites, and on the other hand, topical administration, which limits systemic exposure [6].

**Figure 3:**
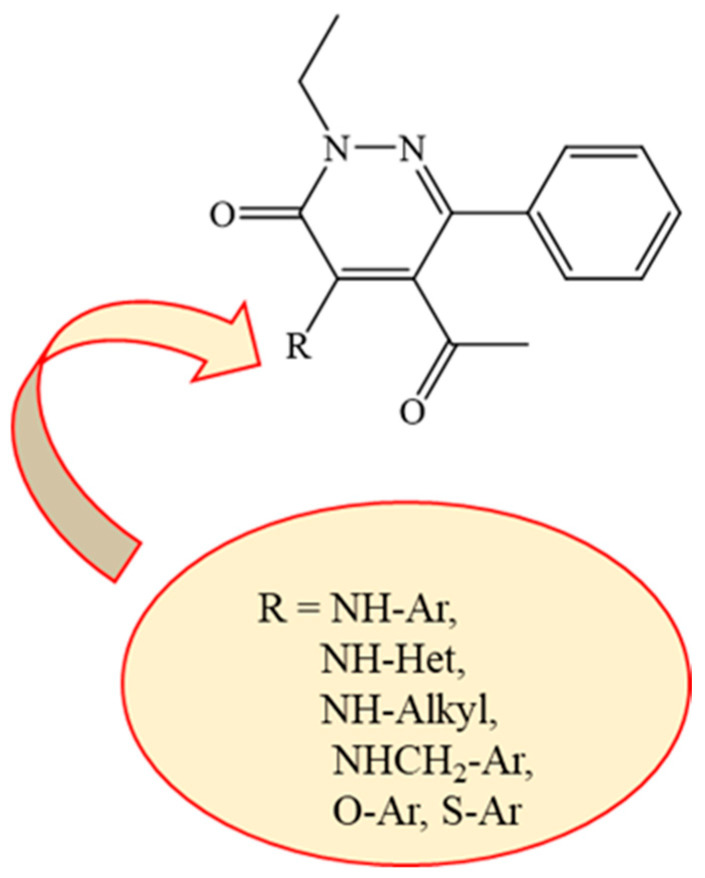
Modifications performed at position 4 of the pyridazinone scaffold.

In the past years, we have worked on the design and synthesis of PDE4 inhibitors with heterocyclic-fused 3(2H)-pyridazinones nuclei [30,31,32], which exhibited interesting activity and selectivity. At the same time, we widely investigated the 3(2H)-pyridazinone nucleus by modifying positions 2, 4, 5, and 6 with a variety of substituents and functions. In this article, we report the first part of this study which regards the modifications performed at position 4 of the scaffold and in particular we describe the synthesis of 5-acetyl-2-ethyl-6-phenyl-3(2H)-pyridazinones extensively substituted at position 4 with a variety of aryl/alkyamines (Figure 3), 4-O- and 4-S-arylderivatives; they have been evaluated as PDE4 inhibitors and for the selected compounds in-depth in vitro and in vivo studies have been performed.

## 2. Results and Discussion

### 2.1. Chemistry

**Scheme 1:**
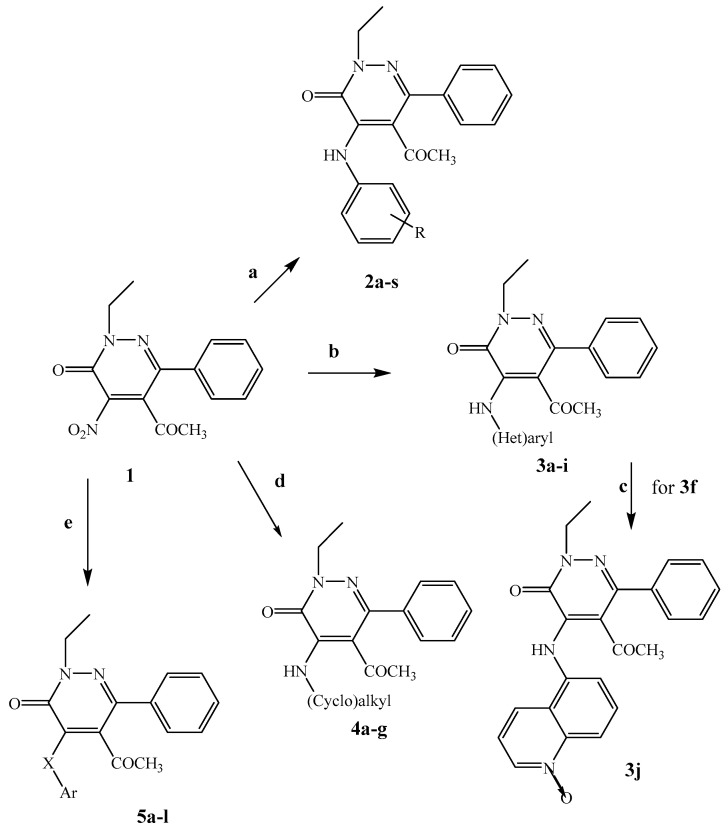
Modifications performed at position 4 of the pyridazinone scaffold. Synthesis of final compounds **2a**-**s**, **3a**-**j**, **4a**-**g** and **5a**-**l**. Reagent and conditions: (a) Appropriate (substituted)aniline, EtOH, r.t., 30 min-3 h (for **2o**: 60 °C, 1 h; for **2p** and **2q**, r.t., 65 h and 20 h respectively). (b) Appropriate (hetero)arylamine, EtOH, r.t., 2-40 h (for **3g**: microwave, 2 h). (c) 3-Chloroperoxybenzoic acid, CH_2_Cl_2_, r.t., 27 h. (d) Appropriate (cyclo)alkylamine, EtOH, r.t., 30 min-4 h. (e) Appropriate (Hetero)arylalkyl amine, EtOH, r.t., 30 min-2 h (for 5d: 90 °C, 3 h).

**Scheme 2:**
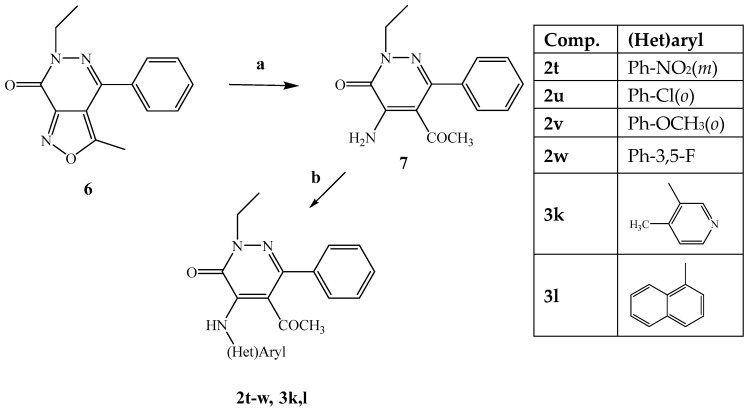
Modifications performed at position 4 of the pyridazinone scaffold. Synthesis of final compounds **2t**-**w** and **3k**,**l**. Reagent and conditions: (a) 10% Pd/C, ammonium formate, EtOH, reflux, 1 h. (b) **2t**-**w** and **3l**: appropriate arylboronic acid, anhydrous cupric acetate, triethylamine, activated molecular sieves, anhydrous CH_2_Cl_2_, r.t., 24 h; for **3k**: 3-bromo-4-methylpyridine, anhydrous cuprous iodide, K_2_CO_3_, 145 °C, 12 h.

**Scheme 3:**
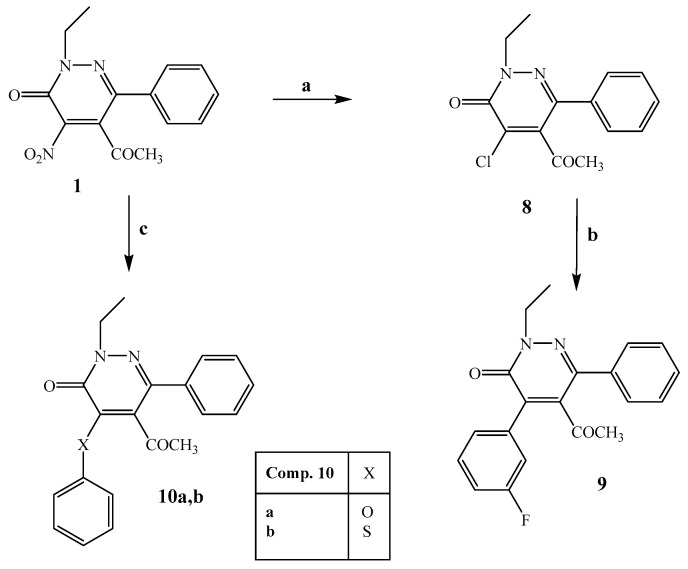
Modifications performed at position 4 of the pyridazinone scaffold. Synthesis of final compounds **9** and **10a**,**b**. Reagent and conditions: (a) 6N HCl, acetone, 100 °C, 5 h. (b) Tetrakis(triphenylphosphine)palladium(0), 3-F-phenylboronic acid, 2M Na_2_CO_3_, anhydrous toluene, under N_2_, 100 °C, 10 h. (c) Sodium phenoxyde or sodium thiophenolate, anhydrous ethanol, r.t., 2 h.

The synthesis of all final compounds (previously patented [33,34,35]) is depicted in Scheme 1, Scheme 2 and Scheme 3, and the structures are confirmed based on analytical and spectral data. In Scheme 1 and Scheme 2 are reported the synthetic pathways affording the final products of types **2**, **3**, **4,** and **5,** in which we introduced an amino group at position 4 of pyridazinone [33,34,35]. All compounds reported in Scheme 1 (see Table 1, Table 2, Table 3 and Table 4) were obtained starting from the common key intermediate 5-acetyl-2-ethyl-4-nitro-6-phenylpyridazin-3(2H)-one **1** [30], whose nitro group at position 4 proved to be an excellent leaving group easily replaced by a variety of nucleophiles [31]. Therefore, the treatment of **1** with the appropriate (cyclo)alkyl, aryl, or (hetero)aryl/alkylamine in ethanol at room temperature afforded the desired final compounds of types **2**–**5** in good yields. The only exceptions are compounds **2o**, **3g** and **5d**; for **2o** and **5d,** the reaction mixture was heated at 60 °C for 1 h and 90 °C for 3 h, respectively, while for compound **3g**, the reaction was conducted in a microwave oven for 2 h. The final **3f** was further transformed into **3j** by treatment with 3-chloroperoxybenzoic acid in dichloromethane at room temperature for 27 h. For the synthesis of the final compounds **2t**–**w** [33] and **3k**,**l** [33,34] (Scheme 2), the key intermediate is represented by 4-aminopyridazinone **7** obtained starting from isoxazolo [3,4-d]pyridazinone **6**, previously described by us [32], by reductive cleavage with ammonium formate and 10% Pd/C in ethanol. Intermediate **7** was treated with the appropriate arylboronic acid in the presence of anhydrous cupric acetate and triethylamine in anhydrous methylene chloride, affording the desired compounds **2t**–**w** and **3l**. Instead, by reacting compound **7** with 3-bromo-4-methylpyridine in the presence of anhydrous cuprous iodide and potassium carbonate at 145 °C for 12 h, compound **3k** was recovered.

Finally, in Scheme 3, the synthesis of compounds **9** and **10a**,**b** is reported, lacking the amino group at position 4 of pyridazinone, deleted in compound **9** or replaced with O or S in compounds **10a**,**b**. For treatment of key intermediate **1** with hydrochloric acid in acetone at high temperature, the 4-Cl derivative **8** was obtained and then converted into the final **9** by Suzuki coupling reaction with tetrakis(triphenylphosphine)palladium(0) and 3-fluorophenilboronic acid and into compounds **10a**,**b** by nucleophilic displacement of the nitro group of compound **1** with appropriate commercially available reagent (sodium phenoxide or sodium thiophenolate respectively).

### 2.2. Pharmacology/Biology

#### 2.2.1. PDE4 Inhibitory Activity and SAR Analysis

All newly synthesized compounds were first evaluated in vitro for their ability to inhibit PDE4B1, the most expressed subtype in inflammatory and immune cells, by measuring the inhibition of hydrolysis of radiolabeled [^3^H]cAMP. Compounds with IC_50_ ≤ 100 nM on PDE4B1 were also tested on subtypes PDE4A4 and PDE4D3, and for their ability to displace [^3^H]Rolipram from its binding site (HARBS) as well as to inhibit TNFα production in human whole blood (hWB). Roflumilast and Rolipram are the reference compounds (Table 5, Table 6, Table 7 and Table 8). Finally, two selected products (**3a** and **3k**) have been extensively and thoroughly investigated with further in vitro tests on metabolism and in vivo studies on different animal models (rat, ferret, and dog). Starting from 4-(substituted)-aniline derivatives of type 2 (Table 5), most of the newly synthesized compounds exhibited very potent PDE4 inhibitory activity, with IC_50_ values in the nanomolar or sub-nanomolar range, and resulted in a good decrease of TNFα production. To allow easier and more direct reading of the results, the compounds in Table 5 are discussed based on the position of the aniline bearing the substituent (*o*, *m*, *p*). The first example is the 4-aniline derivative **2a**, which showed IC_50_ = 29.01 nM, 4.7 nM, and 5.23 nM for PDE4B1, PDE4A4, and PDE4D3, respectively, values that correlate well with the effect on TNFα production (IC_50_ = 38.22 nM). The introduction at the meta position of **2a** of various groups/atoms appears favorable for activity regardless of their electronic features and size. In fact, groups/atoms such as F (**2b**), Cl (**2c**), CN (**2f**) and NO_2_ (**2t**), led to an increase of activity on PDE4B1 with respect to **2a** with IC_50_ = 0.73–8.91 nM, whereas on the other subtypes PDE4A4 and PDE4D3, HARBS and TNFα production, the potency remains about the same. Also, the insertion of Br (**2d**) or OCH_3_ (**2e**) results in active compounds, with IC_50_ values similar to those of the unsubstituted compound **2a**, but with lower activity on TNFα production (130.02–230.01 nM vs. 38.22 nM). The most interesting in the series of meta-substituted compounds is **2f** (R = CN) which, in addition to being the most active on PDE4B1 (IC_50_ = 0.73 nM), shows a decrease of affinity for PDE4D3 (IC_50_ = 17.0 nM), PDE4A4 (IC_50_ = 40.01 nM) and especially for HARBS (IC_50_ = 190.02 nM), but strongly inhibits TNFα production (IC_50_ = 3.7 nM). Instead, the introduction in the same position of groups such as CF_3_, COOH, and OH led to a decrease of activity (**2g**–**i** IC_50_ = 229.21 nM, 2998.01 nM and 907.02 nM for PDE4B1, respectively).

Interestingly, when fluorine, chlorine and the methoxy group are moved to the ortho position (compounds **2j**, **2u** and **2v** as 2-isomer of **2b**, **2c** and **2e** above discussed) the IC_50_ values on the three isotypes and on HARBS remain almost the same, but their ability to inhibit TNFα production is greatly diminished (IC_50_ = 95.03–480.00 nM, respect to IC_50_ = 14.32–230.00 nM of **2b**, **2c**, **2e**). Moving to compounds substituted in the para-position of the phenyl ring, we can observe a general decrease in activity (one/two orders of magnitude) with respect to the corresponding meta-substituted compounds (**2k**–**m** versus **2c**–**e** and **2q** versus **2f**). The 4-CN-derivative **2q**, despite being less potent than its isomer with the ciano group at position 3 **2f** (IC_50_ = 20.06 nM and 0.73 nM, respectively), still shows less affinity for subtype PDE4D3 and HARBS and maintains a good level of control on TNFα production (IC_50_ = 61.03 nM). Instead, the shift of the nitro group from meta (**2t**) to para position (**2p**) affords a compound about fourfold more potent for PDE4B1 than the corresponding 3-isomer **2t** (IC_50_ = 1.41 nM versus 7.20 nM), with a lower affinity for HARBS, but also for TNFα (IC_50_ = 239.23 nM). Likewise, when COOH is moved from position 3 (compound **2i**) to 4 position (compound **2n** [36]) of the phenyl, a considerable increase of activity for PDE4B1 isotype is observed (IC_50_ = 9.24 nM) but also an unwanted good affinity for subtype PDE4D3 and for HARBS (IC_50_ = 6.02−52.04 nM respectively) while the ability to inhibit TNFα production is poor (IC_50_ = 320.11 nM). The esterification of **2n** (compound **2o**) led to a loss of activity (IC_50_ = 6700 nM), thus suggesting the importance of a carboxyl group in this position. Finally, the double substitution on the aniline ring is compatible for activity only with the insertion of two fluorine atoms at positions 3 and 5 (compound **2w**); this modification affords a very potent compound endowed with nanomolar activity for all PDE4 subtypes together with a very good inhibition of TNFα production (IC_50_ = 17.01 nM) but at the same time displays a strong affinity for HARBS (IC_50_ = 16.24 nM). On the contrary, the introduction of substituents in meta and para positions, as in compounds **2r** and **2s,** is detrimental to activity. In Table 6 the biological data concerning the aryl- or hetero-arylamino derivatives of type 3 are reported, in which the phenyl of the aniline group at position 4 has been replaced by heterocycles, as pyridine and quinoline, or by a variously substituted naphthalene nucleus. Similar to Table 5, for a clearer presentation, the products are grouped and discussed according to the (hetero)aromatic linked to the amine group at position 4 (pyridine, quinoline and naphthalene). These compounds showed an interesting inhibitory activity on PDE4 isoforms sometimes also exhibiting a low affinity for HARBS. Compounds bearing at amino group of position 4 a pyridine (compounds **3a** and **3k**) or a quinoline variously substituted (compounds **3b**, **3f**, **3h**–**j**) showed an interesting PDE4 inhibitory activity in the low nanomolar range (IC_50_ = 0.36–26.45 nM) for all analyzed subtypes, and an appreciable TNFα inhibition (IC_50_ = 2.40–410.11 nM), but most of them also exhibited a very good affinity for HARBS (IC_50_ = 5.10–150.02 nM). For example, the 4-quinoline-5-ylamino derivative **3f** endowed with a subnanomolar potency toward PDE4B1 (IC_50_ = 0.36 nM), associated with a good selectivity (two orders of magnitude) for the other two isotypes and overall, with an excellent inhibitory activity on TNFα production (IC_50_ = 2.40 nM), showed a very high affinity for HARBS (IC_50_ = 5.10 nM). Also, its N-oxide derivative **3j** maintained a similar profile, with the potency on TNFα production (IC_50_ = 5.97 nM) still being associated with high affinity for HARBS.

**Figure 4:**
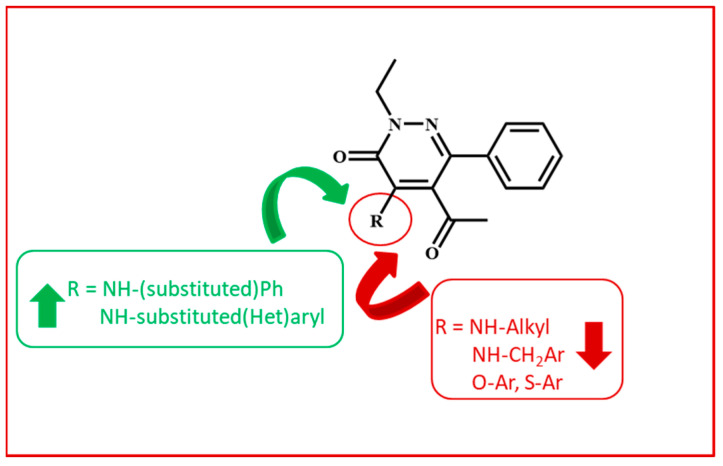
SAR of new synthesized compounds.

Finally, the naphtylamino derivatives **3l**, **3c**-**e** and **3g** showed a comparable profile for activity and selectivity to that of the quinoline analogues. In this series, compound **3g**, bearing a NO_2_ group in position 4 of naphthalene ring was found to be the most interesting for activity (IC_50_ = 0.73 nM for PDE4B1) and selectivity, especially concerning HARBS (IC_50_ = 120.32 nM), but it exhibited low potency in TNFα production (IC_50_ = 220.0 nM). Moving on to those compounds showing a (cyclo)alkyl at NH of position 4 in the pyridazinone scaffold (**4a**-**g** and **7**, Table 7) or a linker between the NH group and the (hetero) aromatic ring (compounds **5a**-**l**, Table 8), it is immediately clear a marked decrease of the activity with IC_50_ for PDE4B1in the (sub)micromolar range, and the only compound endowed with a certain activity is the pyridine derivative **5i** (IC_50_ = 82.51 nM). Also, compounds **9** and **10a**,**b** (Table 8), in which the NH group has been eliminated (compound **9**) or replaced with S or O (compounds **10a**,**b,** respectively), exhibited very low (**10a**) or no (**9**, **10b**) inhibitory activity on PDE4B1. The results shown in Table 7 and Table 8 provide very important indications regarding the importance of the group linked to position 4 of the pyridazinone scaffold. In fact, as the elimination of the (hetero)aromatic ring linked at 4-NH (compound **7**) as its replacement with a (cyclo)alkyl (**4a**–**g**) (Table 7) afforded low active compounds, thus suggesting the importance of a bulky aromatic system and possible π–π interactions with the enzyme. On the other hand, it appears that the NH and the (hetero)aromatic group must be directly linked, as shown by the low activity values of those compounds in which a -CH_2_- has been inserted between the NH and the (hetero)aromatic group (ie **5d** versus **2n**, IC_50_ = 5200 and 9.24 nM respectively; **5g** versus **3a**, IC_50_ = 113.04 and 19.54 nM respectively), and even more so when an ethylene linker (**5c** and **5j**) is inserted (ie **5h** versus **5j**, IC_50_ = 181.23 and 1200.03 nM respectively), (Table 8). Finally, the importance of the presence of a secondary amine (NH) at position 4 of the scaffold, is highlighted by the low activity of compounds **5f** (Table 8), bearing a tertiary amine, and of compound **7** (Table 7) bearing a primary amine (NH_2_), and confirmed by the inactivity of compounds **9** and **10a,b** (Table 8), avoid of NH group. Analyzing all these data and overall, those of the derivatives of type 2, it is not easy to obtain understandable structure-activity relationships (Figure 4). For the series of anilino derivatives (compounds types 2) in fact, we can observe that in meta position of aniline group the presence of a small electron-withdrawing group, such as an F or a CN group, is favored, while the presence of polar groups (OH or COOH) is not tolerated. On the contrary, in the para position, the best results are obtained with a COOH or a NO_2_ groups, while small atoms electron-withdrawing cause a collapse of activity. Finally, in the ortho position, substituents with very different electronic and steric characteristics seem to be tolerated. Thus, to try to rationalize the biological results and to find a discriminant among the active and low active ligands, we decided to carry out molecular modeling studies on some of these compounds, choosing among the various types of derivatives 2–5 (see below).

#### 2.2.2. In Vitro and In Vivo Profile of Compounds **3a** and **3k**

Some of the most potent and interesting compounds have been subjected to metabolism studies, and it was observed that 4-anilino (compounds of type **2**, Table 5) and 4-quinolinylamino derivatives (compounds of type **3**, Table 6), but not pyridylamino derivatives **3a** and **3k**, generated reactive compounds that gave GSH adducts under the experimental conditions. Therefore, compounds **3a** and **3k** were selected for an in-depth in vitro and in vivo studies to evaluate their efficacy, their pharmacokinetic and toxicity profile after oral administration as well as their ability to induce emesis in ferrets (in these experiments the drug Roflumilast is the reference compound). To provide an overview of the profile of the selected compounds **3a** and **3k**, in Table 9, the data of new in vitro studies are reported, TNFα hPBMC and CaCo-2, together with the inhibition values on PDE4 isotypes and on TNFα hWB, already discussed in Table 6. It can be seen that as compounds **3a** and **3k** exhibit very similar values to Roflumilast on both TNFα hWB and TNFα hPBMC, in particular compound **3k**. Furthermore, compounds **3a** and **3k** show a favorable permeability (CaCo-2 assay) comparable to Roflumilast, exhibiting IC_50_ = 39 nM, 45 nM respectively versus IC_50_ = 39 nM of Roflumilast.

As regards in vivo studies, compared to the reference drug, compounds **3a** and **3k** are instead less active in the neutrophilia rat lipopolysaccharide (LPS) in vivo models, measuring the inhibition of the increase of neutrophils in bronchoalveolar lavage (BAL) fluid. Compounds **3a** and **3k** show a favorable absorption profile for oral administration and acceptable metabolic stability, comparable to Roflumilast, but with shorter half-life and lower oral bioavailability due to higher clearance (Table 10).

Interestingly, both analyzed compounds after oral administration in ferrets show a lower emetic effect compared to Roflumilast, exhibiting a Maximal Not Emetic Dose (MNED mg/kg) 3–10-fold higher than that of Roflumilast (in ferret MNED = 2.7, 1.1, and 0.3 mg/kg for **3a**, **3k,** and Roflumilast, respectively, Table 10).

Finally, compound **3k** shows a more favorable toxicological profile with respect to other PDE4 inhibitors (i.e., Rolipram, Roflumilast, and Cilomilast), since no adverse or toxicological events have been observed after 2 weeks of administration in rats (up to 10 mg/kg/day).

### 2.3. Molecular Modeling

A molecular docking by AUTODOCK [37] and a potential energy minimization of the ligand-enzyme complexes was carried out on eight selected active ligands (**Roflumilast**, **2c**, **2e**, **2f, 2n**, **2p**, **3a**, **3k**, 0.69 nM < IC_50_ < 19.54, Class Active, A) and eight very low active ligands (**Rolipram**, **2i**, **2h**, **2o**, **4f**, **5d**, **7**, **10a**, 520 nM < IC_50_ < 6700 nM, Class Inactive, I) on PDE4B1. All 3D structures of the molecules were designed [38] carboxylic group was considered charged (carboxylate group COO-) in compounds **2i**, **2n,** and **5d**.

The enzyme 3GWT (resolution 1.75Å) co-crystallized with a quinolone compound [39,40] was chosen for our docking and minimization studies. In this study, hydrogen bond lengths were measured after complete relaxation of the enzyme-ligand complex, and we considered the poses calculated by AutoDock acceptable. Docking studies were performed considering the entire enzyme structure in vacuum and with water molecules present at the catalytic site (i.e., 14 water molecules).

AUTODOCK furnished 100 poses of the ligand-enzyme complex, which were grouped into clusters within 5 RMSD; these clusters were considered the clusters at the lowest energy and covering at least 70% of all poses. Table S1 (see Supplementary Materials) reports the number of poses for each ligand-enzyme in vacuum and with 14 water molecules.

The potential energy minimization of the complexes ligand-enzyme using GROMACS [41] with forcefield AMBER99sb (details in Supplementary Materials) permitted survey of the hydrogen bond between ligand amino acid residues and water (when present) of the catalytic site, with a cutoff of 3.5Å, that is, the hydrogen bond is collected if the distance between hydrogen and hydrogen acceptor (A) is lower than 3.5Å with angles included between 120° and 170° (ligand-D-H…..A-enzyme and ligand-A….H-D-enzyme).

From minimization data, we extract two parameters: the mean of hydrogen bond distance (Å) between the hydrogen and hydrogen bond acceptor (A) and the number of hydrogen bonds/conformation (pose), in vacuum and with water molecules (fourteen water molecules), considering in the two situations, the interaction with amino acids or with amino acids and water.

In vacuum, all the ligands (class A and class I) interact with amino acid residues showing about the same average number of hydrogen bonds/pose (3.8 vs. 3.7, respectively). In the presence of water, this average is slightly higher for the active ligands (4.59 vs. 3.5, respectively). Non-significant difference (*p* > 0.05) between the average of the distance between H-acceptor for actives and low-actives ligand in vacuum (2.84 vs. 2.75) and with water molecules (2.89 vs. 2.87) (see Table S2, Supplementary Materials).

When we consider the involvement of water molecules in the compounds’ interactions in the enzyme active site, we observe a statistically significant difference (*p* < 0.007) between active (class A) and low-active (class I) ligands (Table S3, Supplementary Materials). The less active compounds (Class I) form hydrogen bonds with water on average shorter and, therefore, stronger with respect to class A ligands (2.18 Å vs. 2.64 Å). The average distance (Å) of hydrogen bonds between active and low-active ligands with enzyme water molecules (fourteen) is significantly different (*p* < 0.05). This behaviour agrees with what has already been evidenced and explained in our previous work [42].

Moreover, less active compounds (Class I) show a more significant number of hydrogen bonds than active compounds (2.13 vs. 1.47), another possible explanation for the difference in activity.

**Figure 5:**
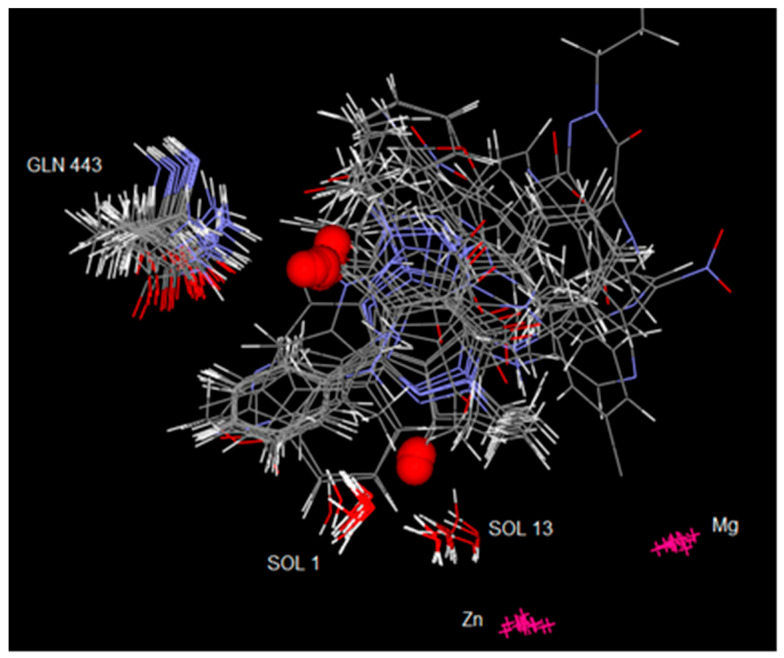
Active ligands: H-bond between COCH_3_ (red spheres) with water molecules (SOL13) e con il Gln443. The number of H-bonds with GLN443 is greater than the number of H-bonds with water.

**Figure 6:**
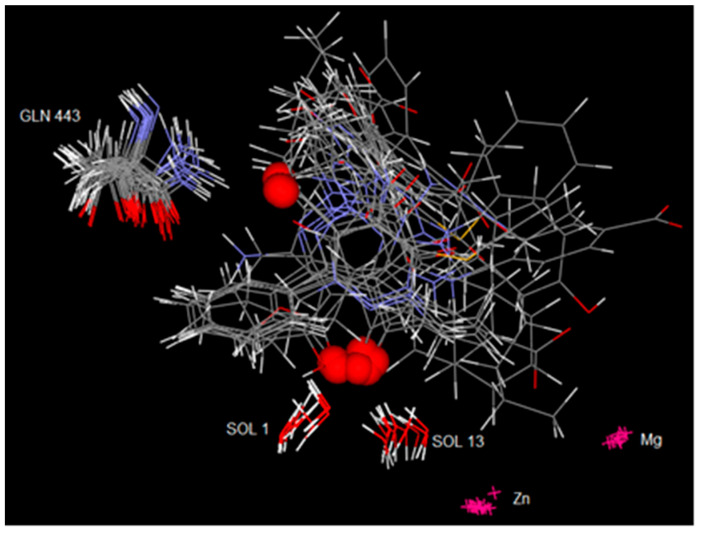
Low active ligands: H-bond between COCH_3_ (red spheres) with water molecules (SOL1 e SOL13 e, and con il Gln443). The number of H-bonds with water is greater than the number of H-bonds with GLN443.

The presence of the -COCH_3_ group at position 5 in active and low-active compounds does not seem to be a discriminator, but if we consider the involvement of this group in the hydrogen bond interactions, we can see a substantial difference. In the minimized complexes enzyme-ligand, the principal hydrogens interactions (distance DH…A < 3.5 Å; angle N-H….O e O-H…. O in the range of 120° and 170°) evidenced between the oxygen of COCH_3_, water molecules (SOL1 and SOL13, near to Zn and Mg ions) and Gln443, are different in active and low active compounds (Table 11 and Table 12 and Figure 5 and Figure 6). In particular, for the low active molecules, a high number of hydrogen bonds between the COCH_3_ oxygen and water molecules (SOL1 and SOL13) happen (1.4 bounds/pose, average distance 2.47 Å), with respect to active ligands (Group A, 0.8 bounds/pose, average distance 2.47 Å). Regarding the interaction with the residue Gln443, the situation is the opposite: the active molecules have a significant number of hydrogen interactions between NH of the residue and COCH_3_ oxygen (0.8 bounds/pose, average distance 2.77 Å) while the low active molecules show a lower number of bounds/pose (0.6) with a greater distance (average 2.81 Å).

Table 11 and Table 12 report the distance between C**O**CH_3_ and **HN**-Gln443 or water molecules (SOL1 and SOL13) for each compound pose for active and low-active molecules.

Figure 5 and Figure 6 represent the minimized complexes, enzyme-active compounds, and enzyme-low-active compounds; the red spheres represent the oxygen atoms of the COCH_3_ group involved in hydrogen bonding. It is possible to note, as previously mentioned, the different numbers of hydrogen bonds between water and Gln443 of the active and low-active ligands.

In the minimised complex roflumilast-enzyme, the strong hydrogen bond interaction (2.02 Å) between the oxygen (of cyclopropylmethyloxy group) and Gln443 is evidenced (see Figure S1 in Supplementary Materials). Moreover, from the overlap of compounds Roflumilast, **3a**, and **3k**, it is possible to see that these latter compounds interact through the carbonyl group of COCH_3_ with solvent molecules (see Figure S2 in Supplementary Materials).

## 3. Materials and Methods

### 3.1. Chemistry

#### 3.1.1. Physical Measurements and Materials

Reagents and starting materials were obtained from commercial sources. Extracts were dried over Na_2_SO_4_, and the solvents were removed under reduced pressure. All reactions were monitored by thin-layer chromatography (TLC) using commercial plates precoated with Merck silica gel 60 F-254 (Merck, Milano Italy). Visualization was performed by UV fluorescence (λmax = 254 nm) or by staining with iodine or potassium permanganate. Chromatographic separations were performed on a silica gel column by gravity chromatography (Kieselgel 40, 0.063–0.200 mm; Merck), flash chromatography (Kieselgel 40, 0.040–0.063 mm; Merck. Yields refer to chromatographically and spectroscopically pure compounds, unless otherwise stated. Compounds were named following IUPAC rules, as applied by Beilstein-Institut AutoNom 2000 (4.01.305) or CA Index Name. All melting points were determined on a microscope hot stage Büchi apparatus and are uncorrected. The identity and purity of intermediates and final compounds were ascertained through TLC chromatography and NMR. ^1^H-NMR and ^13^C NMR spectra were recorded with Avance 400 instruments (Bruker Biospin Version 002 with SGU). Chemical shifts (δ) are reported in ppm to the nearest 0.01 ppm using solvent as the internal standard. Coupling constants (J values) are given in Hz and were calculated using ‘TopSpin 1.3’ software, rounded to the nearest 0.1 Hz. Mass spectra (*m*/*z*) were recorded on a Micromass ZMD mass spectrometer using ESI ionization. All new compounds had a purity ≥ 95%; microanalyses indicated by the symbols of the elements or functions were performed with a Perkin-Elmer 260 elemental analyzer for C, H, and N, and they were within ±0.4% of the theoretical values.

#### 3.1.2. General Procedure for the Synthesis of Compounds **2a**–**s**

To a stirred solution of 5-acetyl-2-ethyl-4-nitro-6-phenylpyridazin-3(2H)-one **1** [30] (0.30 mmol) in 1–2 mL of ethanol, appropriate arylamine (0.30–0.90 mmol) was added portion-wise. The resulting mixture was stirred at room temperature for 30 min–3h (65 h for compound **2p** and 20 h for compound **2q**). For compound **2o** the mixture was heated at 60 °C for 1 h. After cooling the final products were collected by filtration and washed with ethanol.

##### *5-Acetyl-2-ethyl-6-phenyl-4-phenylaminopyridazin-3(2H)-one,* **2a**

Yield = 43%; mp = 187–189 °C (EtOH); ^1^H-NMR (400 MHz, CDCl_3_) δ 1.40 (t, 3H, CH_2_*CH_3_*, J = 7.2 Hz), 1.70 (s, 3H, COCH_3_), 4.30 (q, 2H, CH_2_*CH_3_*, J = 7.2 Hz), 7.20 (m, 10H, Ar), 8.21(exch br s, 1H, NH). ^13^C-NMR (100 MHz, CDCl_3_) δ 12.21, 29.64, 37.10, 107.32, 120.80, 122.41, 128.81, 129.26, 129.52, 131.04, 137.91, 142.28, 146.32, 152.71, 162.1, 200.1. ESI-MS calcd. for C_20_H_19_N_3_O_2_, 333.38; found: *m*/*z* 334.16 [M+H]^+^.

##### *5-Acetyl-2-ethyl-4-(3-fluorophenylamino)-6-phenylpyridazin-3(2H)-one,* **2b**

Yield = 52%; mp = 174–175 °C (EtOH); ^1^H-NMR (400 MHz, CDCl_3_) δ 1.45 (t, 3H, CH_2_*CH_3_*, J = 7.2 Hz), 1.75 (s, 3H, COCH_3_), 4.30 (q, 2H, *CH_2_*CH_3_, J = 7.2 Hz), 6.80 (m, 3H, Ar), 7.30 (m, 6H, Ar), 8.20 (exch br s, 1H, NH). ^13^C-NMR (100 MHz, CDCl_3_) δ 12.15, 29.43, 36.92, 107.51, 110.50, 111.22, 111.90, 128.85, 129.43, 131.22, 131.46, 138.11, 146.06, 146.33, 152.92, 162.38, 163.71, 200.10. ESI-MS calcd. for C_20_H_18_FN_3_O_2_, 351.37; found: *m*/*z* 352.12 [M+H]^+^.

##### *5-Acetyl-4-(3-chlorophenylamino)-2-ethyl-6-phenylpyridazin-3(2H)-one,* **2c**

Yield = 64%; mp = 186–187 °C (EtOH); ^1^H-NMR (400 MHz, CDCl_3_) δ 1.50 (t, 3H, CH_2_*CH_3_*, J = 7.2 Hz), 1.80 (s, 3H, COCH_3_), 4.30 (q, 2H, *CH_2_*CH_3_, J = 7.2 Hz), 7.20 (m, 4H, Ar), 7.40 (s, 5H, Ar), 8.20 (exch br s, 1H, NH). ^13^C-NMR (100 MHz, CDCl_3_) δ 12.22, 29.51, 37.26, 107.48, 114.61, 116.59, 122.33, 128.90, 129.24, 130.81, 131.13, 135.14, 137.82, 146.30, 152.71, 162.14, 200.21. ESI-MS calcd. for C_20_H_18_ClN_3_O_2_, 367.83; found: *m*/*z* 368.59 [M+H]^+^.

##### *5-Acetyl-4-(3-bromophenylamino)-2-ethyl-6-phenylpyridazin-3(2H)-one,* **2d**

Yield = 65%; mp = 189–190 °C (EtOH); ^1^H-NMR (400 MHz, CDCl_3_) δ 1.50 (t, 3H, CH_2_CH_3_, J = 7.2 Hz), 1.80 (s, 3H, COCH_3_), 4.30 (q, 2H, CH_2_CH_3_, J = 7.2 Hz), 7.20 (m, 4H, Ar), 7.40 (m, 5H, Ar), 8.20 (exch br s, 1H, NH). ^13^C-NMR (100 MHz, CDCl_3_) δ 12.0,3 29.62, 37.08, 107.32, 115.34, 115.55, 121.67, 123.95, 128.81, 129.10, 130.62, 131.31, 137.92, 146.62, 152.65, 162.30, 200.13 ESI-MS calcd. for C_20_H_18_BrN_3_O_2_, 412.28; found: *m*/*z* 412.98 [M+H]^+^.

##### *5-Acetyl-2-ethyl-4-(3-methoxyphenylamino)-6-phenylpyridazin-3(2H)-one,* **2e**

Yield = 54%; mp = 158–159 °C (EtOH); ^1^H-NMR (400 MHz, DMSO-*d_6_*) δ 1.33 (t, 3H, CH_2_*CH_3_*, J = 7.2 Hz), 1.71 (s, 3H, COCH_3_), 3.68 (s, 3H, OCH_3_), 4.16 (q, 2H, *CH_2_*CH_3_, J = 7.2 Hz), 6.65 (m, 3H, Ar), 7.15 (m, 1H, Ar), 7.32 (m, 5H, Ar), 8.92 (exch br s, 1H, NH). ^13^C-NMR (100 MHz, CDCl_3_) δ 12.04, 29.72, 36.92, 55.83, 104.90, 107.31, 108.62, 110.84, 128.90, 129.06, 130.54, 131.06, 138.18, 145.49, 146.31, 152.76, 161.41, 161.97, 200.21. ESI-MS calcd. for C_21_H_21_N_3_O_3_, 363.41; found: *m*/*z* 364.18 [M+H]^+^.

##### *3-(5-Acetyl-2-ethyl-3-oxo-6-phenyl-2,3-dihydropyridazin-4-ylamino)-benzonitrile,* **2f**

Yield = 53%; mp = 226–227 °C (EtOH); ^1^H-NMR (400 MHz, CDCl_3_) δ 1.44 (t, 3H, CH_2_*CH_3_*, J = 7.3 Hz), 1.78 (s, 3H, COCH_3_), 4.30 (q, 2H, *CH_2_*CH_3_, J = 7.1 Hz), 7.40 (m, 9H, Ar), 8.64 (exch br s, 1H, NH). ^13^C-NMR (100 MHz, CDCl_3_) δ 12.24, 29.61, 37.15, 107.38, 113.42, 118.60, 120.63, 126.52, 127.81, 128.83, 129.28, 130.22, 131.04, 145.14, 146.32, 152.76, 162.11, 200.16. ESI-MS calcd. for C_21_H_18_N_4_O_2_, 358.39; found: *m*/*z* 359.18 [M+H]^+^.

##### *5-Acetyl-2-ethyl-6-phenyl-4-(3-trifluoromethylphenylamino)pyridazin-3(2H)-one,* **2g**

Yield = 35%; mp = 157–159 °C (EtOH); ^1^H-NMR (400 MHz, CDCl_3_) δ 1.50 (t, 3H, CH_2_*CH_3_*, J = 7.2 Hz), 1.70 (s, 3H, COCH_3_), 4.30 (q, 2H, *CH_2_*CH_3_, J = 7.2 Hz), 7.40 (m, 9H, Ar), 8.40 (exch br s, 1H, NH). ^13^C-NMR (100 MHz, CDCl3) δ 12.32, 29.64, 37.38, 107.20, 115.15, 119.67, 120.24, 124.28, 128.82, 129.01, 129.74, 131.06, 131.72, 137.81, 138.57, 146.21, 152.79, 161.90, 200.14. ESI-MS calcd. for C_21_H_18_F_3_N_3_O_2_, 401.38; found: *m*/*z* 402.14 [M+H]^+^.

##### *5-Acetyl-2-ethyl-4-(3-hydroxyphenylamino)-6-phenylpyridazin-3(2H)-one,* **2h**

Yield = 22%; mp = 185–187 °C (EtOH); ^1^H-NMR (400 MHz, CDCl_3_) δ 1.50 (t, 3H, CH_2_*CH_3_*, J = 7.2 Hz), 1.80 (s, 3H, COCH_3_), 4.30 (q, 2H, *CH_2_*CH_3_, J = 7.2 Hz), 6.60 (m, 3H, Ar), 7.40 (m, 6H, Ar), 8.10 (exch br s, 1H, NH). ^13^C-NMR (100 MHz, CDCl_3_) δ 12.13, 29.64, 37.28, 100.16, 107.81, 108.90, 109.23, 128.71, 129.23, 130.82, 131.10, 137.76, 145.82, 146.19, 152.54, 159.16, 161.90, 200.04. ESI-MS calcd. for C_20_H_19_N_3_O_3_, 349.38; found: *m*/*z* 350.19 [M+H]^+^.

##### *3-(5-Acetyl-2-ethyl-3-oxo-6-phenyl-2,3-dihydropyridazin-4-ylamino)benzoic Acid,* **2i**

Yield = 77%; mp = 262–264 °C (EtOH); ^1^H-NMR (400 MHz, CDCl_3_) δ 1.40 (t, 3H, CH_2_*CH_3_*, J = 7.2 Hz), 1.80 (s, 3H, COCH_3_), 4.40 (q, 2H, *CH_2_*CH_3_, J = 7.2 Hz), 7.30 (m, 6H, Ar), 7.80 (s, 1H, Ar), 7.92 (m, 2H, Ar), 8.43 (exch br s, 1H, NH). ^13^C-NMR (100 MHz, CDCl_3_) δ 12.25, 29.82, 37.16, 107.34, 115.77, 120.33, 121.65, 128.82, 128.91, 129.27, 129.44, 131.02, 137.91, 146.43, 152.76, 166.37, 200.10. ESI-MS calcd. for C_21_H_19_N_3_O_4_, 377.79; found: *m*/*z* 378.54 [M+H]^+^.

##### *5-Acetyl-2-ethyl-4-(2-fluorophenylamino)-6-phenylpyridazin-3(2H)-one,* **2j**

Yield = 43%; mp = 184–186 °C (EtOH); ^1^H-NMR (400 MHz, DMSO-*d_6_*) δ 1.32 (t, 3H, CH_2_*CH_3_*, J = 7.2 Hz), 1.58 (s, 3H, COCH_3_), 4.16 (q, 2H, *CH_2_*CH_3_, J = 7.2 Hz), 7.15 (m, 6H, Ar), 7.35 (m, 3H, Ar), 8.85 (exch br s, 1H, NH). ^13^C-NMR (100 MHz, CDCl_3_) δ 12.26, 29.52, 36.91, 107.32, 116.23, 118.01, 123.44, 127.81, 128.86, 129.23, 130.44, 131.21, 137.74, 146.33, 152.62, 153.23, 162.10, 200.23. ESI-MS calcd. for C_20_H_18_FN_3_O_2_, 351.37; found: *m*/*z* 352.16 [M+H]^+^.

##### *5-Acetyl-4-(4-chlorophenylamino)-2-ethyl-6-phenylpyridazin-3(2H)-one,* **2k**

Yield = 64%; mp = 183–184 °C (EtOH); ^1^H-NMR (400 MHz, CDCl_3_) δ 1.51 (t, 3H, CH_2_*CH_3_*, J = 7.2 Hz), 1.80 (s, 3H, COCH_3_), 4.29 (q, 2H, *CH_2_*CH_3_, J = 7.2 Hz), 7.00 (d, 2H, Ar, J = 8.4 Hz), 7.35 (d, 2H, Ar, J = 8.3 Hz), 7.40 (s, 5H, Ar), 8.00 (exch br s, 1H, NH). ^13^C-NMR (100 MHz, CDCl_3_) δ 12.25, 29.62, 37.01, 107.56, 120.97, 127.53, 128.68, 129.22, 129.51, 131.26, 138.01, 140.37, 146.13, 152.75, 162.06, 200.21. ESI-MS calcd. for C_20_H_18_ClN_3_O_2_, 367.83; found: *m*/*z* 368.54 [M+H]^+^.

##### *5-Acetyl-4-(4-bromophenylamino)-2-ethyl-6-phenylpyridazin-3(2H)-one,* **2l**

Yield = 70%; mp = 185–186 °C (EtOH); ^1^H-NMR (400 MHz, CDCl_3_) δ 1.30 (t, 3H, CH_2_*CH_3_*, J = 7.2 Hz), 1.80 (s, 3H, COCH_3_), 4.30 (q, 2H, *CH_2_*CH_3_, J = 7.2 Hz), 6.90 (d, 2H, Ar, J = 8.3 Hz), 7.30 (d, 2H, Ar, J = 8.3 Hz), 7.40 (s, 5H, Ar), 8.23 (exch br s, 1H, NH). ^13^C-NMR (100 MHz, CDCl_3_) δ 12.29, 29.47, 37.38, 107.33, 116.61, 116.93, 128.84, 129.12, 131.21, 132.45, 137.90, 141.05, 146.33, 152.72, 162.14, 200.12. ESI-MS calcd. for C_20_H_18_BrN_3_O_2_, 412.28; found: *m*/*z* 413.01 [M+H]^+^.

##### *5-Acetyl-2-ethyl-4-(4-methoxyphenylamino)-6-phenylpyridazin-3(2H)-one,* **2m**

Yield = 67%; mp = 161–162 °C (EtOH); ^1^H-NMR (400 MHz, CDCl_3_) δ 1.45 (t, 3H, CH_2_*CH_3_*, J = 7.2 Hz), 1.70 (s, 3H, COCH_3_), 3.80 (s, 3H, OCH_3_), 4.30 (q, 2H, *CH_2_*CH_3_, J = 7.2 Hz), 6.80 (d, 2H, Ar, J = 8.4 Hz), 7.05 (d, 2H, Ar, J = 8.3 Hz), 7.30 (m, 5H, Ar), 8.02 (exch br s, 1H, NH). ^13^C-NMR (100 MHz, CDCl_3_) δ 12.21, 29.68, 37.04, 55.84, 107.43, 115.02, 117.31, 128.81, 129.25, 131.03, 134.57, 138.03, 146.21, 152.78, 153.63, 162.41, 200.13. ESI-MS calcd. for C_21_H_21_N_3_O_3_, 363.41; found: *m*/*z* 364.19 [M+H]^+^.

##### *4-(5-Acetyl-2-ethyl-3-oxo-6-phenyl-2,3-dihydropyridazin-4-ylamino)benzoic Acid,* **2n** [36]

Yield = 77%; mp = 252 °C dec. (EtOH); ^1^H-NMR (400 MHz, DMSO-*d_6_*) δ 1.37 (t, 3H, CH_2_*CH_3_*, J = 7.2 Hz), 1.80 (s, 3H, COCH_3_), 4.20 (q, 2H, *CH_2_*CH_3_, J = 7.2 Hz), 7.03 (d, 2H, Ar, J = 8.6 Hz), 7.33 (m, 3H, 2H Ar + 1H OH), 7.58 (d, 2H, Ar, J = 8.3 Hz), 7.78 (d, 2H, Ar, J = 8.3 Hz), 8.01 (m, 2H, Ar), 9.18 (exch br s, 1H, NH). ^13^C-NMR (100 MHz, CDCl_3_) δ 12.21, 29.52, 37.14, 107.24, 116.26, 120.43, 128.867, 129.278, 130.97, 131.10, 137.91, 146.33, 147.42, 152.78, 162.13, 169.32, 200.10. ESI-MS calcd. for C_21_H_19_N_3_O_4_, 377.39; found: *m*/*z* 378.15 [M+H]^+^.

##### *4-(5-Acetyl-2-ethyl-3-oxo-6-phenyl-2,3-dihydro-pyridazin-4-ylamino)benzoic Acid Ethyl Ester,* **2o**

Yield = 40%; mp = 171–173 °C (EtOH); ^1^H-NMR (400 MHz, CDCl_3_) δ 1.48 (m, 6H, 3H CH_2_CH_3_ + 3H OCH_2_CH_3_), 1.80 (s, 3H, COCH_3_), 4.32 (m, 4H, 2H CH_2_CH_3_ + 2H OCH_2_CH_3_), 7.10 (d, 2H, Ar, J = 8.4 Hz), 7.45 (m, 5H, Ar), 8.00 (d, 2H, Ar, J = 8.3 Hz), 8.40 (exch br s, 1H, NH). ^13^C-NMR (100 MHz, CDCl_3_) δ 12.22, 14.18, 30.01, 37.08, 61.03, 107.32, 116.28, 120.19, 129.00, 129.45, 130.79, 131.09, 138.02, 146.31, 146.48, 152.68, 161.89, 160.11, 200.21. ESI-MS calcd. for C_23_H_23_N_3_O_4_, 405.45; found: *m*/*z* 406.15 [M+H]^+^.

##### *5-Acetyl-2-ethyl-4-(4-nitrophenylamino)-6-phenylpyridazin-3(2H)-one,* **2p**

Yield = 73%; mp = 209–211 °C (EtOH); ^1^H-NMR (400 MHz, DMSO-*d_6_*) δ 1.34 (t, 3H, CH_2_*CH_3_*, J = 7.2 Hz), 2.04 (s, 3H, COCH_3_), 4.19 (q, 2H, *CH_2_*CH_3_, J = 7.2 Hz), 7.04 (d, 2H, Ar, J = 8.4 Hz), 7.40 (m, 5H, Ar), 8.08 (d, 2H, Ar, J = 8.3 Hz), 9.46 (exch br s, 1H, NH). ^13^C-NMR (100 MHz, CDCl_3_) δ 12.18, 29.58, 37.09, 107.34, 114.64, 124.78, 128.82, 129.79, 131.08, 137.02, 137.94, 146.29, 148.31, 153.06, 162.32, 200.19. ESI-MS calcd. for C_20_H_18_N_4_O_4_, 378.38; found: *m*/*z* 379.13 [M+H]^+^.

##### *3-(5-Acetyl-2-ethyl-3-oxo-6-phenyl-2,3-dihydro-pyridazin-4-ylamino)-benzonitrile,* **2q**

Yield = 80%; mp = 204–205 °C (EtOH); ^1^H-NMR (400 MHz, CDCl_3_) δ 1.44 (t, 3H, CH_2_CH_3_, J = 7.2 Hz), 1.87 (s, 3H, COCH_3_), 4.30 (q, 2H, CH_2_CH_3_, J = 7.2 Hz), 7.06 (d, 2H, Ar, J = 8.3 Hz), 7.44 (m, 5H, Ar), 7.58 (d, 2H, Ar, J = 8.4 Hz), 8.61 (exch br s, 1H, NH). ^13^C-NMR (100 MHz, CDCl_3_) δ 12.24, 29.61, 37.16, 102.58, 107.29, 118.59, 120.31, 128.65, 129.18, 131.08, 133.11, 138.02, 146.28, 146.57, 152.65, 162.20, 200.03. ESI-MS calcd. for C_21_H_18_N_4_O_2_, 358.14; found: *m*/*z* 358.91 [M+H]^+^.

##### *5-Acetyl-4-(benzo [1,3]dioxol-5-ylamino)-2-ethyl-6-phenylpyridazin-3(2H)-one,* **2r**

Yield = 66%; mp = 189–191 °C (EtOH); ^1^H-NMR (400 MHz, CDCl_3_) δ 1.50 (t, 3H, CH_2_*CH_3_*, J = 7.2 Hz), 1.70 (s, 3H, COCH_3_), 4.30 (q, 2H, *CH_2_*CH_3_, J = 7.2 Hz), 6.00 (s, 2H, OCH_2_O), 6.60 (m, 3H, Ar), 7.40 (s, 5H, Ar), 8.01 (exch br s, 1H, NH). ^13^C-NMR (100 MHz, CDCl_3_) δ 12.25, 29.61, 37.13, 100.39, 101.22, 107.34, 109.45, 112.76, 128.78, 129.21, 131.03, 137.75, 138.01, 138.86, 146.31, 148.11, 152.72, 162.09, 200.19. ESI-MS calcd. for C_21_H_19_N_3_O_4_, 377.39; found: *m*/*z* 378.14 [M+H]^+^.

##### *5-Acetyl-4-(3-chloro-4-methoxyphenylamino)-2-ethyl-6-phenylpyridazin-3(2H)-one,* **2s**

Yield = 64%; mp = 180–182 °C (EtOH); ^1^H-NMR (400 MHz, CDCl_3_) δ 1.50 (t, 3H, CH_2_*CH_3_*, J = 7.2 Hz), 1.70 (s, 3H, COCH_3_), 3.90 (s, 3H, OCH_3_), 4.30 (q, 2H, *CH_2_*CH_3_, J = 7.2 Hz), 7.65 (m, 9H, Ar), 8.10 (exch br s, 1H, NH). ^13^C-NMR (100 MHz, CDCl_3_) δ 12.23, 29.58, 37.13, 55.40, 107.32, 115.42, 116.59, 117.68, 121.47, 128.78, 129.18, 131.03, 137.88, 138.11, 144.63, 146.32, 152.65, 162.11, 200.32. ESI-MS calcd. for C_21_H_20_ClN_3_O_3_, 397.85; found: *m*/*z* 398.64 [M+H]^+^.

#### 3.1.3. General Procedure for the Synthesis of Compounds **3a**–**i**

To a stirred solution of 5-acetyl-2-ethyl-4-nitro-6-phenylpyridazin-3(2H)-one **1** [30] (0.30 mmol) in 1–5 mL of ethanol, appropriate aryl or heteroaryl amine (0.45–0.90 mmol) was added portion-wise. The resulting mixture was stirred at room temperature for 2–5 h. For compounds **3c** and **3e,** the mixture was stirred for 24 h, and then every **3 h** for 40 h. In the case of compound **3g,** it was necessary to use a microwave oven for 2 h. After cooling, the final products were collected by filtration and washed with ethanol. For compounds **3a** and **3i,** the solvent was concentrated in vacuo, the mixture was diluted with cold water (10–15 mL), and the precipitate was recovered by suction; the final compounds were purified by column chromatography using CH_2_Cl_2_/CH_3_OH 9:1 (for **3a**) and hexane/ethyl acetate 3:1 (for **3i**) as eluents.

##### *5-Acetyl-2-ethyl-6-phenyl-4-(pyridin-3-ylamino)pyridazin-3(2H)-one,* **3a**

Yield = 26%; mp = 185–186 °C (EtOH); ^1^H-NMR (400 MHz, DMSO-*d_6_*) δ 1.34 (m, 3H, CH_2_*CH_3_*), 1.72 (s, 3H, COCH_3_), 4.18 (q, 2H, *CH_2_*CH_3_, J = 7.2 Hz), 7.29 (m, 3H, Ar), 7.41 (m, 4H, Ar), 8.26 (m, 1H, Ar), 8.33 (m,1H, Ar), 9.10 (exch br s, 1H, NH). ^13^C-NMR (100 MHz, CDCl_3_) δ 12.18, 29.67, 37.23, 109.31, 127.62, 128.83, 129.27, 131.10, 134.37, 136.32, 137.92, 129.01, 146.38, 157.29, 162.11, 200.18. ESI-MS calcd. for C_19_H_18_N_4_O_2_, 334.37; found: *m*/*z* 335.16 [M+H]^+^.

##### *5-Acetyl-2-ethyl-6-phenyl-4-(quinolin-8-ylamino)pyridazin-3(2H)-one,* **3b**

Yield = 75%; mp = 179–180 °C (EtOH); ^1^H-NMR (400 MHz, CDCl_3_) δ 1.49 (t, 3H, CH_2_*CH_3_*, J = 7.2 Hz), 1.75 (s, 3H, COCH_3_), 4.34 (q, 2H, *CH_2_*CH_3_, J = 7.2 Hz), 7.25 (m, 1H, Ar), 7.45 (m, 7H, Ar), 7.56 (m, 1H, Ar), 8.17 (d,1H, Ar, J = 8.2 Hz), 8.92 (d, 1H, Ar, J = 8.2 Hz), 9.55 (exch br s, 1H, NH). ^13^C-NMR (100 MHz, CDCl_3_) δ 12.21, 29.58, 37.09, 107.29, 113.82, 116.53, 121.18, 127.36, 128.84, 129.18, 131.09, 133.82, 136.73, 137.18, 138.01, 146.37, 148.58, 152.67, 162.18, 200.16. ESI-MS calcd. for C_23_H_20_N_4_O_2_, 384.43; found: *m*/*z* 385.17 [M+H]^+^.

##### *4-(5-Acetyl-2-ethyl-3-oxo-6-phenyl-2,3-dihydropyridazin-4-ylamino)naphthalene-1-carboxylic Acid,* **3c**

Yield = 54%; mp = 258–260 °C (EtOH); ^1^H-NMR (400 MHz, DMSO-*d_6_*) δ 1.38 (t, 3H, CH_2_*CH_3_*, J = 7.2 Hz), 1.41 (s, 3H, COCH_3_), 4.21 (q, 2H, *CH_2_*CH_3_, J = 7.2 Hz), 7.24 (m, 3H, Ar), 7.40 (m, 3H, Ar), 7.66 (m, 2H, Ar), 8.02 (d,1H, Ar, J = 7.9 Hz), 8.16 (d, 1H, Ar, J = 8.8 Hz), 8.93 (d, 1H, Ar, J = 7.9 Hz), 9.19 (exch br s, 1H, OH), 13.05 (exch br s, 1H, NH). ^13^C-NMR (400 MHz, DMSO-*d_6_*) δ 12.22, 29.58, 37.11, 107.02, 108.25, 117.69, 120.42, 124.17, 125.43, 125.47, 126.63, 128.81, 129.29, 130.11, 131.07, 131.32, 145.03, 146.36, 152.69, 162.11, 168.02, 200.11. ESI-MS calcd. for C_25_H_21_N_3_O_2_, 427.45; found: *m*/*z* 428.18 [M+H]^+^.

##### *5-Acetyl-2-ethyl-4-(6-hydroxynaphthalen-1-ylamino)-6-phenylpyridazin-3(2H)-one,* **3d**

Yield = 76%; mp = 235–236 °C (EtOH); ^1^H-NMR (400 MHz, DMSO-*d_6_*) δ 1.21 (t, 3H, CH_2_*CH_3_*, J = 7.2 Hz), 1.37 (s, 3H, COCH_3_), 4.21 (q, 2H, *CH_2_*CH_3_, J = 7.2 Hz), 6.98 (d, 1H, Ar, J = 7.0 Hz), 7.10 (m, 1H, Ar), 7.12 (s, 1H, Ar), 7.30 (m, 6H, Ar), 7.54 (d, 1H, Ar, J = 8.2 Hz), 7.83 (d, 1H, Ar, J = 8.8 Hz), 8.96 (exch br s, 1H, OH), 9.19 (exch br s, 1H, NH). ^13^C-NMR (400 MHz, DMSO-*d_6_*) δ 12.24, 29.63, 37.11, 102.87, 107.28, 116.79, 118.41, 120.03, 122.78, 128.11, 128.84, 129.23, 131.02, 131.11, 135.29, 137.91, 140.37, 146.28, 152.68, 155.02, 162.11, 200.18. ESI-MS calcd. for C_24_H_21_N_3_O_3_, 399.44; found: *m*/*z* 400.18 [M+H]^+^.

##### 5*-Acetyl-2-ethyl-4-(5-hydroxynaphthalen-1-ylamino)-6-phenylpyridazin-3(2H)-one,* **3e**

Yield = 24%; mp = 234–236 °C (EtOH); ^1^H-NMR (400 MHz, DMSO-d6) δ 1.18 (t, 3H, CH_2_*CH_3_*, J = 7.2 Hz), 1.37 (s, 3H, COCH_3_), 4.22 (q, 2H, *CH_2_*CH_3_, J = 7.2 Hz), 6.89 (m, 2H, Ar), 7.36 (m, 8H, Ar), 8.01 (d, 1H, Ar, J = 8.2 Hz), 8.95 (exch br s, 1H, OH), 10.22 (exch br s, 1H, NH). ^13^C-NMR (400 MHz, DMSO-*d_6_*) δ 12.19, 29.58, 37.12, 105.79, 107.32, 110.45, 113.72, 124.91, 125.78, 126.82, 128.01, 128.92, 129.18, 131.07, 137.93, 140.28, 146.34, 152.73, 154.67, 162.15, 200.08. ESI-MS calcd. for C_24_H_21_N_3_O_3_, 399.44; found: *m*/*z* 400.21 [M+H]^+^.

##### *5-Acetyl-2-ethyl-6-phenyl-4-(quinolin-5-ylamino)pyridazin-3(2H)-one,* **3f**

Yield = 75%; mp = 220–221 °C (EtOH); ^1^H-NMR (400 MHz, DMSO-*d_6_*) δ 1.31 (t, 3H, CH_2_*CH_3_*, J = 7.2 Hz), 1.38 (s, 3H, COCH_3_), 4.22 (q, 2H, CH_2_*CH_3_*, J = 7.2 Hz); 7.22 (m, 2H, Ar), 7.38 (m, 4H, Ar), 7.58 (m, 2H, Ar), 7.86 (d, 1H, Ar, J = 8.5 Hz), 8.43 (d, 1H, Ar, J = 7.6 Hz), 8.92 (m, 1H, Ar), 9.19 (exch br s, 1H, NH). ^13^C-NMR (400 MHz, DMSO-*d_6_*) δ 12.22, 29.60, 37.11, 107.28, 116.02, 116.18, 120.23, 121.57, 128.78, 129.18, 130.26, 131.09, 131.32, 137.93, 138.68, 141.57, 146.32, 149.59, 152.72, 162.11, 200.16. ESI-MS calcd. for C_23_H_20_N_4_O_2_, 384.43; found: *m*/*z* 385.17 [M+H]^+^.

##### *5-Acetyl-2-ethyl-4-(4-nitronaphthalen-1-ylamino)-6-phenylpyridazin-3(2H)-one,* **3g**

Yield = 11%; mp = 185–186 °C (EtOH); ^1^H-NMR (400 MHz, DMSO-*d_6_*) δ 1.37 (t, 3H, CH_2_*CH_3_*, J = 7.2 Hz), 1.71 (s, 3H, COCH_3_), 4.22 (q, 2H, *CH_2_*CH_3_, J = 7.2 Hz), 7.18 (m, 1H, Ar), 7.40 (m, 5H, Ar), 7.73 (m, 1H, Ar), 7.84 (m, 1H, Ar), 8.25 (d, 1H, Ar, J = 8.5 Hz), 8.35 (d, 1H, Ar, J = 8.5 Hz), 8.53 (m, 1H, Ar), 9.39 (exch br s, 1H, NH). ^13^C-NMR (400 MHz, DMSO-*d_6_*) δ 12.22, 29.62, 37.11, 107.28, 117.62, 121.48, 123.52, 123.81, 126.29, 126.69, 127.41, 128.80, 129.19, 129.48, 131.03, 137.26, 137.88, 146.27, 147.18, 152.65, 162.11, 200.09. ESI-MS calcd. for C_24_H_20_N_4_O_4_, 428.44; found: *m*/*z* 429.23 [M+H]^+^.

##### *5-Acetyl-2-ethyl-4-(8-hydroxyquinolin-5-ylamino)-6-phenylpyridazin-3(2H)-one,* **3h**

Yield = 90%; mp = 261–262 °C (EtOH); ^1^H-NMR (400 MHz, DMSO-*d_6_*) δ 1.25 (t, 3H, CH_2_*CH_3_*, J = 7.2 Hz), 1.37 (s, 3H, COCH_3_), 4.20 (q, 2H, CH_2_*CH_3_*, J = 7.2 Hz), 6.90 (d, 1H, Ar, J = 8.2 Hz), 7.24 (m, 6H, Ar), 7.60 (m, 1H, Ar), 8.30 (d, 1H, Ar, J = 8.2 Hz), 8.87 (m, 1H, Ar), 8.93 (exch br s, 1H, OH), 9.97 ( exch br s, 1H, NH). 13C-NMR (400 MHz, DMSO-*d_6_*) δ 12.07, 29.57, 37.18, 107.32, 112.48, 116.18, 120.73, 122.59, 128.82, 129.31, 130.71, 131.01, 134.29, 137.86, 138.05, 143.88, 146.28, 150.03, 152.67, 162.11, 200.09. ESI-MS calcd. for C_23_H_20_N_4_O_3_, 400.43; found: *m*/*z* 401.21 [M+H]^+^.

##### *5-Acetyl-2-ethyl-4-(8-fluoroquinolin-5-ylamino)-6-phenylpyridazin-3(2H)-one,* **3i**

Yield = 71%; mp = 217–218 °C (EtOH); ^1^H-NMR (400 MHz, DMSO-*d_6_*) δ 1.35 (m, 6H, 3H CH_2_*CH_3_* + 3H COCH_3_), 4.21 (q, 2H, *CH_2_*CH_3_, J = 7.2 Hz), 7.22 (m, 5H, Ar), 7.58 (m, 2H, Ar), 7.87 (d, 1H, Ar, J = 8.2 Hz), 8.43 (d, 1H, Ar, J = 8.2 Hz), 8.92 (m, 1H, Ar), 9.23 (exch br s, 1H, NH). ^13^C-NMR (400 MHz, DMSO-*d_6_*) δ 12.20, 29.62, 37.13, 107.28, 113.69, 116.27, 121.58, 123.71, 128.82, 129.18, 131.02, 131.29, 137.83, 138.01, 139.04, 146.27, 148.62, 150.25, 152.66, 162.11, 200.09. ESI-MS calcd. for C_23_H_19_FN_4_O_2_, 402.42; found: *m*/*z* 403.18 [M+H]^+^.

#### 3.1.4. Procedure for the Synthesis of 5-Acetyl-2-ethyl-4-[(1-oxidoquinolin-5-yl)amino]-6-phenylpyridazin-3(2H)-one, **3j**

A solution of compound **3f** (0.55 mmol) in dichloromethane (3 mL) was added dropwise to a cold solution of 3-chloroperoxybenzoic acid (0.55 mmol) in dichloromethane (7 mL). The mixture was stirred at room temperature for 27 h and added to a solution of KHSO_4_ in water (20 mL). The organic layer was washed with water, dried over sodium sulfate anhydride and evaporated. The crude obtained was purified by column chromatography using dichloromethane/methanol 11:0.5 as eluent.

Yield = 73%; mp = 264–265 °C (EtOH); ^1^H-NMR (400 MHz, DMSO-*d_6_*) δ 1.37 (t, 3H, CH_2_*CH_3_*, J = 7.2 Hz), 1.41 (s, 3H, COCH_3_), 4.21 (q, 2H, *CH_2_*CH_3_, J = 7.2 Hz), 7.25 (m, 2H, Ar), 7.42 (m, 3H, Ar), 7.48 (m, 2H, Ar), 7.65 (m, 1H, Ar), 7.96 (d, 1H, Ar, J = 8.2 Hz), 8.35 (d, 1H, Ar, J = 8.2), 8.61 (m, 1H, Ar), 9.24 (exch br s, 1H, NH). ^13^C-NMR (400 MHz, DMSO-*d_6_*) δ 12.22, 29.67, 37.08, 107.28, 113.10, 116.68, 120.09, 122.57, 123.78, 128.82, 129.18, 130.35, 131.06, 132.53, 138.02, 139.38, 141.23, 146.29, 152.75, 162.11, 200.04. ESI-MS calcd. for C_23_H_20_N_4_O_3_, 402.43; found: *m*/*z* 403.19 [M+H]^+^.

#### 3.1.5. General Procedure for the Synthesis of Compounds **4a**–**g**

To a stirred solution of **1** [30] (0.30 mmol) in 1–2 mL of EtOH, appropriate alkyl or cycloalkyl amine (0.30–0.90 mmol) was added portion-wise. The resulting mixture was stirred at room temperature for 30 min–4 h. After concentration of the solvent, the solid obtained was recovered by suction. For compounds **4f** and **4g**, ice-cold water was added, and the mixture was extracted with CH_2_Cl_2_ (3 × 15 mL). Evaporation of the solvent afforded compounds **4f**,**g,** which were purified by column chromatography using cyclohexane/ethyl acetate 1:1 as eluent.

##### *5-Acetyl-2-ethyl-4-ethylamino-6-phenylpyridazin-3(2H)-one,* **4a**

Yield = 30%; mp = 113–114 °C (EtOH); ^1^H-NMR (400 MHz, CDCl_3_) δ 1.30 (t, 3H, NHCH_2_*CH_3_*, J = 7.2 Hz), 1.50 (t, 3H, N*CH_2_*CH_3_, J = 7.2 Hz), 1.90 (s, 3H, COCH_3_), 3.30 (q, 2H, NH*CH_2_*CH_3_, J = 7.2 Hz), 4.31 (q, 2H, N*CH_2_*CH_3_, J = 7.2 Hz), 7.40 (s, 5H, Ar), 7.57 (exch br s, 1H, NH). ^13^C-NMR (100 MHz, CDCl_3_) δ 12.22, 15.63, 29.58, 37.09, 38.18, 109.32, 128.81, 129.23, 131.11, 137.88, 146.28, 157.72, 162.21, 200.09. ESI-MS calcd. for C_16_H_19_N_3_O_2_, 285.34; found: *m*/*z* 286.15 [M+H]^+^.

##### *5-Acetyl-2-ethyl-4-isopropylamino-6-phenylpyridazin-3(2H)-one,* **4b**

Yield = 56%; mp = 80–83 °C (EtOH); ^1^H-NMR (400 MHz, CDCl_3_) δ 1.30 (m, 6H, CH(*CH_3_*)_2_), 1.50 (t, 3H, NCH_2_*CH_3_*, J = 7.2 Hz), 1.88 (s, 3H, COCH_3_), 4.38 (m, 3H, 2H N*CH*_2_CH_3_ + 1H *CH*(CH_3_)_2_), 7.60 (s, 5H, Ar), 7.78 (exch br s, 1H, NH). ^13^C-NMR (100 MHz, CDCl_3_) δ 12.19, 23.82, 29.63, 37.08, 47.57, 109.34, 128.82, 129.19, 131.08, 138.03, 146.27, 157.72, 162.11, 200.08. ESI-MS calcd. for C_17_H_21_N_3_O_2_, 299.37; found: *m*/*z* 300.16 [M+H]^+^.

##### *5-Acetyl-4-cyclopentylamino-2-ethyl-6-phenylpyridazin-3(2H)-one,* **4c**

Yield = 38%; mp = 97–99 °C (EtOH); ^1^H-NMR (400 MHz, CDCl_3_) δ 1.41 (t, 3H, NCH_2_*CH_3_*, J = 7.2 Hz), 1.65 (m, 6H, cC_5_H_9_), 1.85 (s, 3H, COCH_3_), 1.95 (m, 2H, cC_5_H_9_), 4.21 (q, 2H, N*CH_2_*CH_3_, J = 7.2 Hz), 4.40 (m, 1H, cC_5_H_9_), 7.42 (s, 5H, Ar), 7.76 (exch br s, 1H, NH). ^13^C-NMR (100 MHz, CDCl_3_) δ 12.21, 24.01, 29.67, 33.18, 37.09, 60.32, 109.35, 128.84, 129.21, 131.03, 138.02, 146.32, 157.67, 162.11, 200.09. ESI-MS calcd. for C_19_H_23_N_3_O_2_, 325.40; found: *m*/*z* 326.12 [M+H]^+^.

##### *5-Acetyl-4-cyclohexylamino-2-ethyl-6-phenylpyridazin-3(2H)-one,* **4d**

Yield = 34%; mp = 123–125 °C (EtOH); ^1^H-NMR (400 MHz, CDCl_3_) δ 1.44 (m, 8H, 3H NCH_2_*CH_3_* + 5H cC_6_H_11_), 1.72 (m, 3H, cC_6_H_11_), 1.85 (s, 3H, COCH_3_), 2.02 (m, 2H, cC_6_H_11_), 4.28 (q, 2H, NCH_2_CH_3_, J = 7.2 Hz), 4.35 (m, 1H, cC_6_H_11_), 7.44 (s, 5H, Ar), 8.02 (exch br s, 1H, NH). ^13^C-NMR (100 MHz, CDCl_3_) δ 12.23, 25.18, 25.69, 29.61, 32.88, 37.09, 55.17, 109.27, 128.75, 129.21, 131.04, 137.88, 146.27, 157.65, 162.08, 200.09. ESI-MS calcd. for C_20_H_25_N_3_O_2_, 339.43; found: *m*/*z* 340.19 [M+H]^+^.

##### *5-Acetyl-4-cycloheptylamino-2-ethyl-6-phenylpyridazin-3(2H)-one,* **4e**

Yield = 36%; mp = 98–100 °C (EtOH); 1H-NMR (400 MHz, CDCl_3_) δ 1.40 (t, 3H, NCH_2_*CH_3_*, J = 7.2 Hz), 1.62 (m, 8H, cC_7_H_13_), 1.83 (s, 3H, COCH_3_), 1.98 (m, 4H, cC_7_H_13_), 4.15 (m, 3H, 2H N*CH_2_*CH_3_ + 1H cC_7_H_13_), 7.41 (s, 5H, Ar), 8.02 (exch br s, 1H, NH). ^13^C-NMR (100 MHz, CDCl_3_) δ 12.21, 25.48, 29.02, 29.59, 32.42, 37.09, 54.62, 109.28, 128.81, 129.18, 131.03, 137.79, 146.31, 157.68, 162.11, 200.09. ESI-MS calcd. for C_21_H_27_N_3_O_2_, 353.46; found: *m*/*z* 354.23 [M+H]^+^.

##### *5-Acetyl-2-ethyl-4-hexylamino-6-phenylpyridazin-3(2H)-one,* **4f**

Yield = 27%; oil; 1H-NMR (400 MHz, CDCl_3_) δ 0.91 (t, 3H, *CH_3_*(CH_2_)_5_, J = 7.2 Hz), 1.36 (m, 9H, 6H -(*CH_2_*)_3_CH_3_ + 3H CH_2_*CH_3_*), 1.68 (m, 2H, NHCH_2_*CH_2_*), 1.85 (s, 3H, COCH_3_), 3.25 (m, 2H, NH*CH_2_*CH_2_), 4.22 (q, 2H, N*CH_2_*CH_3_, J = 7.2 Hz), 7.42 (s, 5H, Ar), 8.15 (exch br s, 1H, NH). ^13^C-NMR (100 MHz, CDCl_3_) δ 12.19, 14.11, 22.76, 26.81, 29.57, 30.59, 31.50, 37.18, 44.38, 109.28, 128.81, 128.17, 131.03, 137.88, 146.28, 157.71, 162.11, 200.08. ESI-MS calcd. for C_20_H_27_N_3_O_2_, 341.45; found: *m*/*z* 342.20 [M+H]^+^.

##### *5-Acetyl-4-butylamino-2-ethyl-6-phenylpyridazin-3(2H)-one,* **4g**

Yield = 48%; mp = 67–69 °C (EtOH); ^1^H-NMR (400 MHz, CDCl_3_) δ 0.95 (t, 3H, *CH_3_*(CH_2_)_3_, J = 7.2 Hz), 1.42 (t, 3H, NCH_2_*CH_3_*, J = 7.2 Hz), 1.68 (m, 4H, (*CH_2_*)_2_CH_3_), 1.88 (s, 3H, COCH_3_), 3.23 (m, 2H, NH*CH_2_*CH_2_), 4.25 (q, 2H, N*CH_2_*CH_3_, J = 7.2 Hz), 7.40 (s, 5H, Ar), 8.35 (exch br s, 1H, NH). ^13^C-NMR (100 MHz, CDCl_3_) δ 12.18, 13.78, 20.21, 29.58, 32.79, 37.11, 44.18, 109.31, 128.81, 129.24, 131.03, 137.92, 146.32, 157.67, 162.11, 200.14. ESI-MS calcd. for C_18_H_23_N_3_O_2_, 313.39; found: *m*/*z* 314.16 [M+H]^+^.

#### 3.1.6. General Procedure for Compounds **5a**–**l**

To a stirred solution of **1** [30] (0.40 mmol) in 1–2 mL of EtOH, appropriate aryl- or hetero-arylalkylamine (0.40–0.80 mmol) was added portion-wise. The resulting mixture was stirred at room temperature for 0.5–2 h. For compound **5d**, instead, the suspension was heated at 90 °C for 3 h. After cooling, the solvent was concentrated in vacuo, and the precipitate was recovered by suction. For compounds **5d**, **5f** and **5j**, after evaporation of the solvent, cold water was added (20 mL) and the mixture was extracted with CH_2_Cl_2_ (3 × 15 mL) and organic layer was evaporated resulting in desired final compounds which were purified by column chromatography using toluene/ethyl acetate 8:2 (for **5f**) and cyclohexane/ethyl acetate 1:2 (for **5j**) as eleunts. Compound **5d** was purified by crystallization from ethanol.

##### *5-Acetyl-2-ethyl-4-(4-methoxybenzylamino)-6-phenylpyridazin-3(2H)-one,* **5a**

Yield = 57%; mp = 106–107 °C (EtOH); ^1^H-NMR (400 MHz, CDCl_3_) δ 1.40 (t, 3H, NCH_2_*CH_3_*, J = 7.2 Hz), 1.55 (s, 3H, COCH_3_), 3.82 (s, 3H, OCH_3_), 4.25 (q, 2H, N*CH_2_*CH_3_, J = 7.2 Hz), 4.40 (d, 2H, NH*CH_2_*, J = 6.2 Hz), 6.85 (d, 2H, Ar, J = 8.4 Hz), 7.15 (d, 2H, Ar, J = 8.3 Hz), 7.40 (s, 5H, Ar), 8.86 (exch br m, 1H, NH). ^13^C-NMR (100 MHz, CDCl_3_) δ 12.20, 29.63, 37.18, 44.08, 55.78, 109.28, 114.09, 128.81, 129.23, 130.18, 130.53, 131.15, 137.91, 157.72, 158.57, 162.11, 200.08. ESI-MS calcd. for C_22_H_23_N_3_O_3_, 377.44; found: *m*/*z* 378.18 [M+H]^+^.

##### *5-Acetyl-4-(4-chlorobenzylamino)-2-ethyl-6-phenylpyridazin-3(2H)-one,* **5b**

Yield = 59%; mp = 131–133 °C (EtOH); ^1^H-NMR (400 MHz, CDCl_3_) δ 1.38 (t, 3H, CH_2_*CH_3_*, J = 7.2 Hz), 1.52 (s, 3H, COCH_3_), 4.25 (q, 2H, *CH_2_*CH_3_, J = 7.2 Hz), 4.60 (d, 2H, NH*CH_2_*, J = 6.2 Hz), 7.24 (m, 4H, Ar), 7.45 (s, 5H, Ar), 7.78 (exch br m, 1H, NH). ^13^C-NMR (100 MHz, CDCl_3_) δ 12.18, 29.57, 37.09, 44.11, 109.27, 128.61, 128.82, 129.18, 131.02, 132.33, 134.57, 136.04, 137.93, 146.27, 157.65, 162.11, 200.07. ESI-MS calcd. for C_21_H_20_ClN_3_O_2_, 381.86; found: *m*/*z* 382.57 [M+H]^+^.

##### *5-Acetyl-4-[2-(3,4-dimethoxyphenyl)ethylamino]-2-ethyl-6-phenylpyridazin-3(2H)-one,* **5c**

Yield = 48%; mp = 111–112 °C (EtOH); ^1^H-NMR (400 MHz, CDCl_3_) δ 1.39 (t, 3H, CH_2_*CH_3_*, J = 7.2 Hz), 1.85 (s, 3H, COCH_3_), 2.88 (t, 2H, NHCH_2_*CH_2_*, J = 7.1 Hz), 3.59 (m, 2H, NH*CH*_2_CH_2_), 3.85 (s, 3H, OCH_3_), 3.90 (s, 3H, CH_3_), 4.25 (q, 2H, *CH_2_*CH_3_, J = 7.2 Hz), 6.80 (m, 3H, Ar), 7.41 (s, 5H, Ar), 8.98 (exch br m, 1H, NH). ^13^C-NMR (100 MHz, CDCl_3_) δ 12.18, 29.61, 36.11, 37.09, 44.68, 56.12, 109.28, 112.31, 112.79, 128.78, 129.19, 130.84, 131.11, 138.01, 146.34, 147.08, 149.71, 157.68, 162.11, 200.13. ESI-MS calcd. for C_24_H_27_ClN_3_O_4_, 421.49; found: *m*/*z* 422.20 [M+H]^+^.

##### *4-[(5-Acetyl-2-ethyl-3-oxo-6-phenyl-2,3-dihydropyridazin-4-ylamino)-methyl]benzoic Acid,* **5d**

Yield = 52%; mp = 193–195 °C (EtOH); ^1^H-NMR (400 MHz, CDCl_3_) δ 1.41 (t, 3H, CH_2_*CH_3_*, J = 7.2 Hz), 1.50 (s, 3H, COCH_3_), 4.25 (q, 2H, *CH_2_*CH_3_, J = 7.2 Hz), 4.65 (d, 2H, NH*CH_2_*, J = 6.4 Hz), 7.32 (m, 7H, Ar), 7.85 (exch br m, 1H, NH), 8.05 (d, 2H, Ar, J = 8.3 Hz). ^13^C-NMR (100 MHz, CDCl_3_) δ 12.19, 29.61, 37.11, 44.05, 109.28, 126.83, 128.21, 128.82, 129.18, 130.11, 131.08, 137.8, 143.18, 146.32, 157.72, 162.09, 169.31, 200.11. ESI-MS calcd. For C_22_H_21_N_3_O_4_, 391.42; found: *m*/*z* 392.18 [M+H]^+^.

##### *5-Acetyl-2-ethyl-4-(2-methoxybenzylamino)-6-phenylpyridazin-3(2H)-one,* **5e**

Yield = 47%; mp = 97–99 °C (EtOH); ^1^H-NMR (400 MHz, CDCl_3_) δ 1.40 (t, 3H, CH_2_*CH_3_*, J = 7.2 Hz), 1.70 (s, 3H, COCH_3_), 3.85 (s, 3H, OCH_3_), 4.20 (q, 2H, *CH_2_*CH_3_, J = 7.2 Hz), 4.60 (d, 2H, NH*CH_2_*, J = 6.4 Hz), 6.93 (m, 2H, Ar), 7.20 (m, 2H, Ar), 7.41 (s, 5H, Ar), 7.70 (exch br m, 1H, NH). ^13^C-NMR (100 MHz, CDCl_3_) δ 12.22, 29.58, 37.11, 41.76, 56.09, 109.32, 112.08, 120.77, 127.34, 127.68, 128.02, 128.85, 129.18, 131.11, 156.48, 157.68, 162.08, 200.12. ESI-MS calcd. for C_22_H_23_N_3_O_3_, 377.44; found: *m*/*z* 378.19 [M+H]^+^.

##### *5-Acetyl-2-ethyl-4-(methylphenylamino)-6-phenylpyridazin-3(2H)-one,* **5f**

Yield = 86%; oil; 1H-NMR (400 MHz, CDCl_3_) δ 1.46 (t, 3H, CH_2_*CH_3_*, J = 7.2 Hz), 1.96 (s, 3H, COCH_3_), 3.32 (s, 3H, NCH_3_), 4.32 (q, 2H, *CH_2_*CH_3_, J = 7.2 Hz), 6.82 (d, 2H, Ar, J = 7.6 Hz), 6.93 (t, 1H, Ar, J = 7.2 Hz), 7.26 (m, 2H, Ar), 7.46 (s, 4H, Ar), 7.56 (s, 1H, Ar). ^13^C-NMR (100 MHz, CDCl_3_) δ 12.24, 29.61, 37.16, 39.58, 107.29, 121.00, 125.09, 128.82, 129.28, 129.59, 131,02, 138.02, 146.27, 148.03, 152.66, 162.11, 200.08. ESI-MS calcd. for C_21_H_21_N_3_O_2_, 347.41; found: *m*/*z* 348.19 [M+H]^+^.

##### *5-Acetyl-2-ethyl-6-phenyl-4-[(pyridin-3-ylmethyl)-amino]pyridazin-3(2H)-one,* **5g**

Yield = 57%; mp = 163–164 °C (EtOH); ^1^H-NMR (400 MHz, CDCl_3_) δ 1.42 (t, 3H, CH_2_*CH_3_*, J = 7.2 Hz), 1.57 (s, 3H, COCH_3_), 4.24 (q, 2H, *CH_2_*CH_3_, J = 7.2 Hz), 4.72 (d, 2H, NH*CH_2_*, J = 6.5 Hz), 7.20–7.43 (m, 6H, Ar), 7.64 (d, 1H, Ar, J = 7.9 Hz), 7.92 (t, 1H, Ar, J = 7.7 Hz), 8.57 (m, 2H, 1H Ar + 1H NH). ^13^C-NMR (100 MHz, CDCl_3_) δ 12.19, 29.63, 37.09, 47.68, 109.28, 123.07, 128.83, 129.18, 131.03, 135.52, 135.67, 138.03, 146.29, 147.28, 148.77, 157.69, 162.13, 200.09. ESI-MS calcd. for C_20_H_20_N_4_O_2_, 348.40; found: *m*/*z* 349.18 [M+H]^+^.

##### *5-Acetyl-2-ethyl-6-phenyl-4-[(pyridin-2-ylmethyl)-amino]pyridazin-3(2H)-one,* **5h**

Yield = 42%; mp = 138–140 °C (EtOH); 1H-NMR (400 MHz, CDCl_3_) δ 1.43 (t, 3H, CH_2_*CH_3_*, J = 7.2 Hz), 1.81 (s, 3H, COCH_3_), 4.30 (q, 2H, *CH_2_*CH_3_, J = 7.2 Hz), 4.70 (d, 2H, NH*CH_2_*, J = 6.4 Hz), 7.52 (m, 8H, Ar), 8.68 (d, 1H, Ar, J = 7.9 Hz), 8.78 (exch br m, 1H, NH). ^13^C-NMR (100 MHz, CDCl_3_) δ 12.19, 29.58, 37.12, 52.68, 109.31, 121.03, 124.14, 128.82, 129.19, 131.04, 137.89, 139.59, 146.28, 148.57, 156.11, 157.65, 162.13, 200.14. ESI-MS calcd. for C_20_H_20_N_4_O_2_, 348.40; found: *m*/*z* 349.17 [M+H]^+^.

##### *5-Acetyl-2-ethyl-6-phenyl-4-[(pyridin-4-ylmethyl)amino]pyridazin-3(2H)-one,* **5i**

Yield = 50%; mp = 150–151 °C (EtOH); ^1^H-NMR (400 MHz, CDCl_3_) δ 1.40 (t, 3H, CH_2_*CH_3_*, J = 7.2 Hz), 1.55 (s, 3H, COCH_3_), 4.24 (q, 2H, *CH_2_*CH_3_, J = 7.2 Hz), 4.75 (d, 2H, NH*CH_2_*, J = 6.3 Hz), 7.32 (m, 7H, Ar), 7.95 (d, 2H, Ar, J = 7.9 Hz), 8.70 (exch br m, 1H, NH). ^13^C-NMR (100 MHz, CDCl_3_) δ 12.23, 29.61, 37.11, 49.09, 109.28, 122.41, 128.82, 129.18, 131.03, 137.88, 146.28, 147.58, 149.78, 157.68, 162.09, 200.03. ESI-MS calcd. for C_20_H_20_N_4_O_2_, 348.40; found: *m*/*z* 349.18 [M+H]^+^.

##### *5-Acetyl-2-ethyl-6-phenyl-4-(2-pyridin-2-yl-ethylamino)pyridazin-3(2H)-one,* **5j**

Yield = 90%; oil; ^1^H-NMR (400 MHz, CDCl_3_) δ 1.38 (t, 3H, CH_2_*CH_3_*, J = 7.2 Hz), 1.86 (s, 3H, COCH_3_), 3.16 (t, 3H, NHCH_2_*CH_2_*, J = 7.1 Hz), 3.73 (m, 2H, NH*CH_2_*CH_2_), 4.20 (q, 2H, *CH_2_*CH_3_, J = 7.2 Hz), 7.20 (m, 2H, Ar), 7.39 (s, 5H, Ar), 7.63 (t, 1H, Ar, J = 7.8 Hz), 7.79 (exch br m, 1H, NH), 8.69 (d, 1H, Ar, J = 7.8 Hz). ^13^C-NMR (100 MHz, CDCl_3_) δ 12.23, 29.58, 36.21, 37.14, 44.69, 109.32, 121.04, 128.82, 129.19, 122.93, 131.11, 136.38, 137.88, 146.27, 148.31, 157.68, 158.71, 162.11, 200.09. ESI-MS calcd. for C_21_H_22_N_4_O_2_, 362.42; found: *m*/*z* 363.17 [M+H]^+^.

##### *5-Acetyl-2-ethyl-6-phenyl-4-[(thiophen-3-ylmethyl)-amino]pyridazin-3(2H)-one,* **5k**

Yield = 71%; mp = 133–135 °C (EtOH); ^1^H-NMR (400 MHz, CDCl_3_) δ 1.40 (t, 3H, CH_2_*CH_3_*, J = 7.2 Hz), 1.58 (s, 3H, COCH_3_), 4.22 (q, 2H, *CH_2_*CH_3_, J = 7.2 Hz), 4.58 (d, 2H, NH*CH_2_*, J = 6.5 Hz), 6.98 (m, 2H, Ar), 7.10 (m, 1H, Ar), 7.25 (m, 5H, Ar), 7.60 (exch br s, 1h, NH). 13C-NMR (100 MHz, CDCl_3_) δ 12.18, 29.62, 37.13, 42.88, 109.28, 121.31, 126.08, 128.11, 128, 89, 129.18, 131.02, 137.45, 137.88, 146.28, 157.69, 162.11, 200.12. ESI-MS calcd. for C_19_H_19_N_3_O_2_S, 353.44; found: *m*/*z* 354.18 [M+H]^+^.

##### *5-Acetyl-2-ethyl-4-[(4-methylisoxazol-3-ylmethyl)-amino]-6-phenylpyridazin-3(2H)-one,* **5l**

Yield = 47%; mp = 113–115 °C (EtOH); ^1^H-NMR (400 MHz, CDCl_3_) δ 1.40 (t, 3H, CH_2_*CH_3_*, J = 7.2 Hz), 1.80 (s, 3H, COCH_3_), 2.40 (s, 3H, CH_3_), 4.20 (q, 2H, *CH_2_*CH_3_, J = 7.2 Hz), 4.80 (d, 2H, NH*CH_2_*, J = 6.4 Hz), 6.00 (s, 2H, Ar), 7.43 (s, 5H, Ar), 8.20 (exch br m, 1H, NH). ^13^C-NMR (100 MHz, CDCl_3_) δ 11.58, 12.21, 29.62, 37.09, 44.38, 100.52, 109.29, 128.82, 129.19, 131.03, 137.88, 146.32, 150.11, 154.68, 157.74, 162.18, 200.09. ESI-MS calcd. for C19H20N4O3, 352.39; found: *m*/*z* 353.15 [M+H]^+^.

#### 3.1.7. Synthesis of Compound 5-Acetyl-4-amino-2-ethyl-6-phenylpyridazin-3(2H)-one, **7**

A mixture of 6-ethyl-3-methyl-4-phenylisoxazolo [3,4-d]pyridazin-7(6H)-one **6** (0.78 mmol) [32], 10% Pd/C (50 mg) and ammonium formate (2.5 mmol) in ethanol (4 mL) was refluxed for 1 h. After the addition of CH_2_Cl_2_ (5 mL), the catalyst was filtered off, and the solvent was removed under reduced pressure to yield compound **7**.

Yield = 89%; mp = 149–151 °C (EtOH); ^1^H-NMR (400 MHz, CDCl_3_) δ 1.41 (t, 3H, CH_2_*CH_3_*, J = 7.2 Hz), 1.78 (s, 3H, COCH_3_), 4.24 (q, 2H, *CH_2_*CH_3_, J = 7.2 Hz), 7.42 (s, 5H, Ar), 8.20 (exch br s, 2H, NH_2_). ^13^C-NMR (100 MHz, CDCl_3_) δ 12.23, 29.59, 37.12, 109.28, 128.81, 129.18, 131.07, 137.91, 144.04, 146.29, 162.11, 200.10. ESI-MS calcd. for C_14_H_15_N_3_O_2_, 257.29; found: *m*/*z* 258.11 [M+H]^+^.

#### 3.1.8. General Procedure for Compounds **2t–w** and **3l**

A mixture of compound **7** (0.97 mmol), appropriate arylboronic acid (1.94 mmol), anhydrous cupric acetate (1.46 mmol), triethylamine (1.94 mmol), and activated molecular sieves (720 mg, 4 Å) in dry dichloromethane (12 mL) was stirred at room temperature for 24 h. The reaction was filtered, and the solvent was removed under reduced pressure. The resulting residue was purified by flash column chromatography using dichloromethane/ethyl acetate (8:2) as eluent.

##### *5-Acetyl-2-ethyl-4-(3-nitrophenylamino)-6-phenylpyridazin-3(2H)-one,* **2t**

Yield = 40%; mp = 242–243 °C (EtOH); ^1^H-NMR (400 MHz, CDCl_3_) δ 1.45 (t, 3H, CH_2_*CH_3_*, J = 7.2 Hz), 1.78 (s, 3H, COCH_3_), 4.31 (q, 2H, *CH_2_*CH_3_, J = 7.2 Hz), 7.45 (m, 7H, Ar), 7.84 (s, 1H, Ar), 8.02 (d, 1H, Ar, J = 7.8), 8.73 (exch br s, 1H, NH). ^13^C-NMR (100 MHz, CDCl_3_) δ 12.0, 29.7, 37.1, 107.3, 109.2, 113.9, 122.5, 128.9, 129.2, 130.4, 131.0, 138.1, 145.3, 146.3, 148.7, 152.6, 162.1, 200.2. ESI-MS calcd. for C_20_H_18_N_4_O_4_, 378.38; found: *m*/*z* 379.14 [M+H]^+^.

##### *5-Acetyl-4-(2-chlorophenylamino)-2-ethyl-6-phenylpyridazin-3(2H)-one,* **2u**

Yield = 43%; mp = 177–178 °C (EtOH); ^1^H-NMR (400 MHz, CDCl_3_) δ 1.32 (t, 3H, CH_2_*CH_3_*, J = 7.2 Hz), 1.57 (s, 3H, COCH_3_), 4.16 (q, 2H, *CH_2_*CH_3_, J = 7.2 Hz), 7.20 (m, 3H, Ar), 7.28 (m, 2H, Ar), 7.40 (m, 3H, Ar), 7.46 (m, 1H, Ar), 8.75 (exch br s, 1H, NH). ^13^C-NMR (100 MHz, CDCl_3_) δ 12.2, 29.8, 36.9, 107.3, 120.2, 123.7, 124.9, 125.2, 128.8, 129.2, 130.9, 131.1, 137.9, 143.1, 146.3, 152.7, 162.1, 200.1. ESI-MS calcd. for C_20_H_18_ClN_3_O_2_, 367.83; found: *m*/*z* 368.58 [M+H]^+^.

##### *5-Acetyl-2-ethyl-4-(2-methoxyphenylamino)-6-phenylpyridazin-3(2H)-one,* **2v**

Yield = 30%; mp = 205–207 °C (EtOH); ^1^H-NMR (400 MHz, DMSO-*d_6_*) δ 1.33 (t, 3H, CH_2_*CH_3_*, J = 7.2 Hz), 1.52 (s, 3H, COCH_3_), 3.73 (s, 3H, CH_3_), 4.16 (q, 2H, *CH_2_*CH_3_, J = 7.2 Hz), 6.85 (t, 1H, Ar, J = 7.8 Hz), 6.97 (d, 1H, Ar, J = 8.1 Hz), 7.02 (d, 1H, Ar, J = 7.8 Hz), 7.14 (t, 1H, Ar, J = 8.2 Hz), 7.26 (m, 2H, Ar), 7.36 (m, 3H, Ar), 8.47 (exch br s, 1H, NH). ^13^C-NMR (100 MHz, CDC_l3_) δ 12.0, 29.7, 37.1, 55.8, 107.5, 113.4, 115.1, 121.8, 122.7, 128.8, 129.3, 130.9, 132.7, 137.8, 144.1, 146.4, 152,8, 162.0, 200.1. ESI-MS calcd. for C_21_H_21_N_3_O_3_, 363.41; found: *m*/*z* 364.16 [M+H]^+^.

##### *5-Acetyl-4-(3,5-difluorophenylamino)-2-ethyl-6-phenylpyridazin-3(2H)-one,* **2w**

Yield = 54%; mp = 243–245 °C (EtOH); 1H-NMR (400 MHz, CDCl_3_) δ 1.44 (t, 3H, CH_2_*CH_3_*, J = 7.2 Hz), 1.80 (s, 3H, COCH_3_), 4.31 (q, 2H, *CH_2_*CH_3_, J = 7.2 Hz), 6.59 (m, 3H, Ar), 7.40 (m, 5H, Ar), 8.26 (exch br s, 1H, NH). ^13^C-NMR (100 MHz, CDCl_3_) δ 12.18, 29.64, 37.19, 94.74, 106.78, 107.32, 128.83, 129.29, 131.03, 138.02, 146.29, 147.62, 152.71, 158.48, 162.11, 200.20. ESI-MS calcd. for C_21_H_21_N_3_O_3_, 369.36; found: *m*/*z* 370.13 [M+H]^+^.

##### *5-Acetyl-2-ethyl-4-(naphthalen-1-ylamino)-6-phenylpyridazin-3(2H)-one,* **3l**

Yield = 51%; mp = 196–198 °C (EtOH); 1H-NMR (400 MHz, CDCl_3_) δ 1.23 (t, 3H, CH_2_*CH_3_*, J = 7.2 Hz), 1.52 (s, 3H, COCH_3_), 4.36 (q, 2H, *CH_2_*CH_3_, J = 7.2 Hz), 6.85 (t, 1H, Ar, J = 7.9 Hz), 7.34 (m, 6H, Ar), 7.58 (m, 2H, Ar), 7.75 (d, 1H, Ar, J = 7.8 Hz), 7.86 (d, 1H, Ar, J = 7.8 Hz), 8.12 (d, 1H, Ar, J = 8.1 Hz), 8.27 (exch br s, 1H, NH). ^13^C-NMR (100 MHz, CDCl_3_) δ 12.21, 29.63, 37.11, 105.36, 107.29, 118.92, 121.02, 124.68, 125.21, 126.11, 127.65, 128.61, 128.82, 129.25, 130.98, 134.29, 137.92, 140.74, 146.28, 152.66, 162.11, 200.09. ESI-MS calcd. for C_24_H_21_N_3_O_2_, 383.44; found: *m*/*z* 384.18 [M+H]^+^.

#### 3.1.9. Synthesis of Compound 5-Acetyl-2-ethyl-4-(4-methylpyridin-3-ylamino)-6-phenylpyridazin-3(2H)-one, **3k**

A mixture of compound **7** (0.77 mmol), 3-bromo-4-methylpyridine (1.55 mmol), anhydrous cuprous iodide (0.05 mmol), and potassium carbonate (1.16 mmol) was stirred at 145 °C for 12 h. After cooling, cold water was added, and the mixture was extracted with ethyl acetate. The organic layer was washed with water and brine, then dried, and the solvent was removed in vacuo. The solid thus obtained was thoroughly washed with warm ethyl ether and recrystallized from methanol to yield the desired final product.

Yield = 34%; mp = 215–216 °C (EtOH); ^1^H-NMR (400 MHz, DMSO-*d_6_*) δ 1.36 (t, 3H, CH_2_*CH_3_*, J = 7.2 Hz), 1.43 (s, 3H, COCH_3_), 2.22 (s, 3H, CH_3_Ar), 4.18 (q, 2H, *CH_2_*CH_3_, J = 7.2 Hz), 7.25 (m, 3H, Ar), 7.40 (m, 3H, Ar), 8.22 (s, 1H, Ar), 8.25 (d, 1H, Ar, J = 5.0 Hz), 8.76 (exch br s, 1H, NH). ^13^C-NMR (100 MHz, CDCl_3_) δ 12.19, 17.72, 29.63, 37.20, 109.28, 125.17, 128.65, 129.27, 130.93, 132.18, 137.26, 138.23, 139.41, 145.88, 146.57, 157,32, 162.11, 200.12. ESI-MS calcd. for C_20_H_20_N_4_O_2_, 348.40; found: *m*/*z* 349.17 [M+H]^+^.

#### 3.1.10. Synthesis of Compound 5-Acetyl-2-ethyl-4-(3-fluorophenyl)-6-phenylpyridazin-3(2H)-one, **9**

A mixture of compound **8** (0.47 mmol), 0.001 mmol of tetrakis (triphenylphosphine) palladium (0), 1.41 mmol of 3-fluorophenyl boronic acid, and 2M Na_2_CO_3_ (1 mL) in anhydrous toluene (4 mL) was stirred under N_2_, at 100 °C for 10 h. After cooling, the mixture was concentrated in a vacuum, diluted with water (10 mL), and extracted with ethyl acetate (3 × 10 mL). Evaporation of the solvent afforded compound **9,** which was purified by column chromatography using cyclohexane/ethyl acetate (1:1) as eluent. Yield = 36%; mp = 127–128 °C (EtOH); 1H-NMR (400 MHz, CDCl_3_) δ 1.43 (t, 3H, CH_2_*CH_3_*, J = 7.2 Hz), 1.90 (s, 3H, COCH_3_), 4.38 (q, 2H, *CH_2_*CH_3_, J = 7.2 Hz), 7.15 (m, 4H, Ar), 7.43 (s, 5H, Ar). ^13^C-NMR (100 MHz, CDCl_3_) δ 12.18, 29.92, 37.38, 111.26, 114.68, 124.48, 128,78, 129.19, 130.29, 131.12, 134.11, 138.02, 138.83, 146.32, 148.78, 162.81, 163.59, 198.78. ESI-MS calcd. for C_20_H_17_FN_2_O_2_, 336.36; found: *m*/*z* 337.13 [M+H]^+^.

#### 3.1.11. General Procedure for Compounds **10a**,**b**

A mixture of **1** (0.5 mmol) [30] and commercially available sodium phenoxide (for compound 10a) or sodium thiophenolate (0.5 mmol) (for compound **10b**), in anhydrous ethanol (3–5 mL) was stirred at room temperature for 2 h. After dilution with water, the suspension was extracted with CH_2_Cl_2_ (3 × 15 mL), and the organic layer was evaporated in vacuum to afford the desired final compounds, which were purified by flash chromatography using toluene/ethyl acetate 8:2 as eluent.

##### *5-Acetyl-2-ethyl-4-phenoxy-6-phenylpyridazin-3(2H)-one,* **10a**

Yield = 64%; mp = 90–93 °C (EtOH); ^1^H-NMR (400 MHz, CDCl_3_) δ 1.44 (t, 3H, CH_2_*CH_3_*, J = 7.2 Hz), 2.33 (s, 3H, COCH_3_), 4.30 (q, 2H, *CH_2_*CH_3_, J = 7.2 Hz), 7.04 (d, 2H, Ar, J = 8.4 Hz), 7.15 (t, 1H, Ar, J = 7.6 Hz), 7.35 (t, 2H, Ar, J = 8.0 Hz), 7.50 (m, 5H, Ar). ^13^C-NMR (100 MHz, CDCl_3_) δ 12.23, 29.19, 36.72, 125.48, 128.79, 129.03, 129.19, 129.48, 131.07, 132.38, 135.08, 137.92, 146.30, 152.29, 157.42, 198.77. ESI-MS calcd. for C_20_H_18_N_2_O_3_, 334.37; found: *m*/*z* 335.14 [M+H]^+^.

##### *5-Acetyl-2-ethyl-6-phenyl-4-phenylsulfanylpyridazin-3(2H)-one,* **10b**

Yield = 68%; mp = 107–109 °C (EtOH); ^1^H-NMR (400 MHz, CDCl_3_) δ 1.41 (t, 3H, CH_2_*CH_3_*, J = 7.2 Hz), 2.12 (s, 3H, COCH_3_), 4.27 (q, 2H, CH_2_CH_3_, J = 7.2 Hz), 7.32 (m, 3H, Ar), 7.43 (m, 7H, Ar). ^13^C-NMR (100 MHz, CDCl_3_) δ 12.21, 29.91, 37.39, 117.18, 121.42, 122.88, 128.82, 129.18, 129.47, 131.08, 137.92, 146.28, 156.18, 157.68, 162.31, 198.80. ESI-MS calcd. for C_20_H_18_N_2_O_2_S, 350.43; found: *m*/*z* 351.12 [M+H]^+^.

### 3.2. Pharmacology

#### 3.2.1. PDE4 Activity Determination

PDE4 activity from various human recombinant PDE4 subtypes (PDE4B1, PDE4A4, and PDE4D3) was monitored by measuring the hydrolysis of [^3^H]-cAMP to [^3^H]-AMP using a PDE-SPA kit from Amersham International as previously described [43]. Enzyme extracts (∼4 μg of protein) were incubated in “low binding” plates (Costar 3604) for 60 min at room temperature. The assay mixture (80 μL) contained 15 nM [^3^H]-cAMP (1 μCi/mL) in assay buffer (50 mM Tris, pH 7.5, 8.3 mM MgCl_2_, 1.7 mM EGTA) and 10 μL of test compound. These compounds were resuspended in DMSO (final DMSO concentration 5% (*v*/*v*)) at a stock concentration of 1 mM. The compounds were tested at different concentrations varying from 10 μM to 10 pM to calculate an IC_50_. These dilutions were done in 96-well plates. In some cases, plates containing diluted compounds were frozen before being assayed. In these cases, the plates were thawed at room temperature and agitated for 15 min. Hydrolysis of [^3^H]-cAMP was initiated by adding 10 μL of a solution containing PDE4 enzyme, and the plate was then incubated under agitation at room temperature. The reaction was stopped after 60 min (with ~10–20% substrate conversion) by the addition of 50 μL of Phosphodiesterase Scintillation Proximity Assay (SPA) Beads. All reactions were carried out in duplicate. [^3^H]-AMP, captured by the SPA beads, was determined by counting the plates in a Wallac Microbeta Trilux scintillation counter 1 h after addition of the beads, although the signal was quite stable, and samples may be counted from 1 to 48 h after bead addition. All experiments were run in duplicates as long as one was within twice the value of the other (SD pIC_50_ < 0.2). Otherwise, further experiments were run until pIC_50_ < 0.2. All data reported are to two significant figures.

#### 3.2.2. [^3^H]Rolipram Displacement

The binding of [^3^H]Rolipram to rat brain membranes was performed according to Schneider et al. [44]. At least six drug concentrations were assayed in duplicate to generate individual displacement curves. IC_50_ values were calculated for those curves by nonlinear regression using the program Inplot, from GraphPad Software (version 10.6). The effect of drug vehicle was taken into account in the calculation.

#### 3.2.3. LPS Induced TNF-α in Human Whole Blood (HWB-TNF-α)

Human whole blood of healthy donors was collected in 50 mL Falcon tubes with heparin (5000 units/mL, Heparin Mayne 5%, MAYNE PHARMA). LPS (lipopolysaccharide from *Escherichia coli*, Sigma, St. Louis, MO, USA) dissolved in PBS (Dulbecco’s phosphate buffered saline, without calcium and magnesium chloride, Sigma, St. Louis, MO, USA) was added to the tubes to give a final concentration in the assay of 1 μg/mL and preincubated at 37 °C for 10 min with rocking. Increasing concentrations of different inhibitors (2 μL), dissolved in 100% DMSO, were added to the 96-well plates, and 200 μL of blood containing LPS (except for controls) was then distributed into wells. Plates were shaken for 1−2 min, sealed with an aluminum foil lid (Beckman Coulter), and then incubated for 24 h at 37 °C under agitation in KelvitronT (Heraeus Instruments). After 24 h, plates were placed on ice, 50 μL of PBS was added, and the reaction was stopped by centrifugation of plates at 2000 rpm (800× *g*) at 4 °C for 15 min. The Serum obtained was then subjected to ELISA or kept at −80 °C until use.

#### 3.2.4. LPS Induced TNF-α in Human Peripheral Blood Mononuclear Cells (HPBMC-TNF-α)

PBMCs from healthy volunteers were isolated by centrifugation of whole blood on Histopaque^®^-1077 (Sigma; Poole, Dorset, UK) at 400× *g* for 30 min at room temperature. Cells collected from the interphase were washed with PBS, then resuspended in RPMI-1640 medium supplemented with 10% heat-inactivated fetal bovine serum and 2 mL L-glutamine. Purified PBMCs were pre-treated with pharmacological agents for 60 min before stimulation with LPS (100 ng/mL). Alternatively, following the addition of LPS, the PBMCs were left to incubate overnight at +37 °C before culture supernatants were removed and assessed for TNF-α by enzyme-linked immunosorbent assay (R&D Systems; Abingdon, UK) according to the manufacturer’s protocols.

#### 3.2.5. TNF-α Determination

The quantification of TNF-α in human serum was performed using a commercial ELISA kit (DuoSet) obtained from R&D Systems, Inc., and following the manufacturer’s instructions. Plate Preparation: First, R&D DuoSet ELISA 96-well microplates were coated with 4.0 μg/mL mouse antihuman TNF-R diluted in PBS overnight at room temperature. After washing, the plates were then blocked with PBS containing 1% BSA for a minimum of 1 h at room temperature and then washed. Assay Procedure: 100 μL of samples or standard was added and incubated for 2 h at room temperature. After washing (ELX406 Select, BIO-TEK), biotinylated anti-hTNF-α antibody was added and incubated at room temperature for 2 h, followed by incubation with streptavidin−peroxidase for 20 min. Detection of bound hTNF-α was carried out with 100 μL of substrate solution (H_2_O_2_ and tetramethylbenzidine), followed by measurement at 450 nm in a SPECTRA max Plus (Molecular Devices). These experiments were performed 2−3 times using the same experimental design. Duplicates from each series of experiments were averaged and expressed as hTNF-α levels in pg/mL.

#### 3.2.6. Study of Transport Through CaCo-2 Cell Barrier

To study the transport characteristics of a new NCE and account for passive or active transport, the CaCo-2 cells are used to assess permeability from the apical to basolateral side (AB transport) as well as basolateral to apical side (BA transport). Passage numbers from 20 to 40 are used for transport studies. Experiments are performed using monolayers grown with the Biocoat HTS-CaCo-2 Assay System (Becton & Dickinson) according to the protocol of the kit. A 24-well plate format from BIOCAT with PET membranes (1 μm pore size) and a layer of fibrillar collagen is also used for the experiments. Briefly, CaCo-2 cells are seeded at 500,000 cells/well in Mito+ Serum Extender-supplemented medium, incubated for 56 h. Then, the medium is changed to Mito+ supplemented Entero-STIM Differentiation Media (ESM+) and incubated for another 48 h. Then, ESM+ is renewed by fresh ESM+ and incubated for 24 h extra. Six days post-seeding cells are used to perform transport experiments. The NCE (solution in HBSS supplemented buffer and a maximum of 1% DMSO) or supplemented HBSS buffer (10 mM Hepes and 25 mM glucose at pH 7.4) will be placed in the apical or basolateral side as indicated in the table below and according to the AB or BA transport study. After a 3 h-incubation period, samples are collected, diluted with methanol (1:1), and analyzed by HPLC-UV. [^3^H]Mannitol at 115 nM is used as a positive control of the membrane integrity. The outcome of the assay obtained includes apparent permeability for directions (PappAB and PappBA), percentage of absorption, material balance after 3 h, and stability at 37 °C after 3 h.

#### 3.2.7. Metabolic Stability in Rat Microsomes

To determine metabolic stability, the test compound (n = 2 replicates) is mixed with liver microsomes at a final concentration of 5 µM, 1 mg/mL of microsomal protein, 50 mM potassium phosphate, 1 mM EDTA, and mM magnesium chloride at pH = 7.4 and at 37 °C on a shaker set at 900 rpm. The reaction is initiated by the addition of the NADPH-generating system (1 mM NADP+; 5 mM glucose-6-phosphate; 2.5 UI/mL glucose-6-phosphate dehydrogenase). Final percentage of organic solvent is 0.8% (0.79% ACN + 0.01% DMSO). The final incubation volume is 250 µL.

At 30 min, samples are processed by adding 250 µL of stop solvent (Acetonitrile) and centrifuged (4000 rpm, 10 min, room temperature). An aliquot of 60 mL of the supernatant is diluted with 60 µL of water, and the test compound concentration therein is measured by HPLC-MS/MS to determine the percentage of parent compound remaining compared to a blank (considered as 100% of parent remaining).

Testosterone is used as a reference control among different species

#### 3.2.8. Inhibition of LPS-Induced Neutrophilia in Mice po (SIGMA, Ref, L-2630)

Male, Balb/c mice (20–25 g) purchased from Charles River Laboratories. One week after acclimatation, the animals were randomly distributed into several treatment groups and dosed orally in a suspension with methyl cellulose 0.5% + Tween 0.1% in water. One hour later, animals were challenged by inhalation with LPS (*E. coli*, serotype O111:B4; Sigma Chemical Co., St Louis, MO, USA) at a concentration of 0.03 mg/mL in Phosphate saline Buffer.

Afterwards, at 3 h, animals were euthanised and surgically prepared for bronchial lavage by inserting a tracheal cannula. Animals (n = 6/group) were lavaged with 2 × 0.3 mL of phosphate-buffered saline, pH 7.2 (PBS). Bronchoalveolar lavage (BAL) fluid was analysed for neutrophil content.

#### 3.2.9. Oral PK in Rat

Male Wistar rats (225–250 g) purchased from Charles River Laboratories were fasted overnight before dose administration. For each compound, 3 rats were treated. Each compound was suspended in a mixture of 0.5% methylcellulose and 0.1% Tween 80 and administered by oral gavage (dose volume of 10 mL/kg). Blood samples were collected from the retro-orbital plexus in heparinized Eppendorfs at 0.25, 0.5, 1, 2, 3, 4, 6, and 24 h after dosing (n = 3 replicates). After centrifugation (4000 rpm, 10 min, room temperature) plasma is separated and stored at −20 °C until analysis. For analysis of the compound, plasma samples are thawed and centrifuged (4000 rpm, 10 min, room temperature). An aliquot of 30 mL of plasma is processed by adding 120 µL of stop solvent and centrifuged (4000 rpm, 10 min, room temperature). An aliquot of 50 µL of the supernatant is diluted with 50 µL of water and analysed by UPLC-MS/MS.

#### 3.2.10. Emesis in Ferret

Male castrated adult ferrets (1–2 kg) provided by Marshall Farms (North Rose, NY, USA) were used. They were housed in a humidity and temperature-controlled environment and fed on a carnivore diet (standard ferret food) with water “ad libitum”. Ferrets were fasted overnight. On the day of the experiment, ferrets (n = 6/group) were placed into individual observation cages. Following the oral administration of the drug or of its vehicle (5% *w*/*v* of methylcellulose with 0.1% *v*/*v* tween-80 in distilled water) they were observed continuously for a period of 3 h during which, the number of emetic episodes (i.e., rhythmic contraction of the abdomen with expulsion or attempt to expel solid/liquid matter from the gastrointestinal tract) were recorded in a timely manner. Oral administration of the drug or its vehicle was performed using a rubber feeding tube, size 12 Ch (Rüsch AG, Kernen, Germany).

#### 3.2.11. Ethics Statement

The animals were cared for in compliance with European Directive 2010/63/EU and Spanish and autonomous Catalan laws (Real Decreto 53/2013 and Decret 214/1997). Experimental procedures (DAMM5677 and DAMM4647, September 2011 and July 1998, respectively) were reviewed by the Animal Experimentation Ethics Committee of Almirall and approved by the appropriate authority (Catalan Government), following the ARRIVE guidelines.

All experiments performed on human samples were conducted in accordance with the Declaration of Helsinki. Human blood was obtained from human healthy donors from the Hospital de la Santa Creu i de Sant Pau (Barcelona) and approved by the Medical Ethics Committee of the Hospital (number EC/20/345/6135, 23 December 2020, Ethics committee of Hospital de la Santa Creu i de Sant Pau and EC/472011, 11 January 2012, Ethics committee of Hospital de la Santa Creu i de Sant Pau). Written informed consent was obtained from all patients.

### 3.3. Molecular Docking and Complex Potential Energy Minimization

The enzyme 3GWT (resolution 1.75Å) was chosen for our docking due to the similarity of the scaffold used for the co-crystallization, a quinoline [39,40]. The compounds were inserted on the catalytic site of the enzyme 3GWT by superimposing the conformation of the ligand co-crystallized with the enzyme (GSK-256066) [40].

All the 3D structures of the molecules were designed (“DS ViewerPro 6.0 Accelrys Software Inc.,” n.d.) [38].

For molecular docking, we used the AUTODOCK suite [37]. Docking studies were performed considering all amino acids within a radius of 2 nm (20 Å) from the binding site centre in vacuum and with water molecules present at the catalytic site (i.e., fourteen water molecules). For the potential energy minimization of the complexes, the ligand-enzyme was used with GROMACS [41], and the entire enzyme and the fourteen water molecules present in the catalytic site were considered. The partial atomic charge of the ligand structures was calculated with CHIMERA (CHIMERA 1.10.2; ACYPE Server (bio2 byte.be/acype/) using the AM1-BCC method, and the topology was created with ACPYPE based on the routine Antechamber.

For the potential energy calculation, the AMBER99sb force field parameters were applied. A conjugate gradient algorithm for energy minimization, with a tolerance of 10.0 kJ mol^−1^ nm^−1^ (the minimization is converged when the maximum force is smaller than this value), was used.

## 4. Conclusions

In this manuscript, we have reported an extensive study concerning the synthesis and the biological/pharmacological evaluation of several 5-acetyl-2-ethyl-6-phenylpyridazin-3(2H)-ones, variously substituted at position 4 with different aryl/alkylamines. To rationalize the biological values, we can state that the best results in terms of inhibitory potency on PDE4B1 were obtained for the 4-anilino derivatives (compounds of type **2**) and for the 4-(hetero)aryl amino of type **3**, with IC_50_ values in the low nanomolar range. By contrast, (cyclo)alkylamino-pyridazinones of type **4**, and products in which a linker has been inserted between the amine group bound at position 4 and the (hetero)aromatic ring (compounds of type **5**), are less active; similarly the 4-NH_2_ derivative 7, compound in which the NH group has been completely removed (compound **9**), converted in a tertiary amine (**5f**) or replaced with S or O (**10a** and **10b**), show low activity in the micromolar range or are completely inactive. These results clearly suggest that both the presence of the NH group and the (hetero)aromatic nucleus are important for activity and that these two portions must be directly connected. Regarding the insertion of substituents on the phenyl of aniline, these affect not only the potency on PDE4B1 differently, but also other isotypes, HARBS, and TNFα production.

Briefly summarizing the results of the two most interesting classes (compounds of type **2** and **3**), we can say that generally the introduction at the meta position of the unsubstituted **2a** of various groups or atoms, regardless of their nature and size, is advantageous for activity, leading to an increase of potency on PDE4B1 and maintaining good levels of inhibition of TNFα production. In this series, the most interesting is **2f** (R = CN), as for potency and selectivity, and overall, it strongly inhibits TNFα production (IC_50_ = 3.7 nM).

Moving the substituents to the para-position of the phenyl ring, a general decrease in activity is recorded (one/two orders of magnitude) with respect to the corresponding meta-isomers, and the most potent inhibitors of isotype PDE4B1 (**2n** and **2p**) show poor potency on TNFα production.

Finally, the o-substituted compounds **2j**, **2u,** and **2v** exhibit on the three isotypes and on HARBS IC_50_ values analogous to those of the meta isomers, but their ability to inhibit TNFα production is greatly diminished.

Moving on the 4-(hetero)aryl amino derivatives **3**, all these compounds, as pyrido-, quinoline- and naphthalene derivatives are provided with a potent inhibitory activity on PDE4s, with IC_50_ values in the low/subnanomolar range, but they exhibit low selectivity toward the three isoforms, and with the exception of compounds **3a**, **3b** and **3g**, a very good affinity for HARBS. The most interesting products as regards TNFα production are the amino-3-pyridyl derivatives **3a**, **3k**, the amino-5-quinolinyl **3f**, **3i**, **3j** and the naphthalene derivative **3l** (IC_50_ = 2.40–73.06 nM).

Further in vivo studies on the selected pyridylamino derivatives **3a** and **3k** indicate an acceptable metabolic stability and a good absorption profile for oral administration, but a shorter half-life and a low oral bioavailability due to high clearance. Instead, both compounds exhibit a low emetic effect, with MNED about 3–10-fold higher than Roflumilast. In particular, compound **3k** shows a favorable toxicological profile with no adverse events after 2 weeks of treatment (up to 10 mg/kg/day per os).

Finally, molecular modelling studies confirm our previous results about the correlation between water molecules in the active site and compounds’ activity.

For all compounds (as high and low potent), the main hydrogen bond interactions involve the oxygen of the 5-COCH_3_ moiety, the water molecules SOL1 and SOL13, by Zn and Mg ions, and the residue Gln443 of the enzyme, but, while the COCH_3_ oxygen of the low-active products form a high number of hydrogen bonds with SOL1 and SOL13, in the active compounds the COCH_3_ oxygen engages a significant number of hydrogen interactions with NH of the Gln443 of the target. These data suggest that the ability of molecules to form hydrogen bonds with the water molecules SOL1 and SOL13 in the active site is a discriminant for the potency, thus allowing distinction between more active and less active products.

## Figures and Tables

**Table 1:** Compounds of type **2**.

Comp. 2	R	Comp. 2	R
**a**	H	**k**	Cl (*p*)
**b**	F(*m*)	**l**	Br(*p*)
**c**	Cl(*m*)	**m**	OCH_3_(*p*)
**d**	Br(*m*)	**n**	COOH(*p*)
**e**	OCH_3_(*m*)	**o**	COOEt(*p*)
**f**	CN(*m*)	**p**	NO_2_(*p*)
**g**	CF_3_(*m*)	**q**	CN(*p*)
**h**	OH(*m*)	**r**	3,4-OCH_2_O
**i**	COOH(*m*)	**s**	3-Cl-4-OCH_3_
**j**	F(*o*)		

**Table 2:** Compounds of type **3**.

Comp. 3	Ar	Comp. 3	Ar
**a**	** 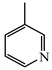 **	**f**	** 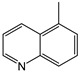 **
**b**	** 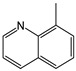 **	**g**	** 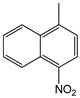 **
**c**	** 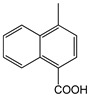 **	**h**	** 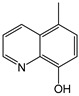 **
**d**	** 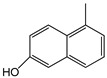 **	**i**	** 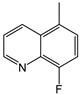 **
**e**	** 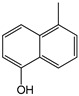 **		

**Table 3:** Compounds of type **4**.

Comp. 4	R
**a**	Et
**b**	iPr
**c**	cC_5_H_9_
**d**	cC_6_H_11_
**e**	cC_7_H_13_
**f**	C_6_H_13_
**g**	C_4_H_9_

**Table 4:** Compounds of type **5**.

Comp. 5	X	Ar
**a**	NHCH_2_	Ph-OCH_3_(*p*)
**b**	NHCH_2_	Ph-Cl(*p*)
**c**	NHCH_2_CH_2_	Ph-3,4-OCH_3_
**d**	NHCH_2_	Ph-COOH(*p*)
**e**	NHCH_2_	Ph-OCH_3_(*o*)
**f**	NCH_3_	Ph
**g**	NHCH_2_	3-pyridine
**h**	NHCH_2_	2-pyridine
**i**	NHCH_2_	4-pyridine
**j**	NHCH_2_CH_2_	2-pyridine
**k**	NHCH_2_	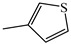
**l**	NHCH_2_	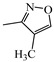

**Table 5:**
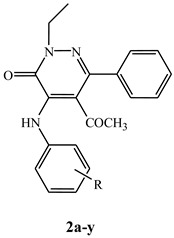
Inhibitory activity of final compounds of type **2**.

		Potency (IC_50_ nM)				
Comp. 2	R	PDE4B1 ^a^	PDE4A4 ^a^	PDE4D3 ^a^	HARBS ^a^	TNFα (hWB ^a^)
**a**	H	29.01 ± 1.25	4.70 ± 0.88	5.23 ± 0.91	73.31 ± 2.45	38.22 ± 1.84
**b**	F(*m*)	6.58 ± 0.92	1.31 ± 0.78	2.21 ± 0.65	17.12 ± 1.11	14.32 ± 1.08
**c**	Cl(*m*)	8.91 ± 0.89	2.71 ± 0.83	4.60 ± 0.78	95.31 ± 3.06	79.11 ± 1.56
**d**	Br(*m*)	27.01 ± 1.11	4.50 ± 0.68	6.12 ± 0.97	72.03 ± 1.87	130.02 ± 6.89
**e**	OCH_3_(*m*)	19.01 ± 0.84	5.92 ± 0.76	3.90 ± 0.69	16.12 ± 1.23	230.01 ± 7.87
**f**	CN(*m*)	0.73 ± 0.09	40.01 ± 1.87	17.00 ± 1.03	190.02 ± 9.89	3.7 ± 0.91
**g**	CF_3_(*m*)	229.21 ± 9.89	-	-	-	-
**h**	OH(*m*)	2998.01 ± 13.21	-	-	-	-
**i**	COOH(*m*)	907.02 ± 10.87	-	-	-	-
**t**	NO_2_(*m*)	7.20 ± 0.28	26.32 ± 0.09	18.03 ± 3.58	120.11 ± 3.87	150.03 ± 16.02
**j**	F(*o*)	7.38 ± 0.87	64.01 ± 5.89	37.12 ± 4.89	68.03 ± 5.33	150.11 ± 9.45
**u**	Cl(*o*)	5.32 ± 0.89	10.11 ± 0.87	5.92 ± 0.07	89.02 ± 6.58	480 ± 21.43
**v**	CH_3_O(*o*)	14.01 ± 1.37	18.03 ± 0.98	5.90 ± 0.27	190.03 ± 11.22	95.03 ± 1.23
**k**	Cl(*p*)	104.52 ± 4.21	-	-	-	-
**l**	Br(*p*)	124.03 ± 5.86	-	-	-	-
**m**	OCH_3_(*p*)	420.01 ± 8.83	-	-	-	-
**n**	COOH(*p*)	9.24 ± 0.65	19.11 ± 1.02	6.02 ± 0.09	52.04 ± 6.47	320.11 ± 5.11
**o**	COOEt(*p*)	6700.21 ± 21.11	-	-	-	-
**p**	NO_2_(*p*)	1.41 ± 0.07	14.21 ± 0.87	66.21 ± 3.25	920.07 ± 03.97	239.23 ± 12.11
**q**	CN(*p*)	20.06 ± 0.58	40.23 ± 4.88	55.32 ± 2.87	730.02 ± 12.09	61.03 ± 01.23
**r**	3,4-OCH_2_O	160.01 ± 10.21	-	-	-	-
**s**	3-Cl-4-OCH_3_	260.02 ± 12.87	-	-	-	-
**w**	3,5-F	3.11 ± 0.06	3.92 ± 0.23	4.70 ± 0.11	16.24 ± 2.07	17.01 ± 0.89
**Roflumilast**		0.69 ± 0.05	0.59 ± 0.06	0.27 ± 0.04	21.02 ± 0.85	36.01 ± 0.05
**Rolipram**	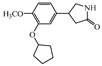	520 ± 5.05			7.03 ± 2.35	1683 ± 12.04

^a^ Values are the mean ± SD of three to four experiments performed in duplicate.

**Table 6:**
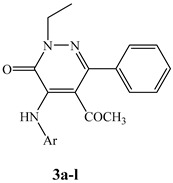
Inhibitory activity of final compounds of types **3**.

		Potency (IC_50_ nM)				
Comp. 3	Ar	PDE4B1 ^a^	PDE4A4 ^a^	PDE4D3 ^a^	HARBS ^a^	TNFα hWB ^a^
**a**	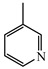	19.54 ± 1.43	14.02 ± 2.11	17.05 ± 1.88	150.02 ± 3.21	73.06 ± 1.12
**k**	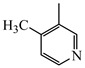	9.02 ± 0.05	11.04 ± 1.02	72.04 ± 1.88	24.23 ± 0.56	27.12 ± 3.44
**b**	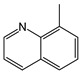	10.05 ± 1.22	13.02 ± 0.88	14.01 ± 1.08	130.12 ± 11.43	410.11 ± 9.88
**f**	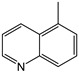	0.36 ± 0.05	26.03 ± 5.01	18.01 ± 0.84	5.10 ± 0.03	2.40 ± 0.16
**h**	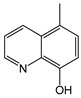	10.02 ± 2.04	13.12 ± 0.86	26.45 ± 1.88	39.02 ± 3.02	190.11 ± 11.76
**i**	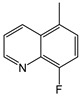	6.80 ± 0.07	5.91 ± 0.56	3.92 ± 0.07	36.08 ± 1.37	5.91 ± 0.08
**j**	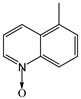	0.79 ± 0.02	0.45 ± 0.04	0.74 ± 0.06	6.60 ± 0.54	5.97 ± 0.07
**l**	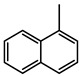	2.17 ± 0.03	1.8 ± 0.09	1.16 ± 0.02	17.01 ± 1.16	31.09 ± 2.88
**c**	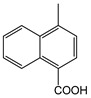	1.64 ± 0.08	3.92 ± 0.12	4.71 ± 0.24	30.11 ± 3.22	654.03 ± 11.22
**d**	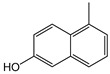	5.32 ± 1.47	23.11 ± 8.22	27.21 ± 2.56	9.00 ± 0.89	308.11 ± 7.65
**e**	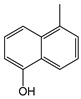	2.11 ± 0.08	n.d.	n.d.	5.60 ± 1.88	284.01 ± 9.58
**g**	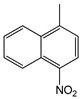	0.73 ± 0.02	40.12 ± 2.21	17.64 ± 1.43	120.32 ± 11.06	220.00 ± 8.11
**Roflumilast**		0.69 ± 0.05	0.59 ± 0.06	0.27 ± 0.04	21.02 ± 0.85	36.01 ± 0.05
**Rolipram**	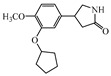	520 ± 5.05			7.03 ± 2.35	1683 ± 12.04

^a^ Values are the mean ± SD of three to four experiments performed in duplicate.

**Table 7:**
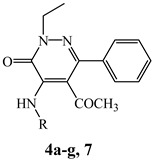
Inhibitory activity of the final compounds of types **4** and of compound **7**.

Comp.	R	PDE4B1 (IC_50_ nM) ^a^
**4a**	Et	974.51 ± 11.03
**4b**	iPr	387.54 ± 2.53
**4c**	cC_5_H_9_	370.01 ± 3.08
**4d**	cC_6_H_11_	360.02 ± 0.93
**4e**	cC_7_H_13_	430.04 ± 14.08

^a^ Values are the mean ± SD of three to four experiments performed in duplicate.

**Table 8:**
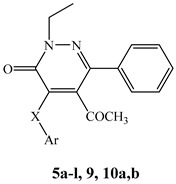
Inhibitory activity of final compounds **5a–l**, **9** and **10**.

Comp.	X	Ar	PDE4B1 (IC_50_ nM) ^a^
**5a**	NHCH_2_	Ph-OCH_3_(*p*)	1400.02 ± 7.83
**5b**	NHCH_2_	Ph-Cl(*p*)	2542.5 ± 21.05
**5c**	NHCH_2_CH_2_	Ph-3,4-OCH_3_	34.4% (10 μM)
**5d**	NHCH_2_	Ph-COOH(*p*)	5200.32 ± 8.42
**5e**	NHCH_2_	Ph-OCH_3_(*o*)	1500.43 ± 2.87
**5f**	NCH_3_	Ph	1258.11 ± 21.06
**5g**	NHCH_2_	3-pyridine	113.04 ± 7.03
**5h**	NHCH_2_	2-pyridine	181.23 ± 5.04
**5i**	NHCH_2_	4-pyridine	82.51 ± 6.08
**5j**	NHCH_2_CH_2_	2-pyridine	1200.03 ± 14.06
**5k**	NHCH_2_	3-thienyle	190.63 ± 16.09
**5l**	NHCH_2_	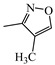	2400.24 ± 10.03
**9**	/	Ph-F(m)	58% (10 μM)
**10a**	S	Ph	2404.11 ± 3.08
**10b**	O	Ph	29% (10 μM)

^a^ Values are the mean ± SD of three to four experiments performed in duplicate.

**Table 9:** In vitro profile of compounds **3a** and **3k** compared with Roflumilast.

	3a	3k	Roflumilast
**IC_50_ PDE4A4** (nM)	14.02 ± 2.11	11.04 ± 1.02	0.59 ± 0.06
**IC_50_ PDE4B1** (nM)	19.54 ± 1.43	9.02 ± 0.05	0.69 ± 0.05
**IC_50_ PDE4D3** (nM)	17.05 ± 1.88	72.04 ± 1.88	0.27 ± 0.04
**TNFα hWB** (nM)	73.06 ± 1.12	27.12 ± 3.44	36.01 ± 0.05
**TNFα hPBMC** (nM)	19.08 ± 1.12	9.01 ± 0.12	2.60 ± 0.13
**CaCo-2** (10^−6^ cm/s)	39	45	39

**Table 10:** In vivo profile and PK p.o. parameters of compounds **3a** and **3k** compared with **Roflumilast**.

	3a	3k	Roflumilast
**Inhibition of LPS-induced neutrophilia in mice** (ED_50_ = mg/kg p.o., n = 6)	8.31	8.80	2.01
**Rat metabolism** %	92	97	73
**Human metabolism** %	33	52	39
**Oral PK in rat** (10 mg/kg, n = 3)	C_max_ = 265 ng/mL AUC = 390 ng.h/mL	C_max_ = 389 ng/mL t_max_ = 0.3 h t_1/2_ = 1.3 AUC = 1271 ng.h/mL	C_max_ = 56 ng/mL t_max_ = 4.4 h t_1/2_ = 4.2 AUC = 95 ng.h/mL
**Emesis in ferret p.o. (MNED ^a^, mg/kg, n = 6)**	2.70	1.10	0.30

^a^ MNED = Maximal Not Emetic Dose.

**Table 11:** Active ligands: H-Bond, distance (Å) between COCH_3_ oxygen and Gln443, SOL1 e SOL13.

Compound	Pose	GLN443		SOL1		SOL13	
**2c**	1	1.99	3.49				
	2	2.46	3.09				
	3						
**2e**	1	1.91					
	2						
**2f**	1	1.99	3.47				
	2						
	3						
**2n**	1	1.89	3.44				
	2	3.4	3.36				
**2p**	1	2.11	3.43				
	2			1.79	2.97	2.17	3.15
**3a**	1			1.8	2.95	2.26	3.03
	2					2.04	
**3k**	1			1.83	3.06	1.89	3.15
	2						

**Table 12:** Active ligands: H-Bond, distance (Å) between COCH_3_ oxygen and Gln443, SOL1 and SOL13.

Compound	Pose	GLN 443		SOL1		SOL13	
**2h**	1	2.13	3.41				
	2			1.82	3.02	1.92	3.14
	3	2.64	3.34				
**2i**	1	1.96	3.43				
	2						
**2o**	1	2.1	3.48				
	2						
**4f**	1			1.79	3	1.85	3.12
	2						
**5d**	1			1.93	3.24	1.78	3.21
	2						
**7**	1			1.76	3.01	1.84	3.08
**10a**	1			1.87	3.12	1.79	3.13
	2						

## Data Availability

The original contributions presented in this study are included in the article/Supplementary Material. Further inquiries can be directed to the corresponding author(s).

## Supplementary Materials

The following supporting information can be downloaded at: https://www.mdpi.com/article/10.3390/molecules31040699/s1. Table of contents: Chemistry (description of intermediate **8**); ^1^H-NMR spectra of some representative compounds; Molecular modeling studies (Tables S1–S5); Elemental analyses (Table S6); Figures S1–S4; References.
