# Supplementary material for: Discovery of Potent PDE4 Inhibitors with 3(2H)-Pyridazinone Scaffold: Synthesis, In Silico Studies and In Vitro/Vivo Evaluation
Source: Molecules. 2026 Feb 17;31(4):699. doi: 10.3390/molecules31040699 (PMC12943719; doi:10.3390/molecules31040699)
Supplement: Supplementary file 1 [file molecules-31-00699-s001.zip › molecules-4074279-supplementary.pdf]

Supplementary materials

for

Discovery of Potent PDE4 Inhibitors with 3(2H)-Pyridazinone Scaffold: Synthesis, in Silico Studies and In Vitro/Vivo Evaluation

Claudia Vergelli [1,*], Letizia Crocetti [1], Gabriella Guerrini [1], Fabrizio Melani [1], Jordi Gracia [2], Maria Antonia Buil [3], Yolanda Garrido [3], Lluis Pagès [3], Joan Taltavull [3], Amadeu Gavaldà [4], Elena Calama [4] and Maria Paola Giovannoni [1]

[1]NEUROFARBA, Pharmaceutical and Nutraceutical Section, University of Florence, Via Ugo Schiff 6, 50019 Sesto Fiorentino, Italy.
[2]Medicinal Chemistry Department, Evotec (France) SAS, Campus Curie, 195, route d'Espagne, 31036 Toulouse CEDEX, France
[3]New Chemical Entities Discovery & Early Development, Almirall R&D, carrer Laureà Miró 408-410, 08980 Sant Feliu de Llobregat, Spain
[4]Pharmacology, Almirall R&D, carrer Laureà Miró 408-410, 08980 Sant Feliu de Llobregat, Spain

* To whom correspondence should be addressed
Claudia Vergelli
Dipartimento NEUROFARBA, Sezione Farmaceutica e Nutraceutica
Via Ugo Schiff 6
Sesto Fiorentino 50019 Firenze
Tel +39-055-4573790
Fax +39-055-4573780
E-mail: claudia.vergelli@unifi.it

Table of contents

1. **Chemistry** (description of intermediate **8**)
2. **^{1}H-NMR spectra of some representative compounds**
3. **Molecular modeling studies (Tables S1-S5, Figures S1-S4)**
4. **Elemental analyses (Table S6)**
5. **References**

1. Chemistry

Reagents and starting materials were obtained from commercial sources. Extracts were dried over Na2SO4, and the solvents were removed under reduced pressure. All reactions were monitored by thin layer chromatography (TLC) using commercial plates precoated with Merck silica gel 60 F-254. Visualization was performed by UV fluorescence (λmax = 254 nm) or by staining with iodine or potassium permanganate. Chromatographic separations were performed on a silica gel column by gravity chromatography (Kieselgel 40, 0.063-0.200 mm; Merck), flash chromatography (Kieselgel 40, 0.040-0.063 mm; Merck. Yields refer to chromatographically and spectroscopically pure compounds, unless otherwise stated. Compounds were named following IUPAC rules, as applied by Beilstein-Institut AutoNom 2000 (4.01.305) or CA Index Name. All melting points were determined on a microscope hot stage Büchi apparatus and are uncorrected. ^{1}H NMR spectra were recorded with Avance 400 instruments (Bruker Biospin Version 002 with SGU). Chemical shifts (δ) are reported in ppm to the nearest 0.01 ppm using solvent as the internal standard. Coupling constants (J values) are given in Hz and were calculated using 'TopSpin 1.3' software rounded to the nearest 0.1 Hz. Mass spectra (m/z) were recorded on an ESI-TOF mass spectrometer (Bruker Micro TOF), and reported mass values are within the error limits of ± 5 ppm mass units.

5-Acetyl-4-chloro-2-ethyl-6-phenylpyridazin-3(2H)-one, 8

A mixture of 5-acetyl-2-ethyl-4-nitro-6-phenylpyridazin-3(2H)-one **1** (0.49 mmol) [1] and 6N HCl (5.6 mL) in acetone (3 mL) was stirred at 100 °C for 5 h. After cooling, cold water was added and the suspension was extracted with CH_2Cl_2 (3 x 15 mL). Evaporation of the solvent afforded compound **8**. Yield = 98%; oil; ^{1}H-NMR ($CDCl_3$) δ 1.40 (t, 3H, $CH_2\mathit{CH_3}$, J = 7.2 Hz); 2.18 (s, 3H, $COCH_3$); 4.28 (q, 2H, $\mathit{CH_2}CH_3$, J = 7.2 Hz); 7.38 (s, 5H, Ar). ESI-MS calcd. for $C_{14}H_{13}ClN_2O_2$, 276.72; found: *m/z* 277.43 $[M+H]^+$.

2. ^{1}H-NMR spectra of some representative compounds

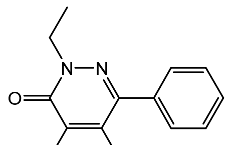

1H NMR Compound 2a
CDC13

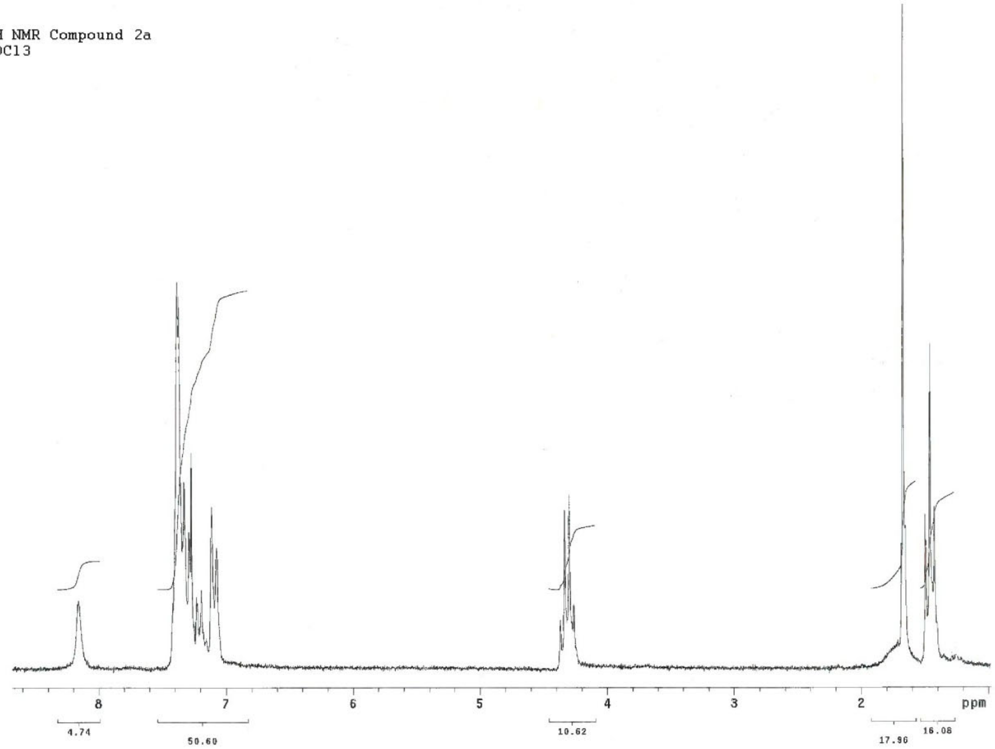

1H-NMR Compound 2b
CDC13

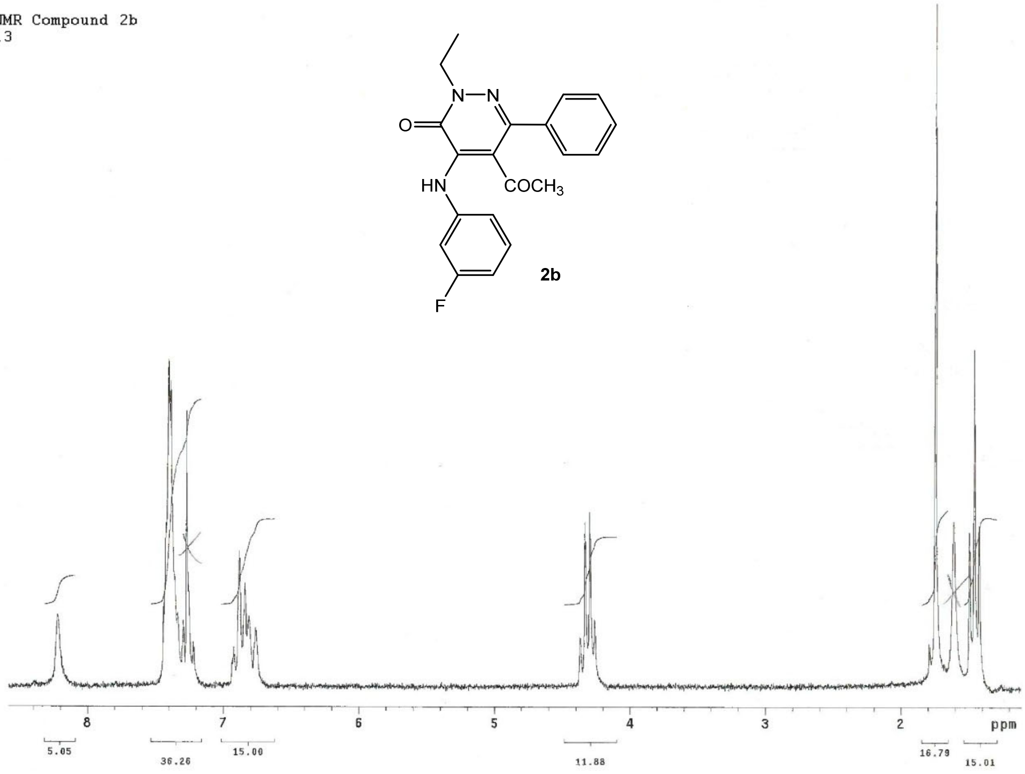

1H-NMR Compound 2h
CDCl3

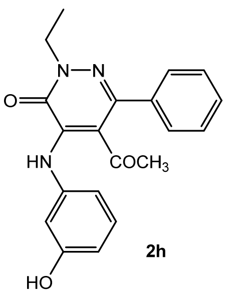

2h

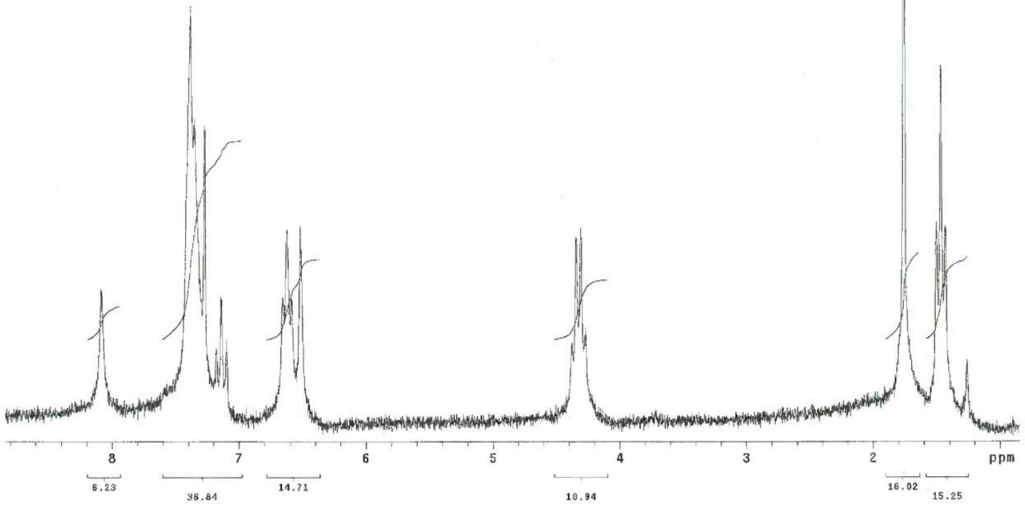

1H-NMR Compound 2i
CDCl3

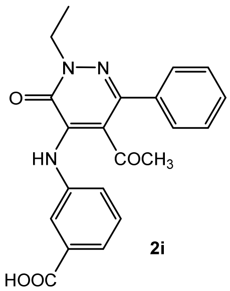

2i

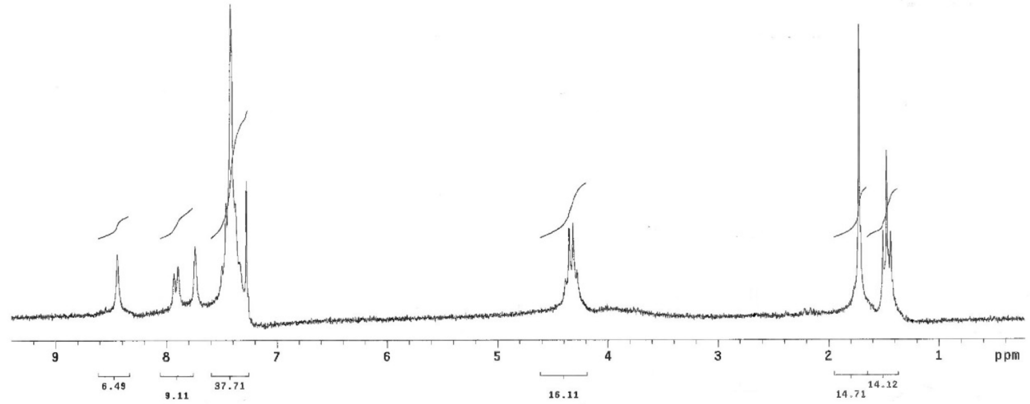

1H-NMR Compound 2n
DMSO

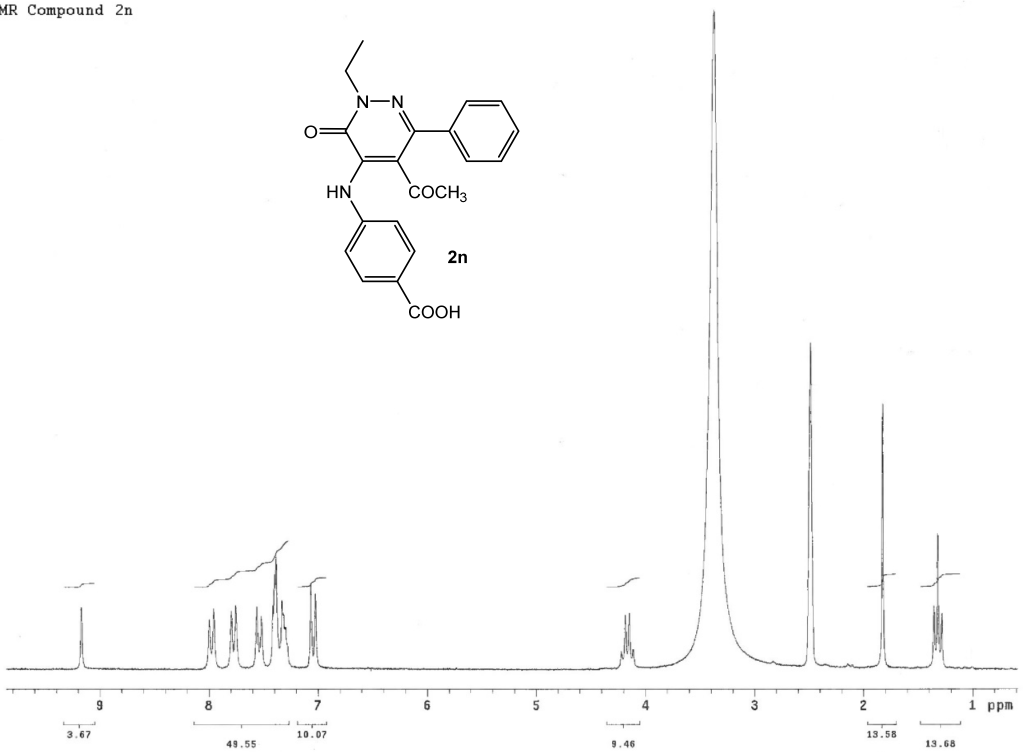

1H-NMR Compound 2n
DMSO + D2O

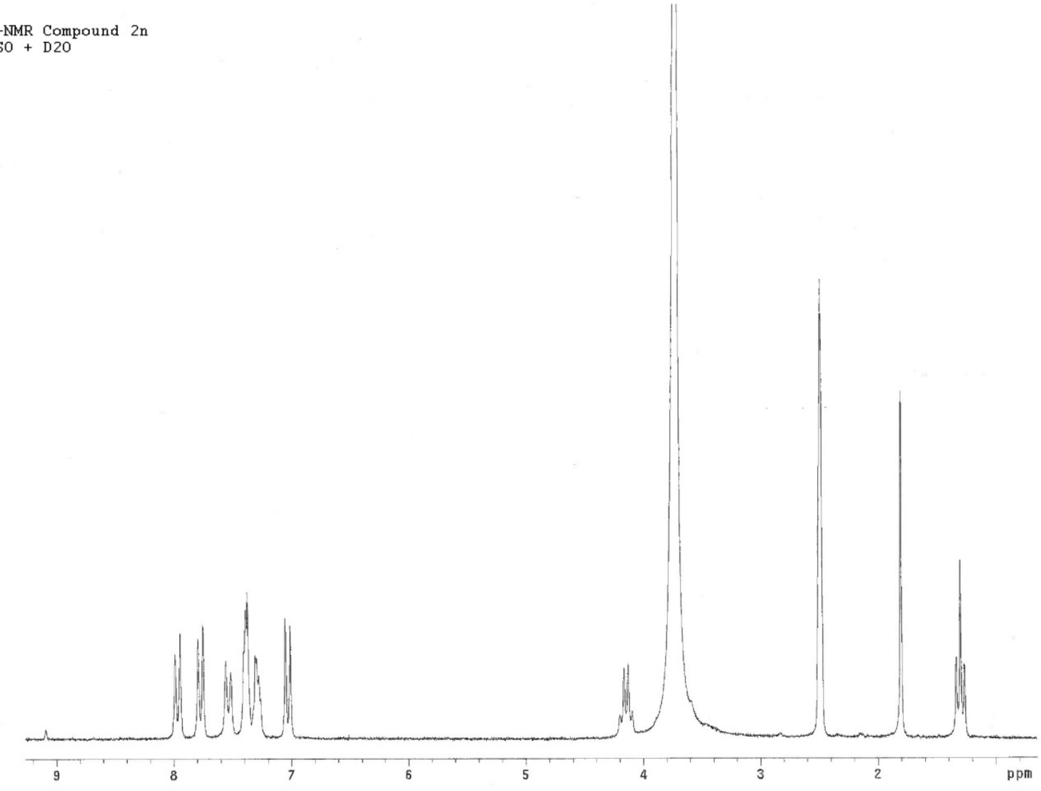

1H-NMR Compound 2o
CDCl3

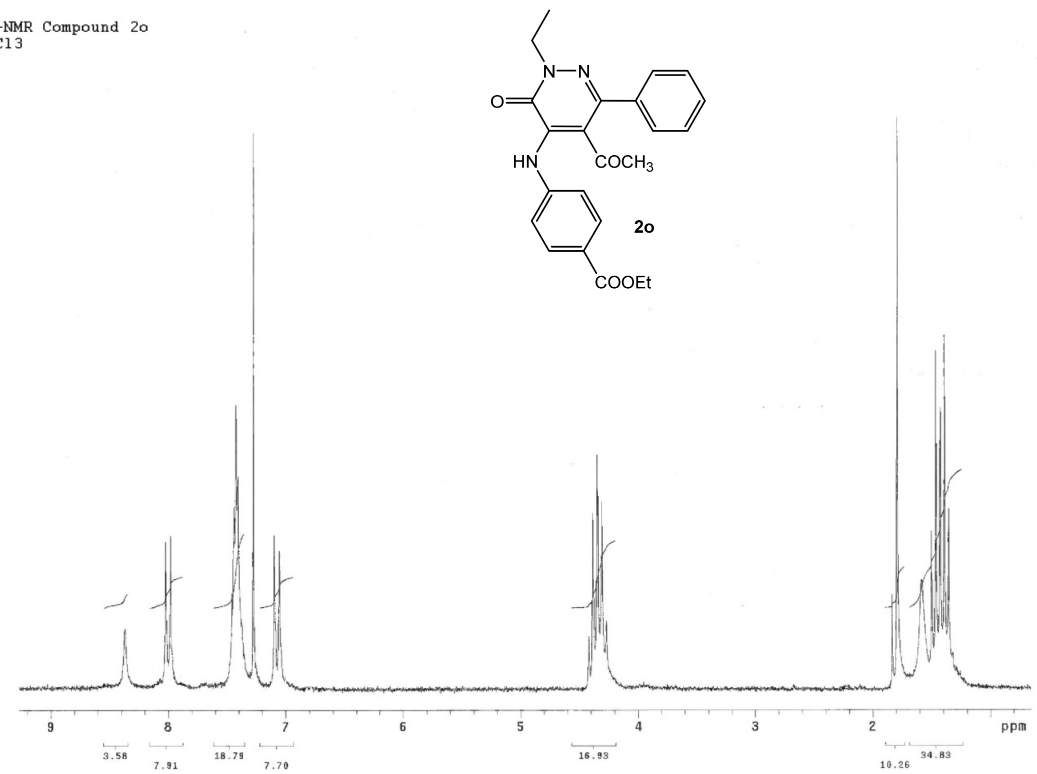

1H NMR Compound
4c
CDCl3

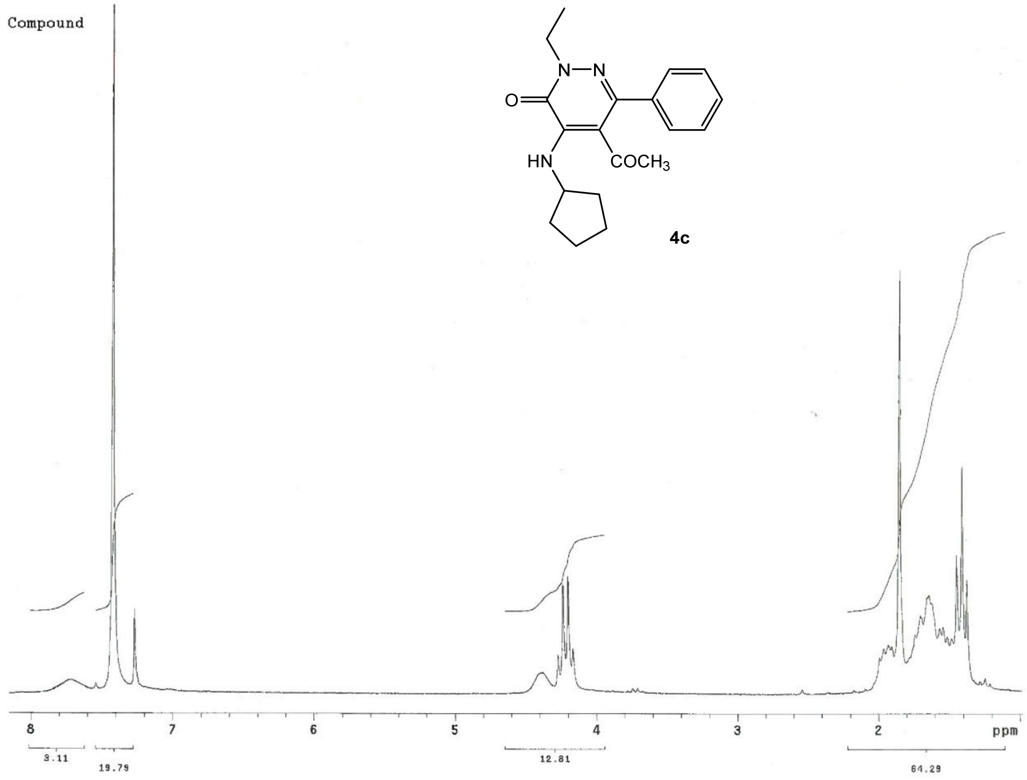

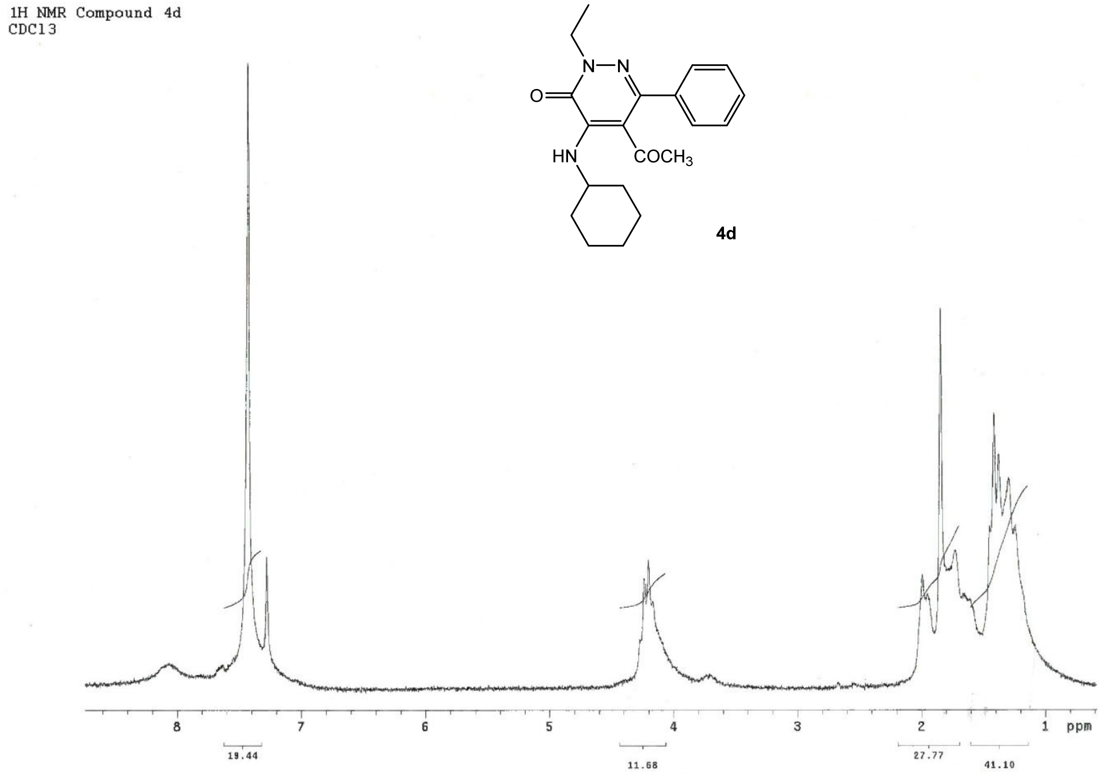

1H NMR Compound 4d
CDCl3
O
N
N
HN
COCH3
4d
8
7
6
5
4
3
2
1
ppm
19.44
11.68
27.77
41.10

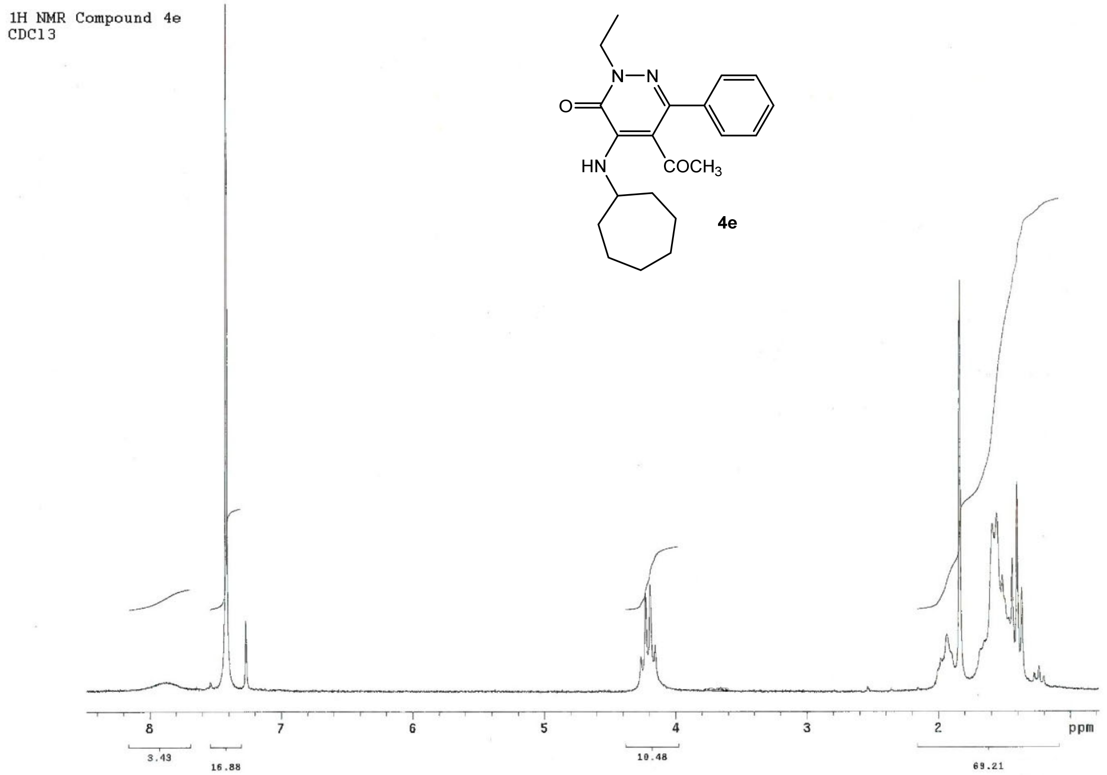

1H NMR Compound 4e
CDCl3
O
N
N
HN
COCH3
4e
8
7
6
5
4
3
2
ppm
3.43
16.88
10.48
69.21

1H NMR Compound 4f
CDCl3

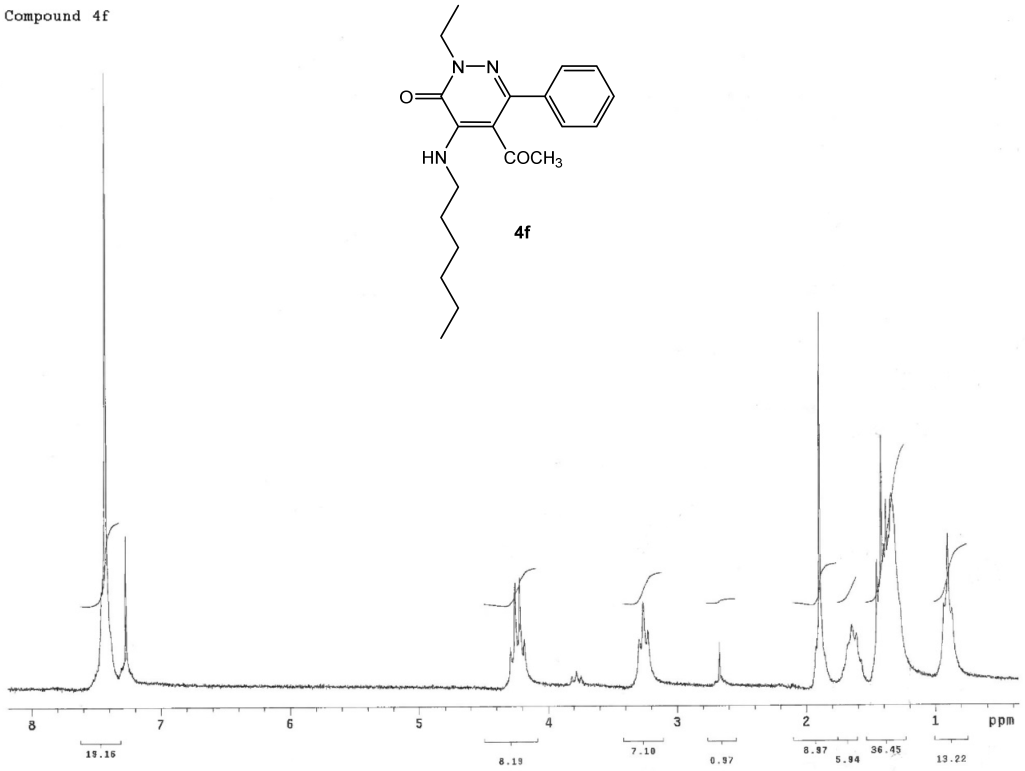

1H NMR
Compound 4g
CDCl3

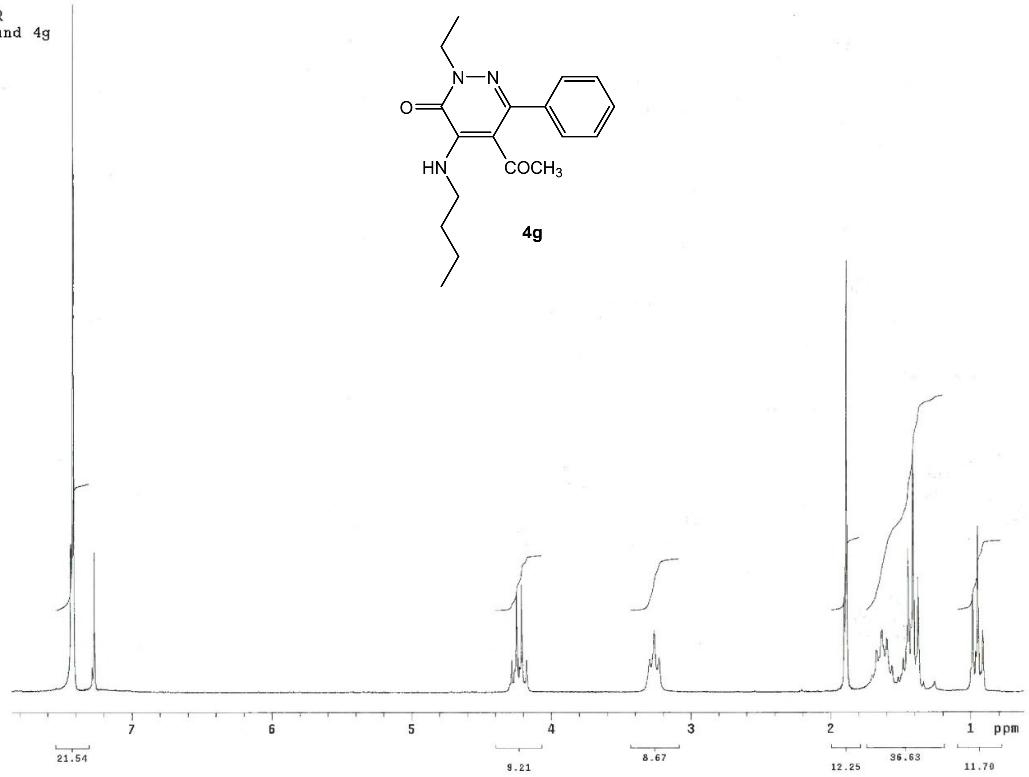

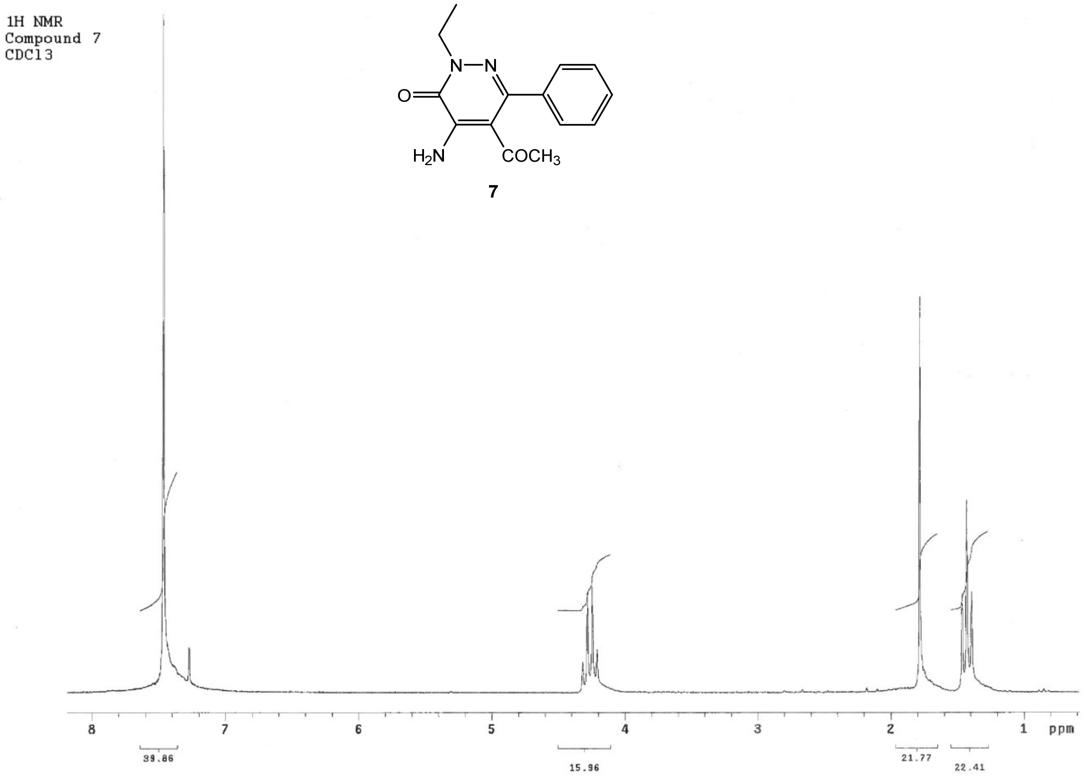
1H NMR
Compound 7
CDCl3
O
N
N
H2N
COCH3
7
8
7
6
5
4
3
2
1
ppm
39.86
15.96
21.77
22.41

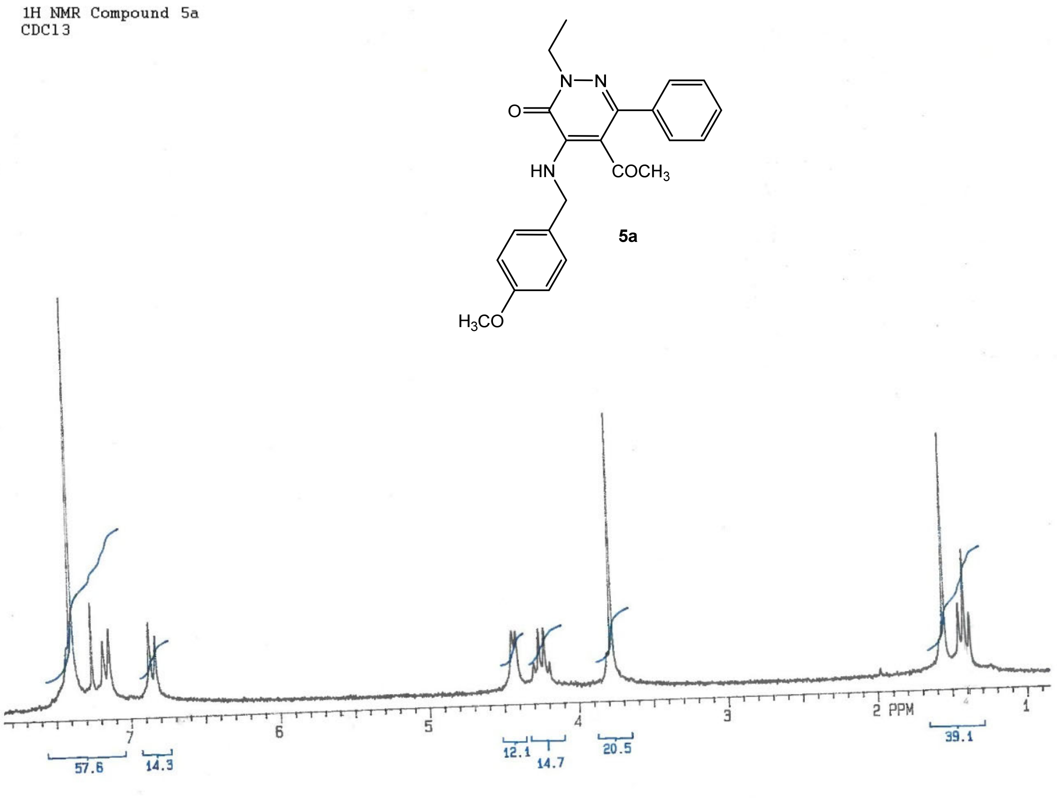
1H NMR Compound 5a
CDCl3
O
N
N
HN
COCH3
5a
H3CO
7
6
5
4
3
2 PPM
1
57.6
14.3
12.1
14.7
20.5
39.1

1H NMR Compound 5b
CDC13

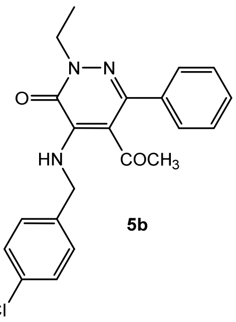

5b

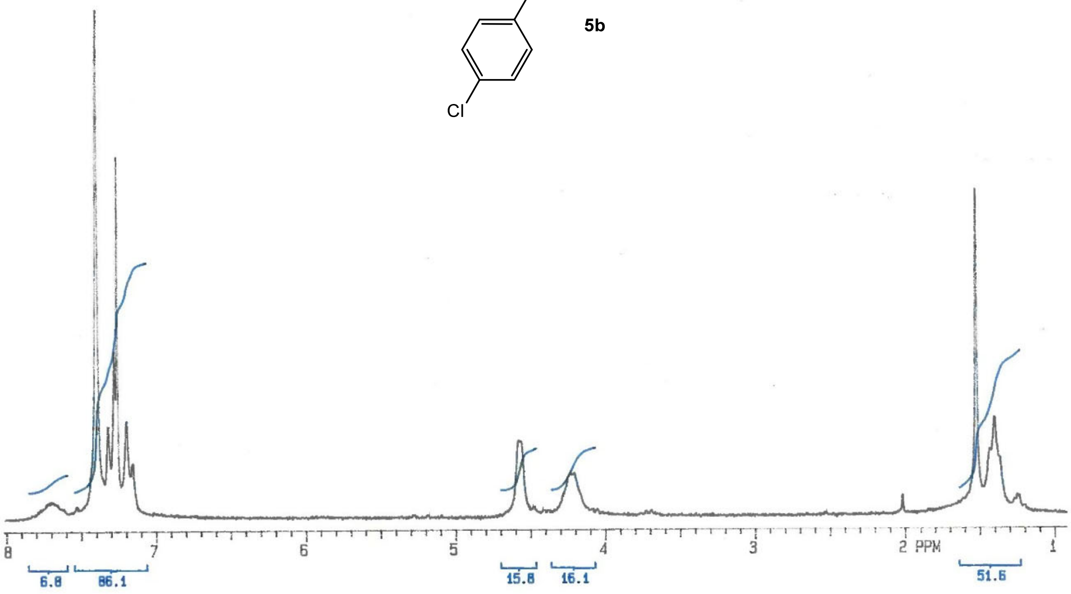

1H NMR Compound 5c
CDC13

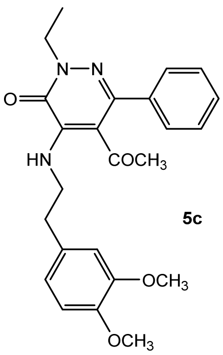

5c

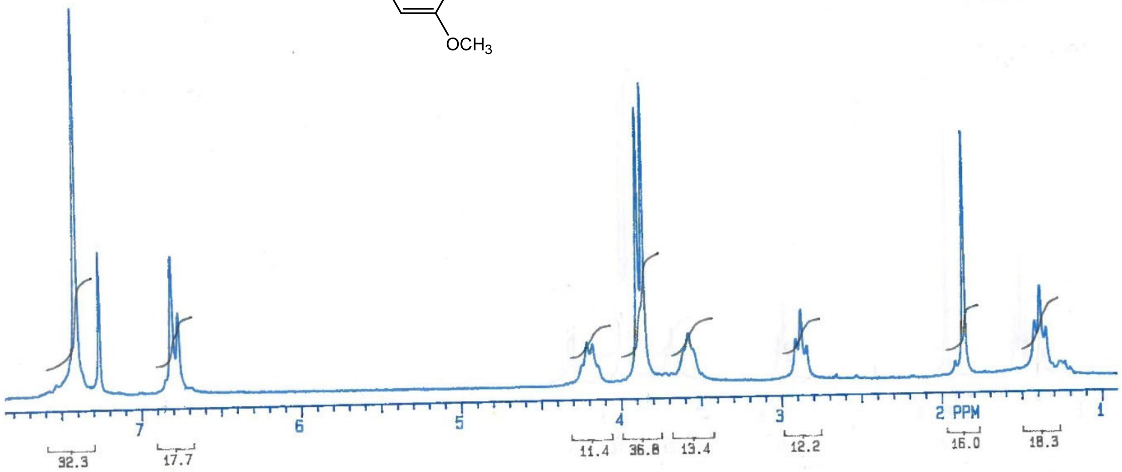

1H NMR Compound 5d
CDCl3

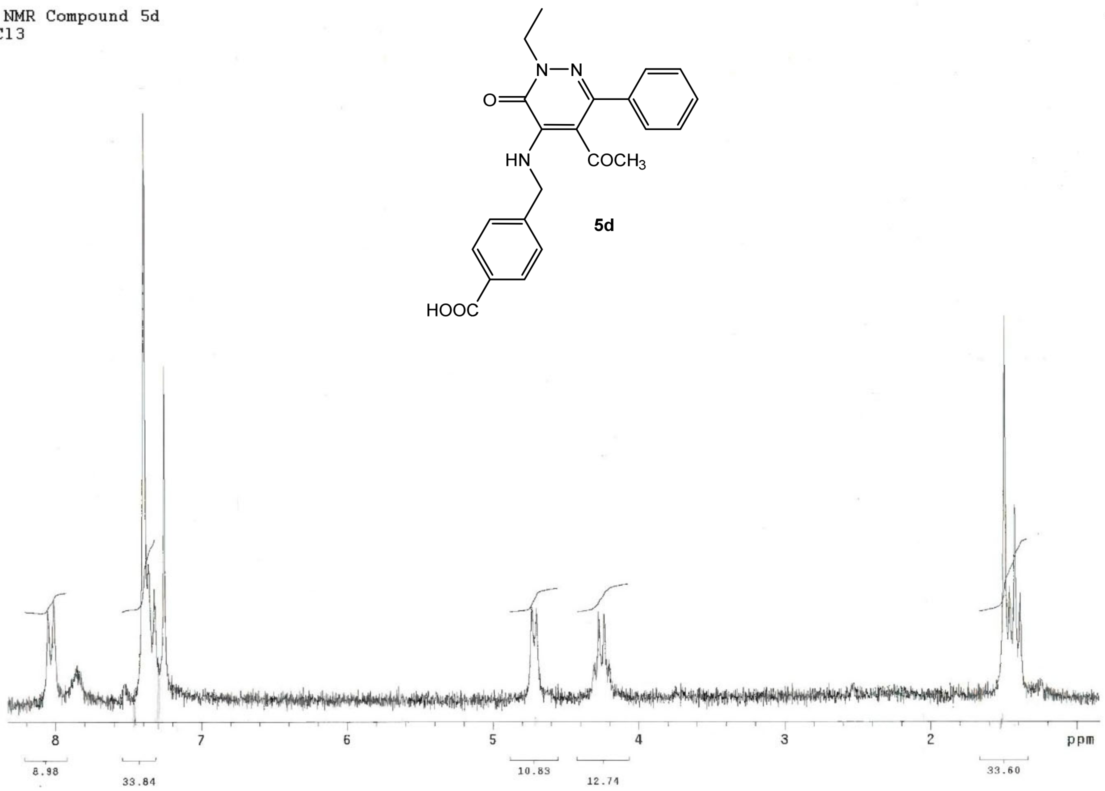

1H NMR Compound 5d
CDCl3 + D2O

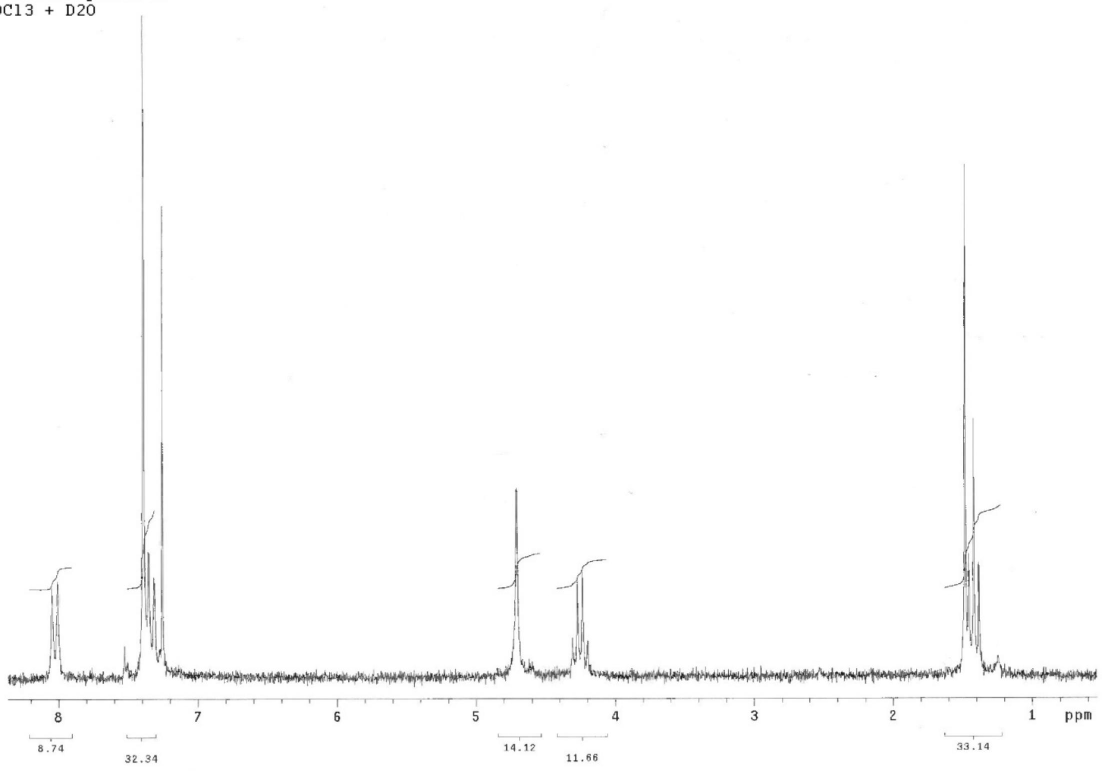

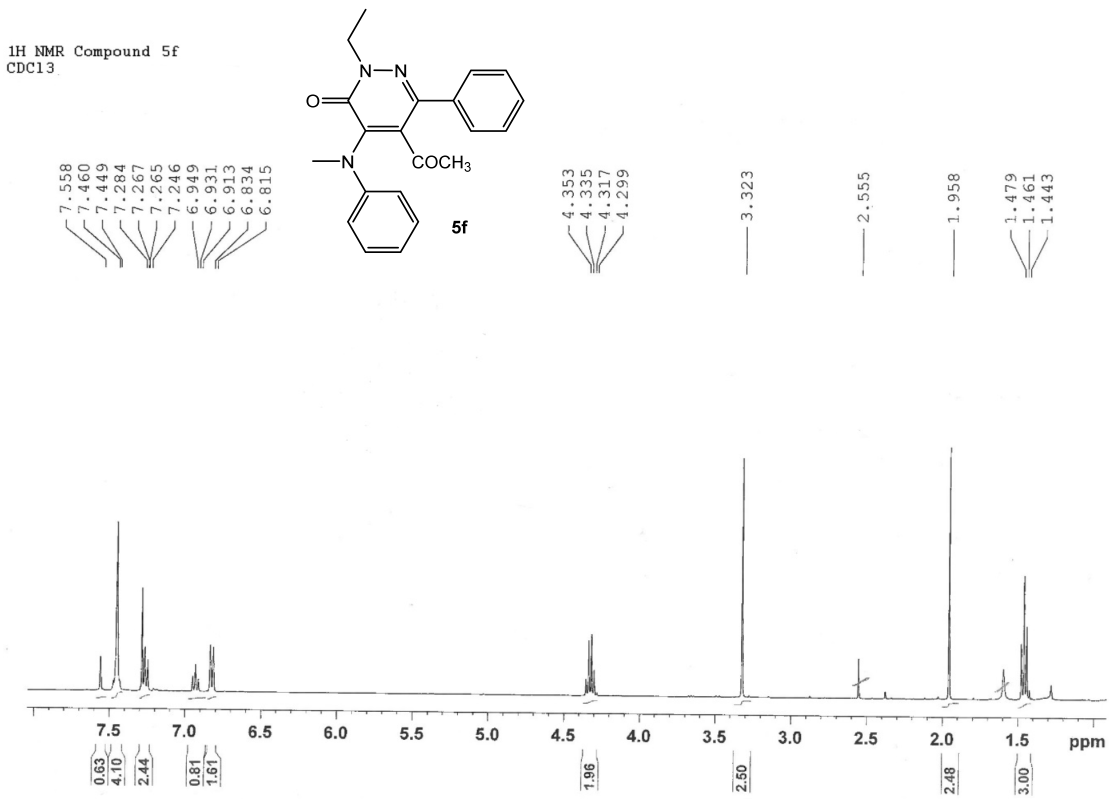

1H NMR Compound 5f
CDCl3
5f
COCH3
7.558 7.460 7.449 7.284 7.267 7.265 7.246 6.949 6.931 6.913 6.834 6.815
4.353 4.335 4.317 4.299
3.323
2.555
1.958
1.479 1.461 1.443
7.5 7.0 6.5 6.0 5.5 5.0 4.5 4.0 3.5 3.0 2.5 2.0 1.5 ppm
0.63 4.10 2.44 0.81 1.61 1.96 2.50 2.48 3.00

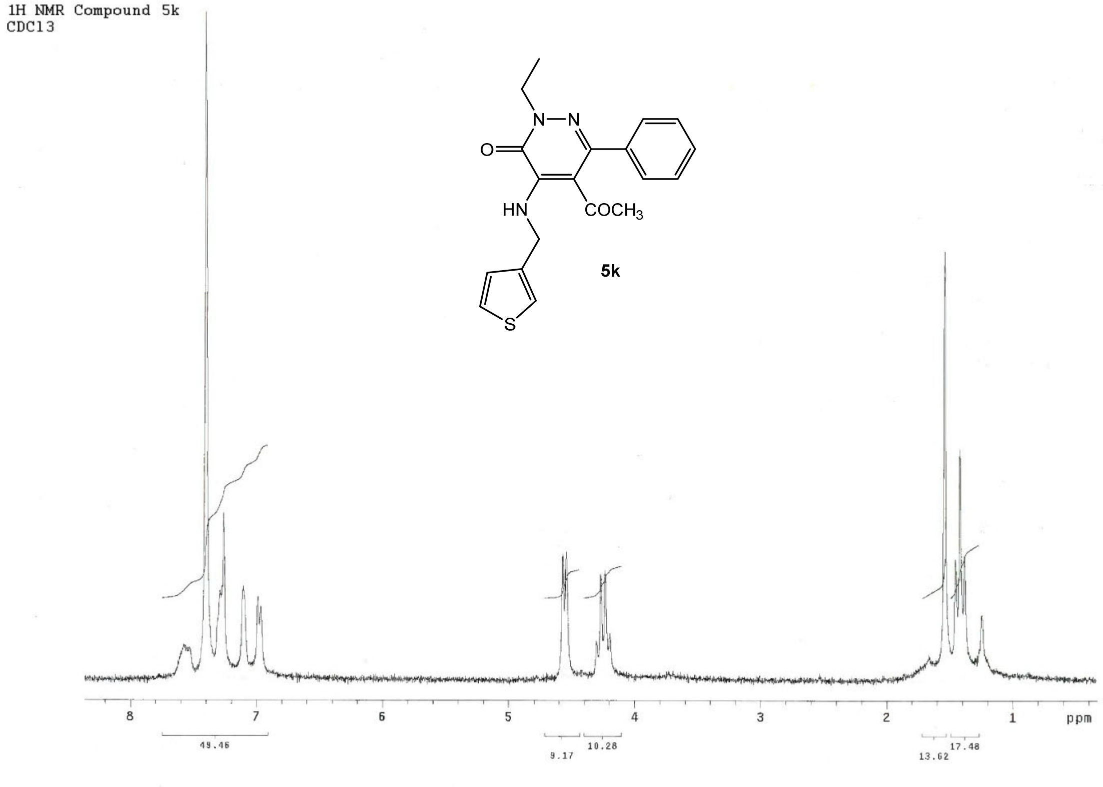

1H NMR Compound 5k
CDCl3
5k
HN
COCH3
S
8 7 6 5 4 3 2 1 ppm
48.46
9.17 10.28
13.62 17.48

1H NMR Compound 5k
CDCl3 + D2O

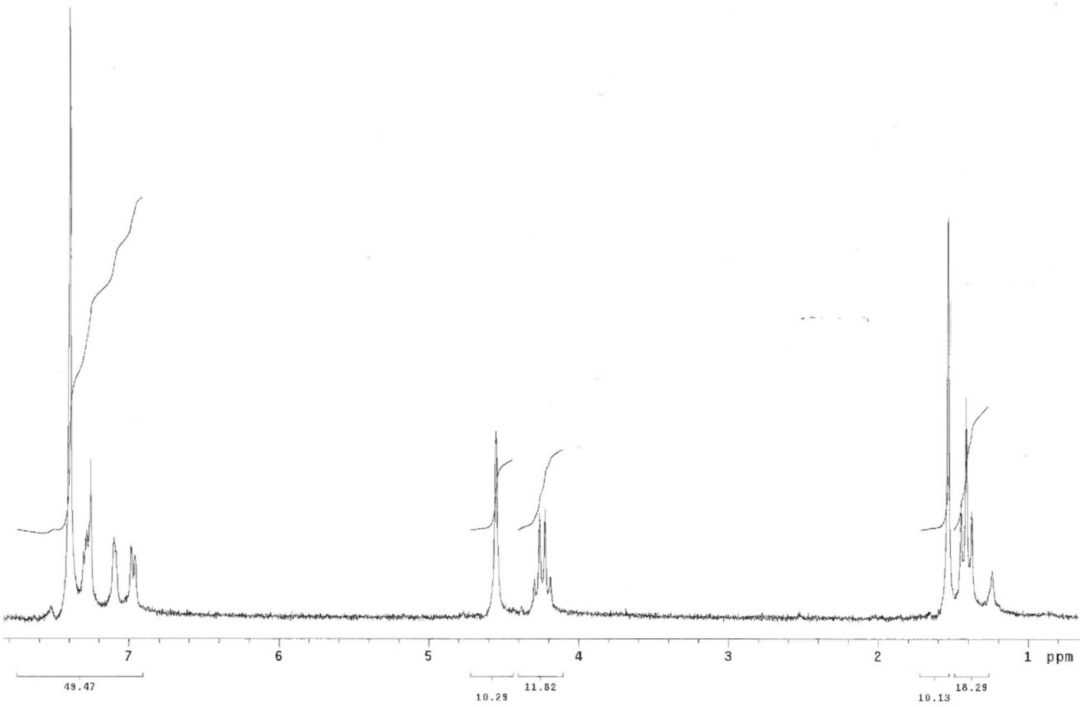

1H NMR Compound 5l
CDCl3

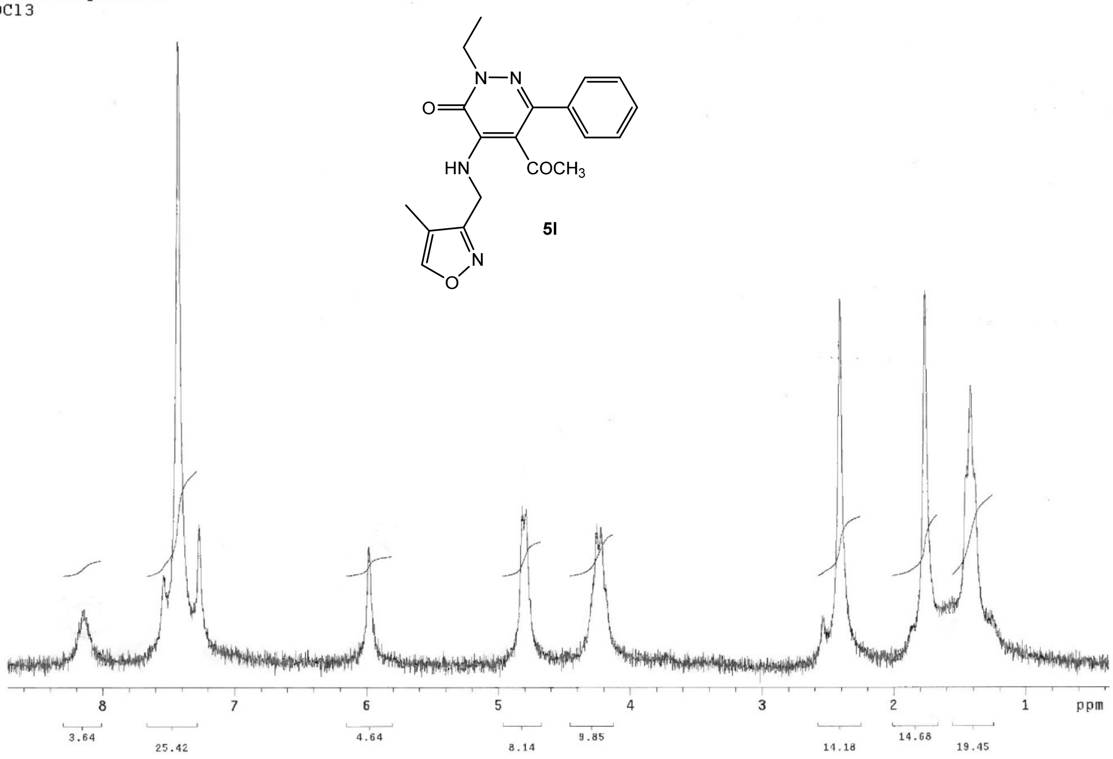

1H NMR Compound 51
CDC13 + D2O

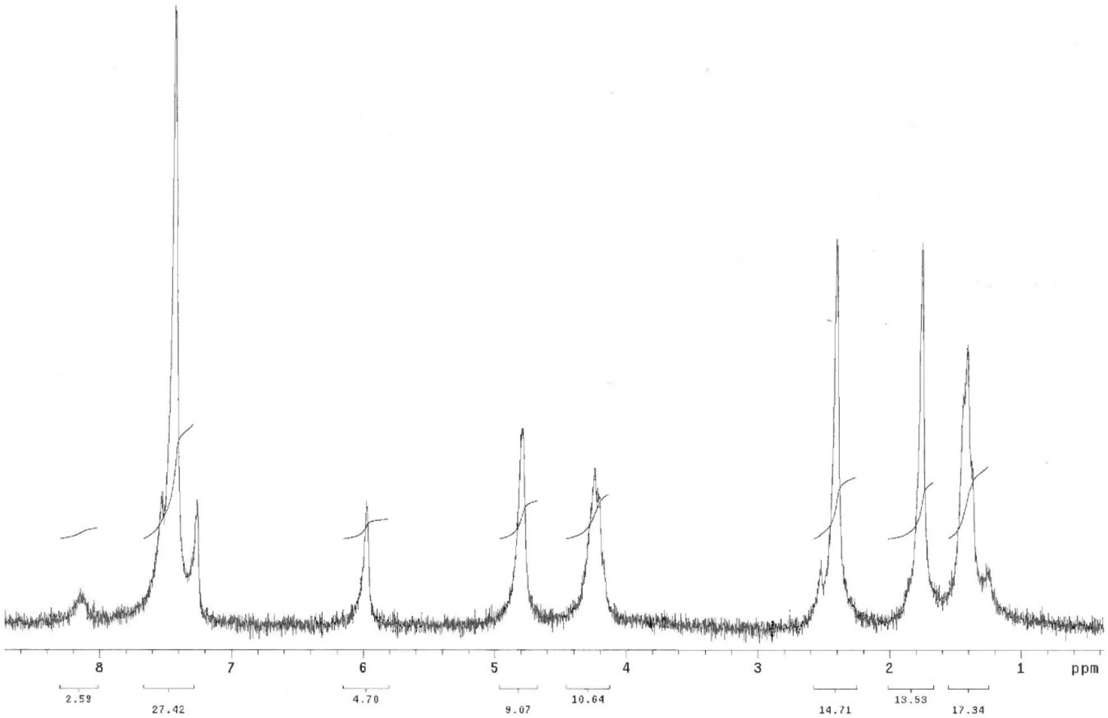

1H NMR Compound 9
CDC13

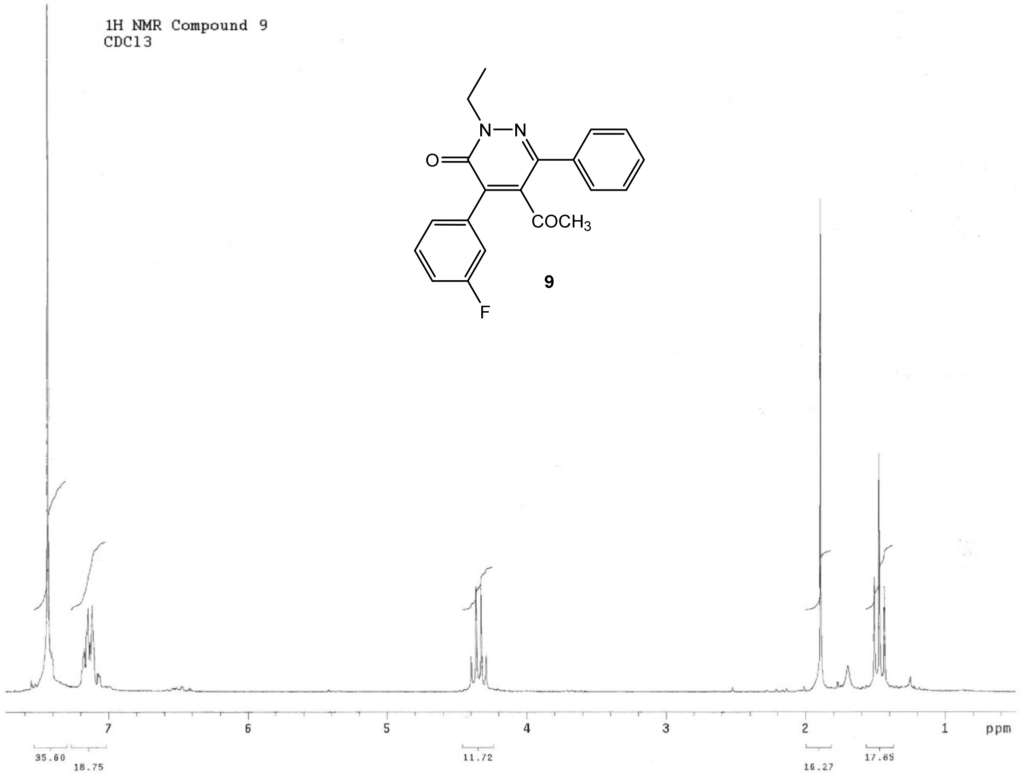

1H NMR Compound 10a
CDCl3

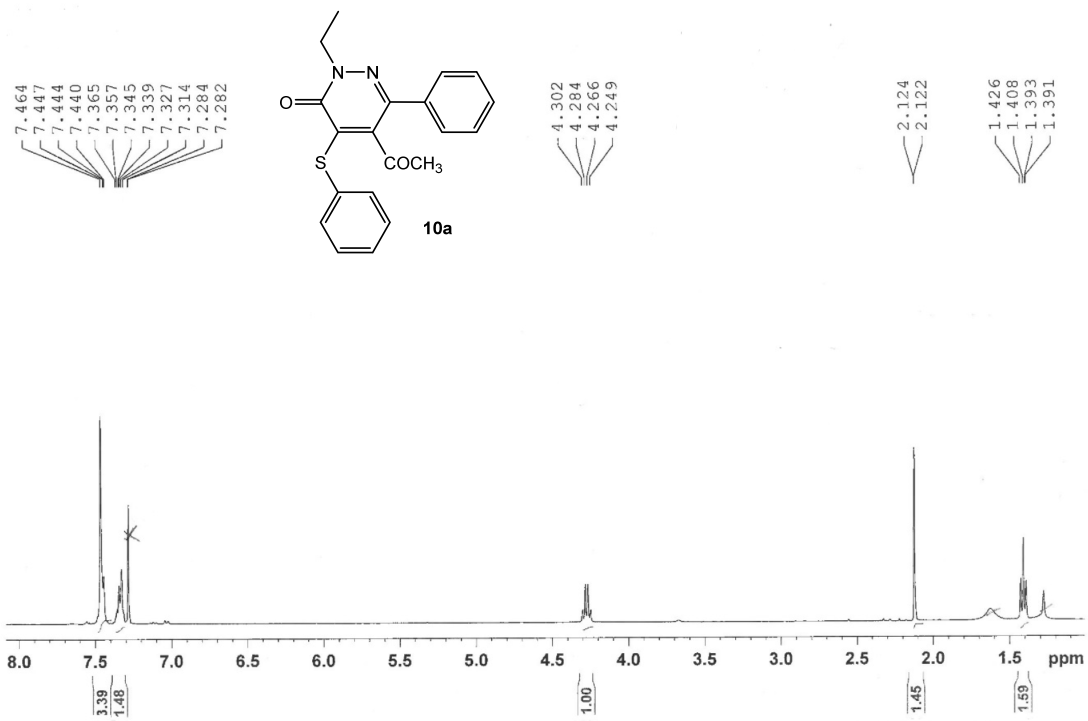

1H NMR Compound 10b
CDCl3

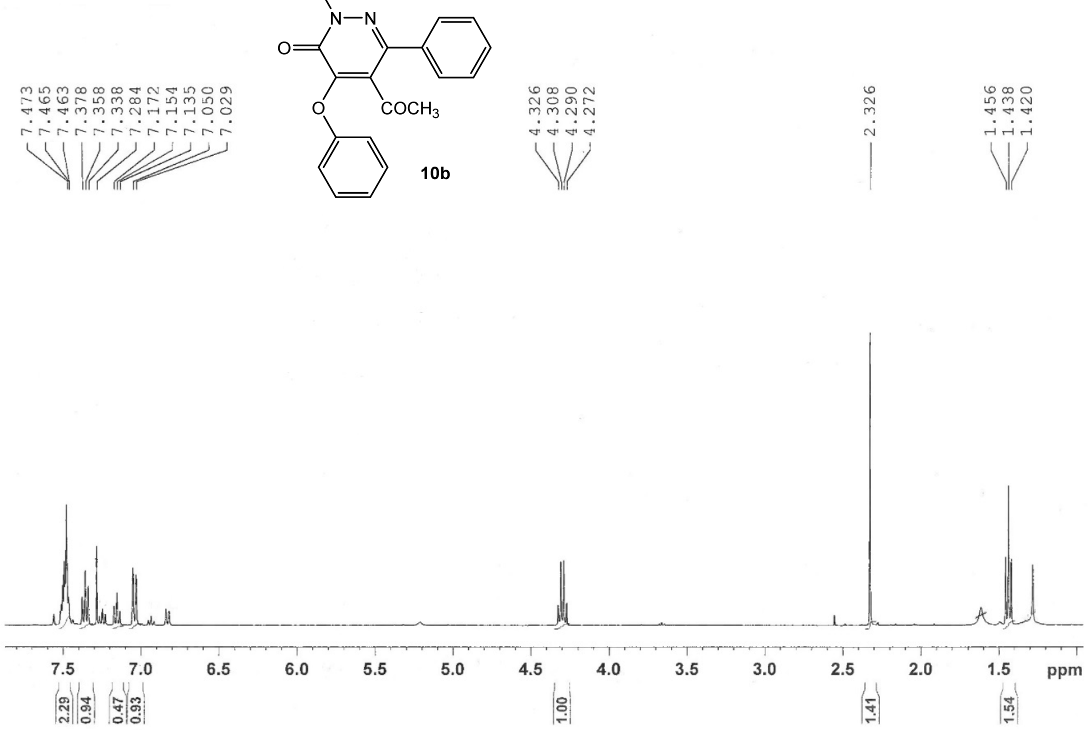

3. **Molecular Modelling studies**

Table S1. Number of poses for each ligand in enzyme in vacuum and with water

**Compound number**	**Poses in enzyme in vacuum**	**Poses in enzyme with 14 mol water**
**2c**	3	3
**2e**	3	2
**2f**	3	3
**2n**	3	2
**2p**	3	2
**3a (A)**	3	2
**3k (K)**	1	2
**Roflumilast**	2	1
**2h**	3	3
**2i**	3	2
**2o**	3	2
**4f**	3	2
**5d**	2	2
**7**	1	1
**10a**	4	2
**Rolipram**	1	2

Table S2. Average of hydrogen bond distance, m(Å), and average number of hydrogen bond interactions/pose (n/pose) between ligand and residues of the catalytic site in vacuum and with water.

	In vacuum		With water (14 mol H_2O)	
	m (Å)	n/pose	m (Å)	n/pose
Class A	2.84	3.81	2.89	4.59
Class I	2.75	3.7	2.87	3.5
p	0.265		0.716	

Table S3. Average of hydrogen bond distance, m(Å), and average number of hydrogen bond interactions/pose (n/pose) between ligand and water molecules.

	m (Å)	n/posa
Class A	2.64	1.47
Calss I	2.18	2.13
p	0.007	

Table S4. Grid parameters.

npts 60 60 60	# num.grid points in xyz
spacing 0.375	# spacing(A)
receptor_types A C Mg N NA OA SA Zn	# receptor atom types
ligand_types A C Cl NA OA N HD	# ligand atom types
gridcenter 49.4 46.7 64.3	# xyz-coordinates or auto
smooth 0.5	# store minimum energy w/in rad(A)
dielectric -0.1465	# <0, AD4 distance-dep.diel;>0, constant

Table S5. Lamarckian Genetic Algorithm parameters.

autodock_parameter_version 4.2	# used by autodock to validate parameter set
outlev 1	# diagnostic output level
intelec	# calculate internal electrostatics
seed pid time	# seeds for random generator
ligand_types A C Cl NA OA N HD	# atoms types in ligand
tran0 random	# initial coordinates/A or random
quaternion0 random	# initial orientation
dihe0 random	# initial dihedrals (relative) or random
torsdof 5	# torsional degrees of freedom
rmstol 2.0	# cluster_tolerance/A
extnrg 1000.0	# external grid energy
e0max 0.0 10000	# max initial energy; max number of retries
ga_pop_size 150	# number of individuals in population
ga_num_evals 2500000	# maximum number of energy evaluations
ga_num_generations 27000	# maximum number of generations
ga_elitism 1	# number of top individuals to survive
ga_mutation_rate 0.02	# rate of gene mutation
ga_crossover_rate 0.8	# rate of crossover
ga_window_size 10	#
ga_cauchy_alpha 0.0	# Alpha parameter of Cauchy distribution
ga_cauchy_beta 1.0	# Beta parameter Cauchy distribution
set_ga	# set the above parameters for GA or LGA
sw_max_its 300	# iterations of Solis & Wets local search
sw_max_succ 4	# consecutive successes before changing rho
sw_max_fail 4	# consecutive failures before changing rho
sw_rho 1.0	# size of local search space to sample
sw_lb_rho 0.01	# lower bound on rho
ls_search_freq 0.06	# probab. of performing local search on individual
set_psw1	# set the above pseudo-Solis & Wets parameters
unbound_model bound	# state of unbound ligand
ga_run 100	# do this many hybrid GA-LS runs
analysis	# perform a ranked cluster analysis

Table S5. More relevant GROMACS 5.1 parameters for minimization.

```
integrator = cg
emtol = 10.0
emstep = 0.01
nsteps = 50000
cutoff-scheme = group
nstlist      = 0
rlist = 0
ns_type = simple
coulombtype = Cut-off
vdw_type = Cut-off
rcoulomb = 0.0
rvdw = 0.0
pbc = no
```

Figure S1.

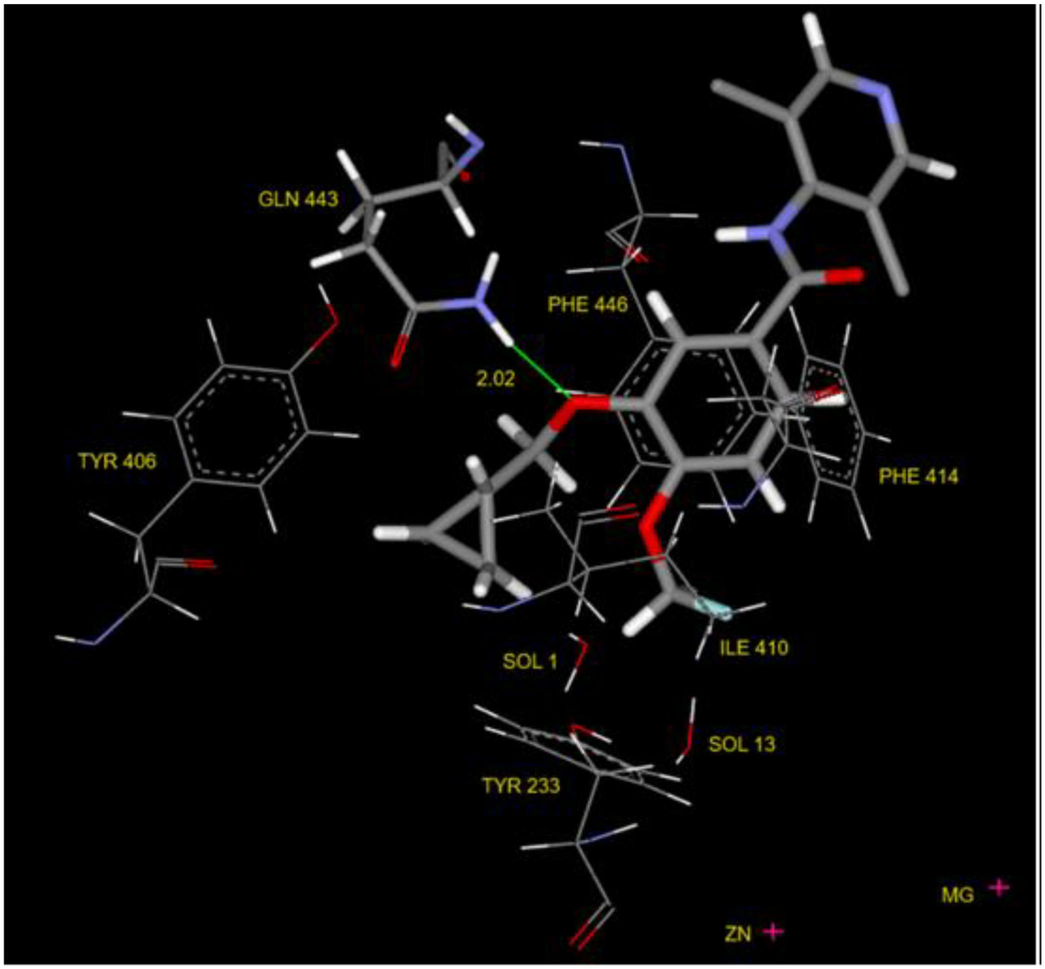

Minimized complex roflumilast-enzyme; the strong H-bond with Gln443 is evidenced

Figure S2.

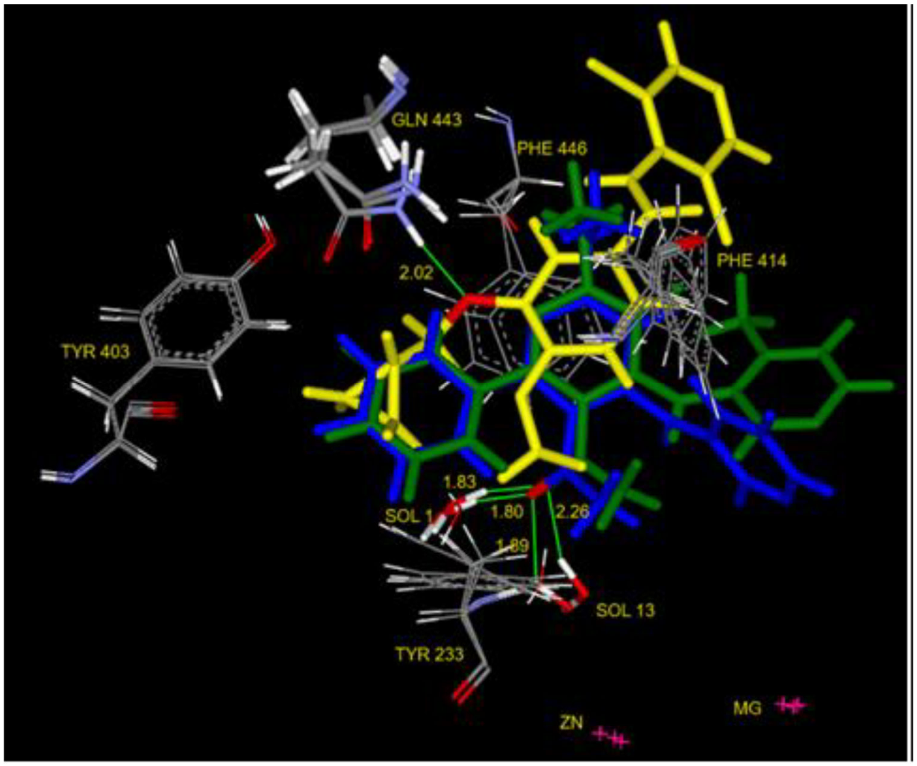

Roflumilast (yellow), **3a** (blu), **3k** (green)

Figure S3.

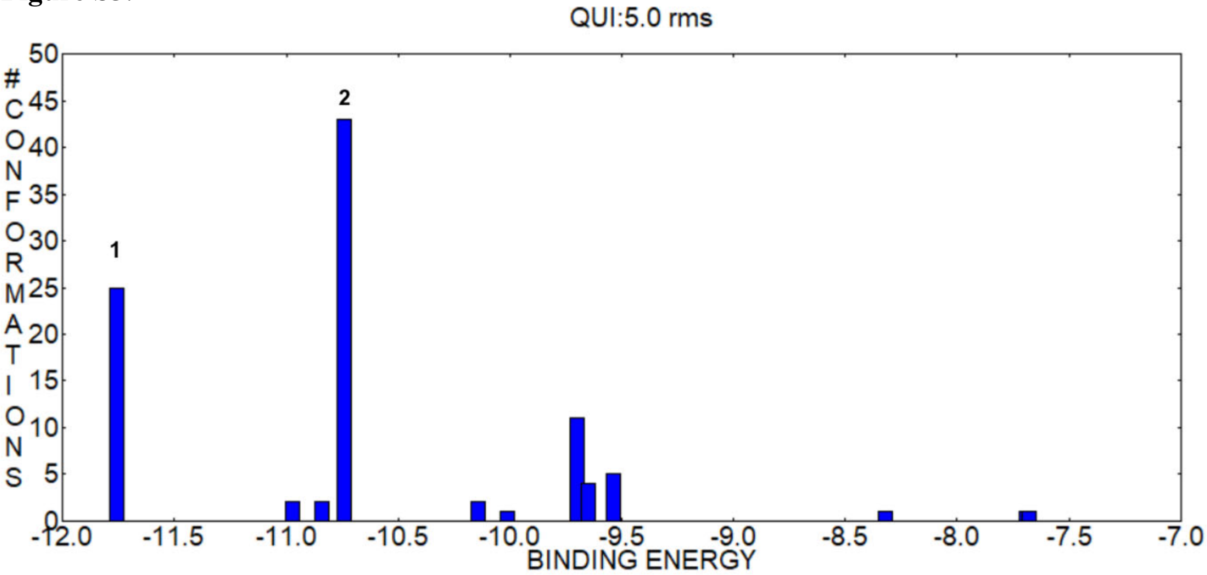

Figure S4.

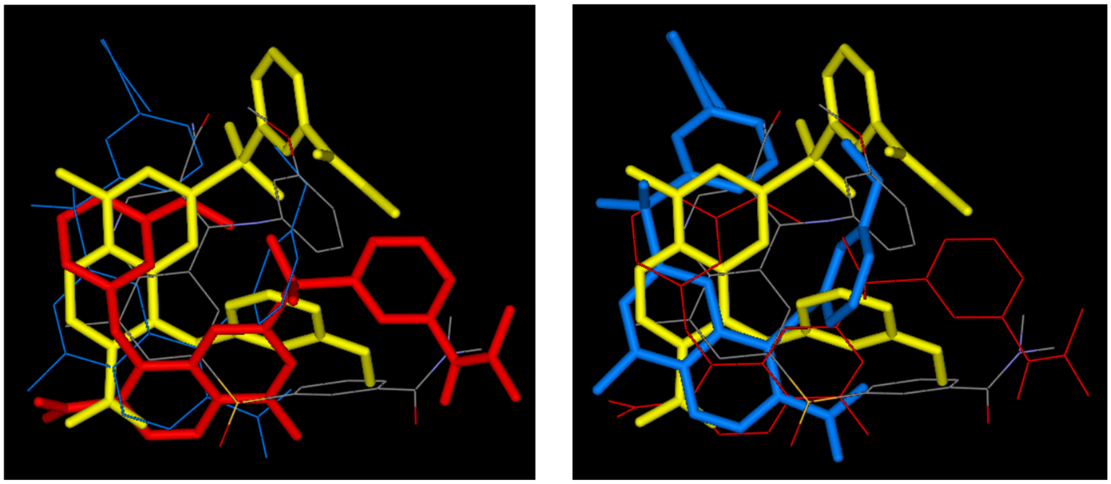

Figure S4, shows the pose of the crystallised ligand (yellow), the low-energy pose of cluster 1 (red), and the high-number conformation pose of cluster 2 (light blue) **Figure S3**. The sum of the two clusters accounts for approximately 70% of the calculated poses and sufficiently mimics the pose of the crystallised ligand. Given that the hydrogen-bond ligands reported in the paper were measured after complete relaxation of the enzyme-ligand complex, we consider the poses calculated by AutoDock acceptable.

4. **Elemental analyses. Table S6**

	Calculated			Found		
**Comp**	**%C**	**%H**	**%N**	**%C**	**%H**	**%N**
**2a**	72.05	5.74	12.60	72.33	5.76	12.65
**2b**	68.36	5.16	11.96	68.08	5.14	11.91
**2c**	65.31	4.93	11.42	65.05	4.91	11.37
**2d**	58.27	4.40	10.19	58.50	4.42	10.23
**2e**	69.41	5.82	11.56	69.69	5.84	11.60
**2f**	70.38	5.06	15.63	70.10	5.04	15.57
**2g**	62.84	4.52	10.47	62.59	4.50	10.43
**2h**	68.75	5.48	12.03	68.47	5.46	11.98
**2i**	66.83	5.07	11.13	66.56	5.05	11.08
**2j**	68.36	5.16	11.96	68.63	5.18	12.00
**2k**	69.41	5.82	11.56	69.69	5.84	11.60
**2l**	58.27	4.40	10.19	58.50	4.42	10.23
**2m**	69.41	5.82	11.56	69.13	5.80	11.51
**2n**	66.83	5.07	11.13	66.56	5.05	11.08
**2o**	68.13	5.72	10.36	68.40	5.74	10.40
**2p**	63.49	4.80	14.81	63.23	4.78	14.75
**2q**	70.38	5.06	15.63	70.66	5.08	15.69
**2r**	66.83	5.07	11.13	66.56	5.05	11.08
**2s**	63.40	5.07	10.56	63.65	5.09	10.60
**2t**	63.49	4.80	14.81	63.23	4.78	14.75
**2u**	65.31	4.93	11.42	65.05	4.91	11.37
**2v**	69.41	5.82	11.56	69.69	5.84	11.60
**2w**	65.03	4.64	11.38	65.29	4.66	11.42
**3a**	68.25	5.43	16.76	68.52	5.45	16.83
**3b**	71.86	5.24	14.57	71.57	5.22	14.51
**3c**	70.25	4.95	9.83	70.53	4.97	9.87
**3d**	72.17	5.30	10.52	72.43	5.32	10.56
**3e**	72.17	5.30	10.52	72.43	5.32	10.56
**3f**	71.86	5.24	14.57	71.57	5.22	14.51

**3g**	67.28	4.71	13.08	67.55	4.73	13.13
**3h**	68.99	5.03	13.99	68.71	5.01	13.93
**3i**	68.65	4.76	13.92	68.37	4.74	13.86
**3j**	68.99	5.03	13.99	68.71	5.01	13.93
**3k**	68.95	5.79	16.08	68.67	5.77	16.01
**3l**	75.18	5.52	10.96	75.48	5.54	11.00
**4a**	67.35	6.71	14.73	67.08	6.69	14.67
**4b**	68.20	7.07	14.04	68.47	7.09	14.10
**4c**	70.13	7.12	12.91	70.41	7.15	12.96
**4d**	70.77	7.42	12.38	70.49	7.39	12.43
**4e**	71.36	7.70	11.89	71.64	7.73	11.94
**4f**	70.35	7.97	12.31	70.63	7.99	12.36
**4g**	68.98	7.40	13.41	68.70	7.37	13.36
**5a**	70.01	6.14	11.13	70.29	6.16	11.17
**5b**	66.05	5.28	11.00	66.31	5.30	11.04
**5c**	68.39	6.46	9.97	68.11	6.44	9.93
**5d**	67.51	5.41	10.74	67.78	5.43	10.78
**5e**	70.01	6.14	11.13	70.29	6.16	11.17
**5f**	72.60	6.09	12.10	72.30	6.07	12.05
**5g**	68.95	5.79	16.08	68.67	5.77	16.01
**5h**	68.95	5.79	16.08	68.67	5.77	16.01
**5i**	68.95	5.79	16.08	68.67	5.77	16.01
**5j**	69.59	6.12	15.46	69.86	6.14	15.52
**5k**	64.57	5.42	11.89	64.31	5.40	11.84
**5l**	64.76	5.72	15.90	64.50	5.70	15.83
**7**	65.36	5.88	16.33	65.62	5.90	16.39
**9**	71.42	5.09	8.33	71.70	5.11	8.36
**10a**	68.55	5.18	7.99	68.27	5.16	7.96
**10b**	71.84	5.43	8.38	71.55	5.42	8.35

5. References

[1] Dal Piaz, V.; Giovannoni, M. P.; Castellana, C.; Palacios, J. M.; Beleta, J.; Domenech, T.; Segarra, V. Novel Heterocyclic-Fused Pyridazinones as Potent and Selective Phosphodiesterase IV Inhibitors. *J. Med. Chem.* **1997**, *40*, 1417-1421. DOI:10.1021/jm970105l.
